# Supplementary material for: Allele-selective and pan-allelic huntingtin lowering differentially affect neuronal homeostasis and BDNF transport
Source: Mol Ther Nucleic Acids. 2026 Oct 27;37(4):103160. doi: 10.1016/j.omtn.2026.103160 (PMC13770661; doi:10.1016/j.omtn.2026.103160)
Supplement: Supplementary file 1 [file mmc1.pdf]

OMTN, Volume *37*

Supplemental information

Allele-selective and pan-allelic huntingtin lowering differentially affect neuronal homeostasis and BDNF transport

Romane A. Lahaye, Aude Lemesle, Laurent Cotter, Donya El Akrouti, Virginie Redeker, Mathilde Louçã, Hélène Jean-Jacques, Yuanjing Liu, Abbie Maguire, Naoki Iwamoto, Michael Byrne, Chandra Vargeese, Sophie Lenoir, Frédéric Saudou, and Anselme L. Perrier

Table S1. Differentially expressed proteins identified by quantitative proteomic profiling following pan-allelic or allele-selective HTT ASO treatment in 109Q iPSC-derived cortical neurons.

See excel file Table S1.xlsx

Table S2. Antibodies used for immunostaining and Western blot

Target	Supplier	Application	Dilution	Reference
TBR1	Abcam	IF	1:500	ab31940
MAP2	Abcam	IF	1:500	ab5392
GABA	Sigma-Aldrich	IF	1:4000	A2052
cFos	Abcam	IF	1:500	2H2
EGR1	Cell Signaling	IF	1:500	15F7
GFAP	Millipore	IF	1:1000	AB5541
VLGUT2	Synaptic System	IF	1:500	135403
HOMER	Synaptic System	IF	1:500	160006
BASSOON	Abcam	IF	1:500	ab82958
Huntingtin	Cell Signaling	WB	1:1000	D7F7
Huntingtin	Ozyme	WB	1:1000	5656S
Calnexin	Sigma-Aldrich	WB	1:2500	C4731
GluA1	Millipore	WB	1:1000	AB1504
Vinculin	Cell Signaling	WB	1:10000	V9131
pAKT	Cell Signaling	WB	1:1000	40585
pERK	Cell Signaling	WB	1:2000	9106
AKT	Cell Signaling	WB	1:1000	9272
ERK	Cell Signaling	WB	1:1000	9102

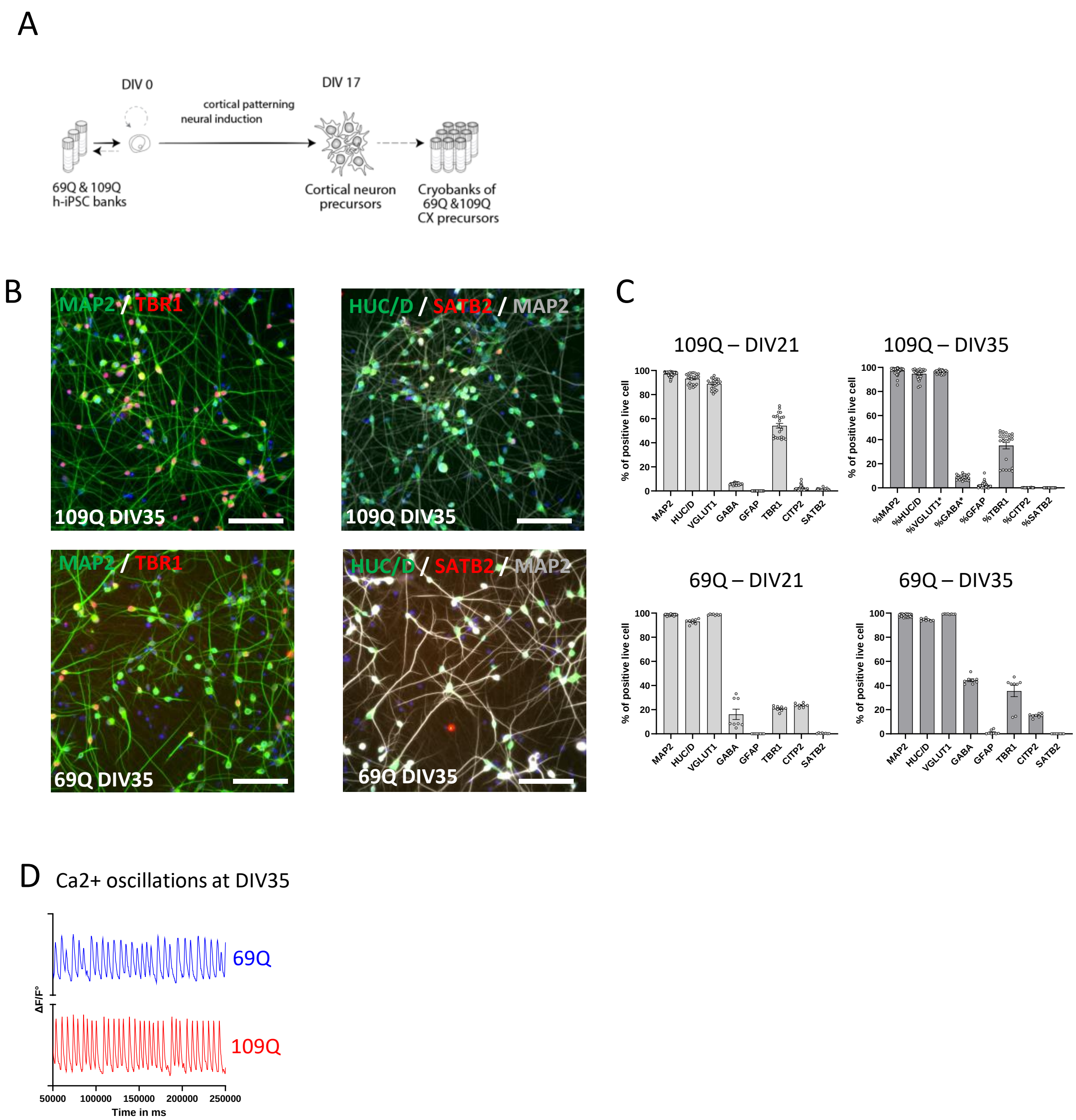

Figure S1. Characterization of human iPSC-derived neuronal population.

(A) Schematic of the differentiation. HD iPSCs carrying 69 or 109 CAG repeats were differentiated into cortical neurons.

(B) Immunostaining and (C) quantification of different markers of neuronal (MAP2, GABA), glial (GFAP), and cortical identities (TBR1) of 109Q iPSC- and 69Q-iPSC-derived cells at DIV21 and DIV35 after seeding of neuron precursor. Data are mean ± SEM; Scale bar: 50µm.

(D) Representative 200-second traces from the 10-min recordings of calcium oscillations in wells with 109Q or 69Q human cortical neuronal networks at DIV35 (synchronized calcium transient).

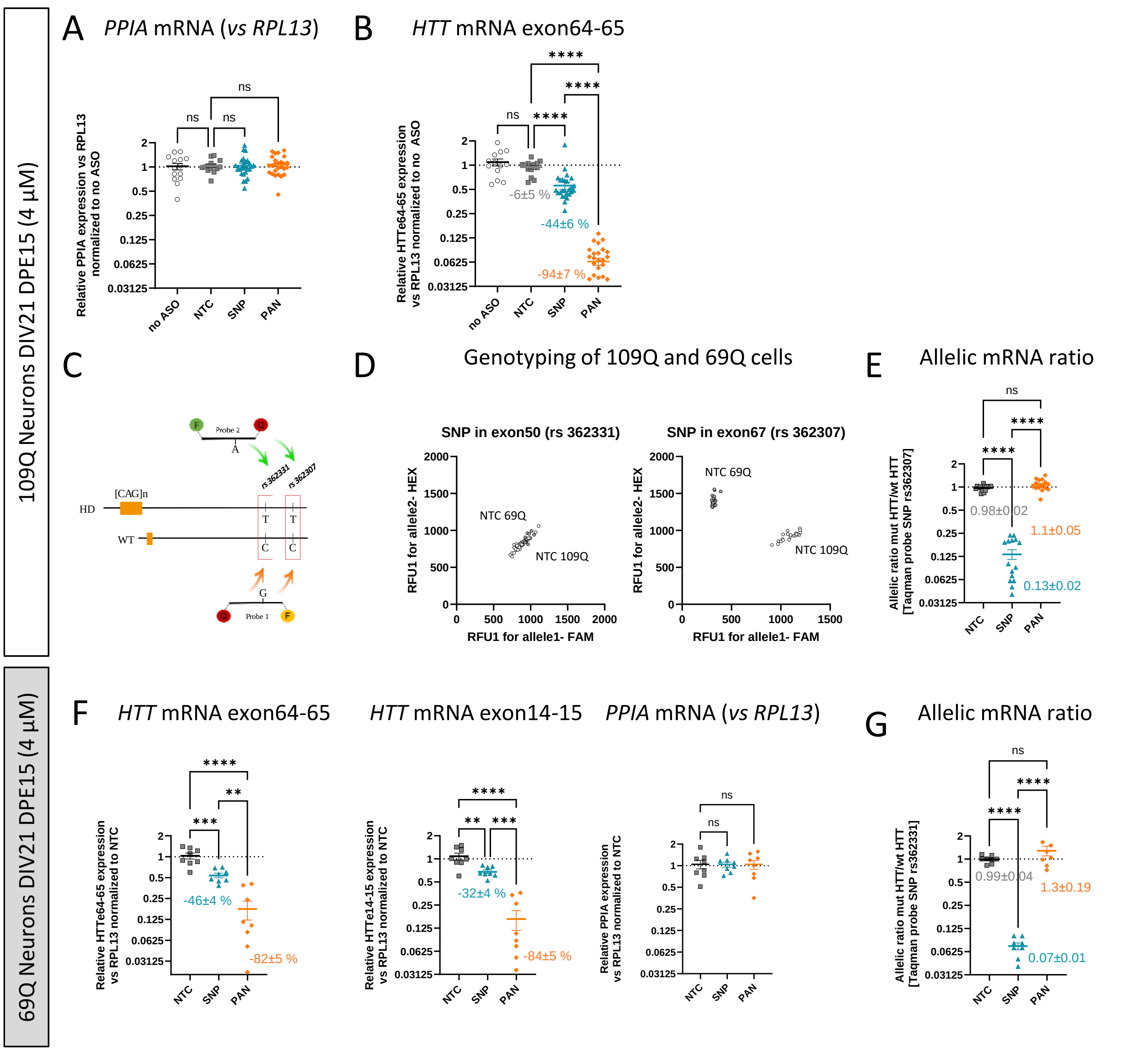

Figure S2. *HTT* mRNA-lowering efficacy and allele selectivity of ASOs in HD iPSC-derived cortical neurons.

(A) qRT–PCR analysis of *PPIA* relative to *RPL13* at DIV21–dpe15 showing stable expression of this second reference gene relative to *RPL13*.

(B) qRT–PCR analysis of spliced *HTT* transcripts (exons 64–65) at DIV21–dpe15 following treatment with NTC, PAN, or SNP ASOs.

(C) Schematic of TaqMan probe binding on both SNPs on either allele and allele-specific qPCR analysis.

(D) Genotyping of 109Q and 69Q lines for SNP rs362331 and rs362307. Distribution of the maximum fluorescence intensity of each probe after qRT-PCR of multiple 109Q and 69Q cDNA samples. Both 109Q and 69Q lines were heterozygous for SNP rs 362331, whereas only the 109Q line was heterozygous for SNP rs362307.

(E) Allelic ratio of mut- and wt-*HTT* mRNA in DIV21–dpe15 109Q neurons calculated based on the Ct difference of both probes for the same sample ($R_{mut/wt} = 2^{(-\Delta Ct)}$), normalized to the ratio of control samples.

(F) qRT–PCR analysis of spliced *HTT* transcripts (exons 14–15) at DIV21–dpe15 in 69Q neurons following treatment with NTC, PAN, or SNP ASOs. qRT–PCR analysis of *PPIA* relative to *RPL13* at DIV21–dpe15 in 69Q neurons.

(G) Allelic ratio of mut- and wt-*HTT* mRNA of DIV21–dpe15 neurons calculated based on the Ct difference of both probes for the same sample ($R_{mut/wt} = 2^{(-\Delta Ct)}$), normalized to the ratio of control samples.

Data are mean ± SEM; One-way ANOVA with post hoc test; p-values: *; <0.05; ** = 0.01; *** <0.001; **** < 0.0001.

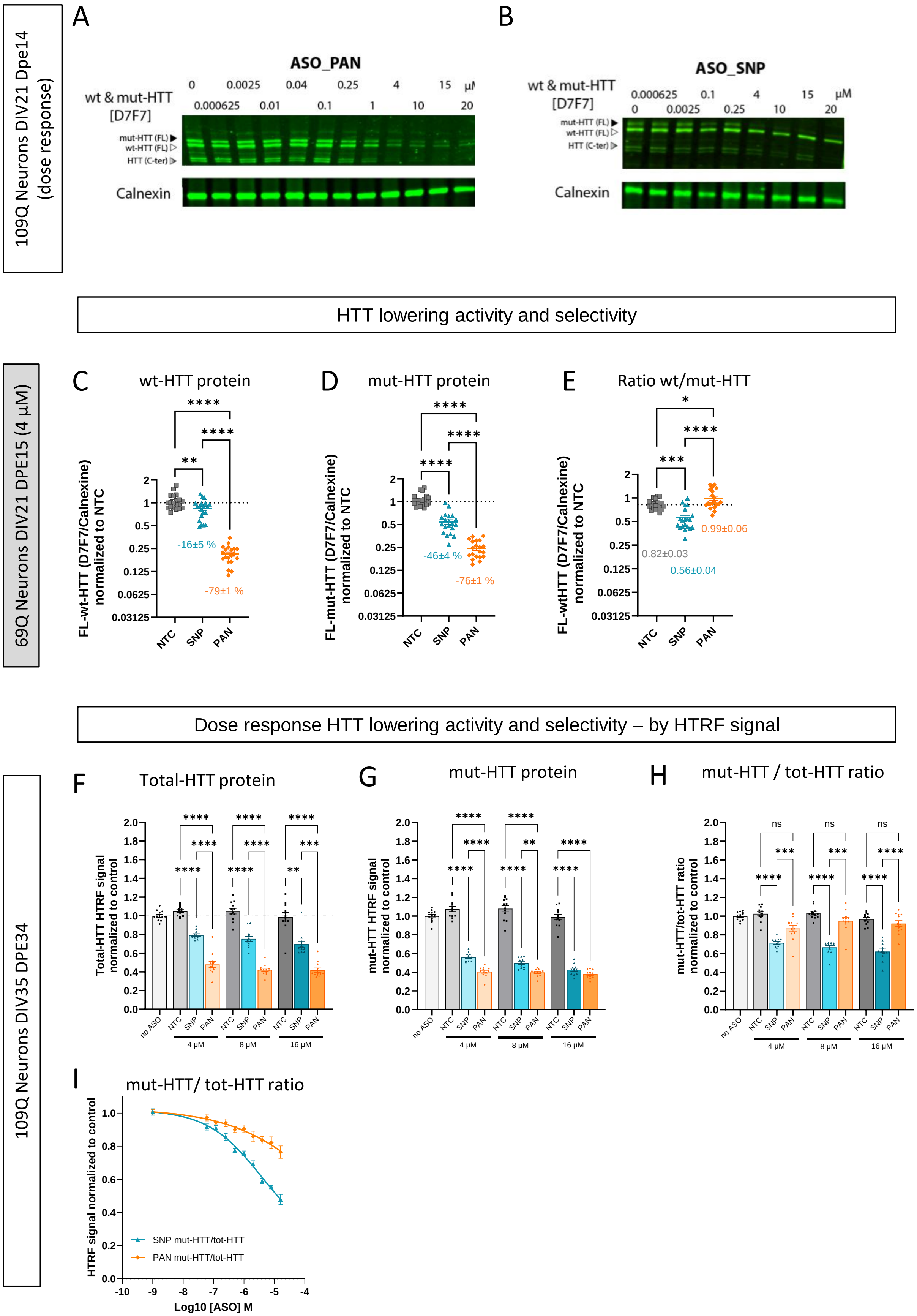
109Q Neurons DIV21 Dpe14 (dose response)
A
ASO_PAN
wt & mut-HTT [D7F7]
0 0.000625 0.0025 0.01 0.04 0.1 0.25 1 4 10 15 20 µM
mut-HTT (FL)
wt-HTT (FL)
HTT (C-ter)
Calnexin
B
ASO_SNP
wt & mut-HTT [D7F7]
0.000625 0 0.1 0.0025 0.25 4 10 15 20 µM
mut-HTT (FL)
wt-HTT (FL)
HTT (C-ter)
Calnexin
HTT lowering activity and selectivity
69Q Neurons DIV21 DPE15 (4 µM)
C
wt-HTT protein
FL-wt-HTT (D7F7/Calnexine) normalized to NTC
-16±5 %
-79±1 %
NTC
SNP
PAN
D
mut-HTT protein
FL-mut-HTT (D7F7/Calnexine) normalized to NTC
-46±4 %
-76±1 %
E
Ratio wt/mut-HTT
FL-wtHTT (D7F7/Calnexine) normalized to NTC
0.82±0.03
0.56±0.04
0.99±0.06
Dose response HTT lowering activity and selectivity – by HTRF signal
109Q Neurons DIV35 DPE34
F
Total-HTT protein
Total-HTT HTRF signal normalized to control
G
mut-HTT protein
mut-HTT HTRF signal normalized to control
H
mut-HTT / tot-HTT ratio
mut-HTT/tot-HTT ratio normalized to control
no ASO
NTC
SNP
PAN
4 µM
8 µM
16 µM
ns
I
mut-HTT/ tot-HTT ratio
HTRF signal normalized to control
SNP mut-HTT/tot-HTT
PAN mut-HTT/tot-HTT
Log10 [ASO] M

Figure S3. HTT full-length protein-lowering efficacy and allele selectivity of ASOs in HD iPSC-derived cortical neurons.

(A-B) Representative western blot images of wild-type and mutant full-length and C-terminal fragment of HTT protein (D7F7 antibody) and Calnexin in 109Q neurons (DIV21-dpe15) exposed once to 0-20 µM of PAN or SNP ASO.

(C-E) Western blots and quantification of full-length HTT protein (D7F7 antibody) normalized to Calnexin in 69Q neurons at DIV21-dpe15 following treatment with NTC, PAN, or SNP ASOs.

(F-I) HTRF assays for total and mut-HTT protein at DIV35–dpe34 of 109Q neurons exposed to 4, 8 or 16 µM of ASOs.

Data are mean ± SEM ; One-way ANOVA with post hoc test; p-values: *; <0.05; ** = 0.01; *** <0.001; **** < 0.0001.

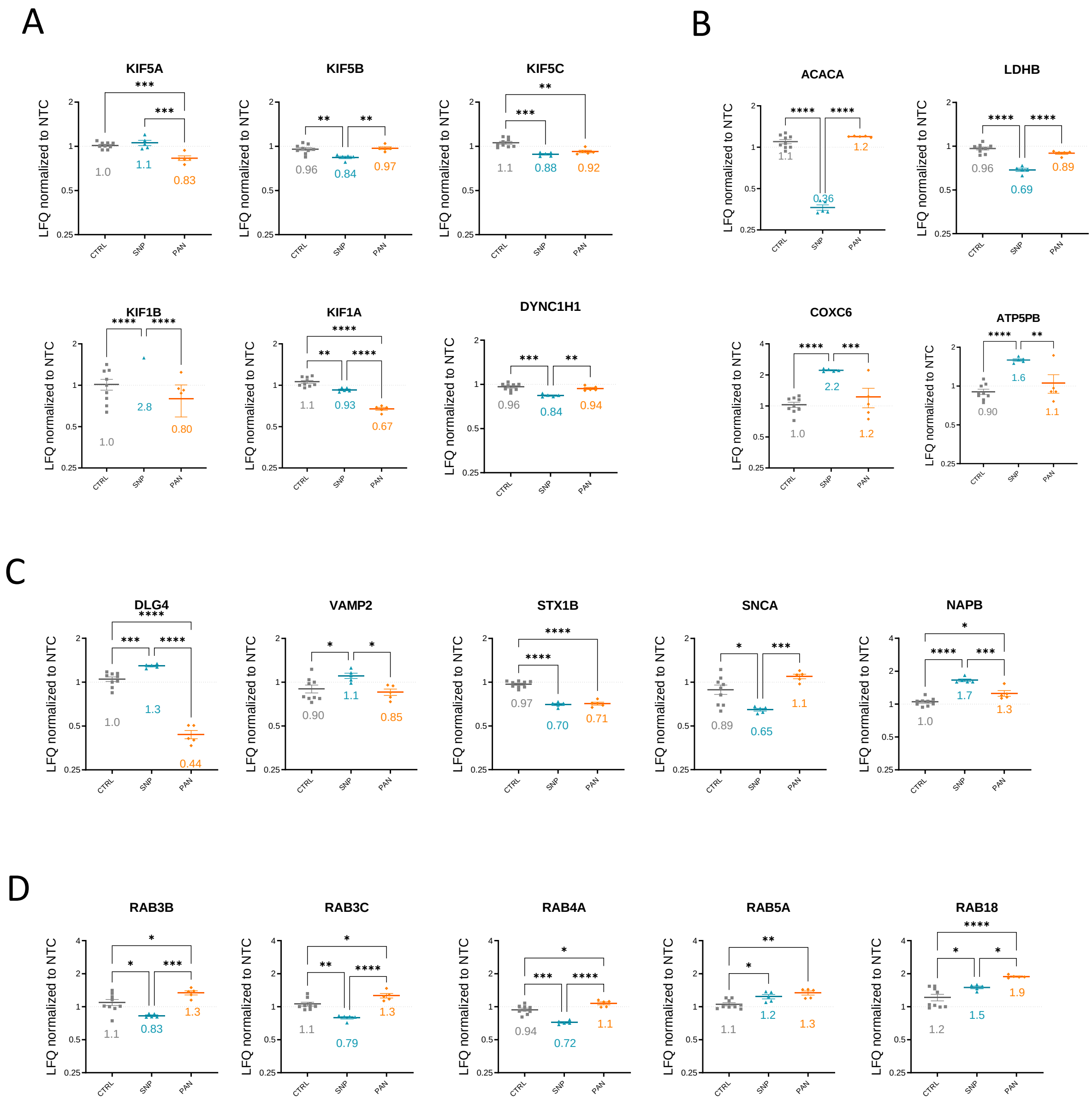

Figure S4. Proteomic signature of allele-selective versus pan-allelic HTT lowering in human HD cortical neurons.

Label free quantification signals normalized to NTC treated neurons for selected proteins involved in (A) cytoskeletal motors (KIF5A/B/C, KIF1A/B, DYNC1H1) essential for axonal transport, (B) metabolic enzymes (ACACA, LDHB, COX6C, ATP5PB) involved in ATP production, (C) synaptic proteins (DLG4/PSD-95, VAMP2, STX1B, SNCA, NAPB) mediating vesicle fusion and neurotransmission, and (D) Rab GTPases (RAB3B/C, RAB4A, RAB5A, RAB18) regulating vesicular trafficking. In the graphs, the condition labeled "CTRL" represents the combined control samples (NTC-treated and no-ASO), whereas normalization was performed exclusively relative to the NTC-treated group.

Data are mean ± SEM ; One-way ANOVA with post hoc test; p-values: *; <0.05; ** = 0.01; *** <0.001; **** < 0.0001.

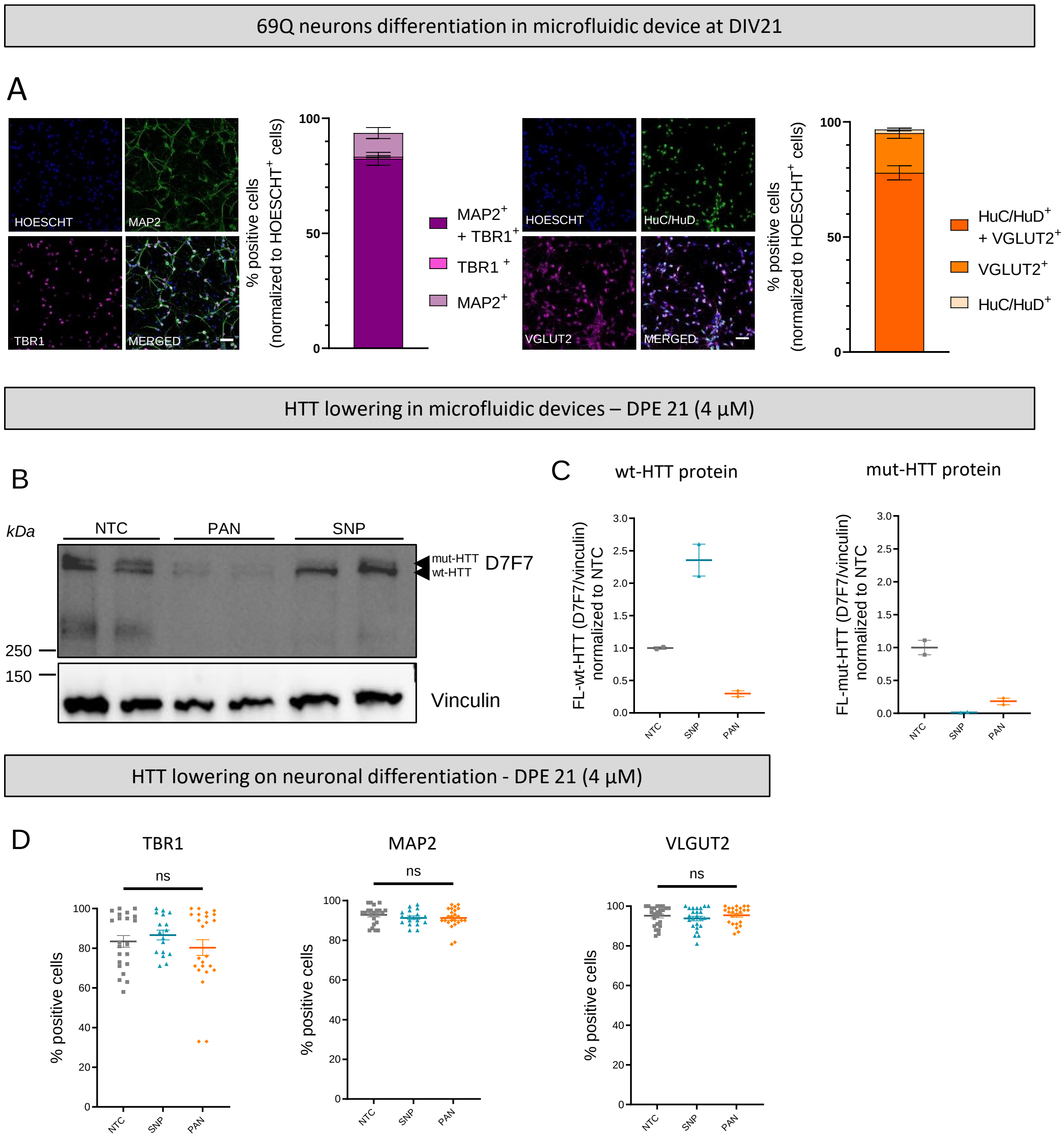

Figure S5. HTT silencing in human 69Q corticocortical neuronal networks-on-chip.

(A) Representative immunofluorescence images of 69Q iPSC-derived cortical neurons at DIV21, showing neuronal (HuC/D, MAP2) and cortical glutamatergic identity (TBR1, VGLUT2) markers.

(B-C) Western blot validation of *HTT* silencing following exposure of 69Q cells at DIV1 to 4 µM NTC, PAN or SNP ASOs for 20 days. Representative blots (B) and quantification of wt-HTT and mut-HTT (C) normalized to Vinculin.

(D) Quantification of $TBR1^+$, $MAP2^+$, and $VGLUT2^+$ populations.

Data are mean ± SEM; n = 3–4 independent networks per condition; one-way ANOVA with Tukey's post hoc test;

; p-values: *; <0.05; ** = 0.01; *** <0.001; **** < 0.0001. Scale bar: 50 µm.

BDNF containing vesicles traffic – 69Q Neurons DIV21 – Dpe 20 (4µM)

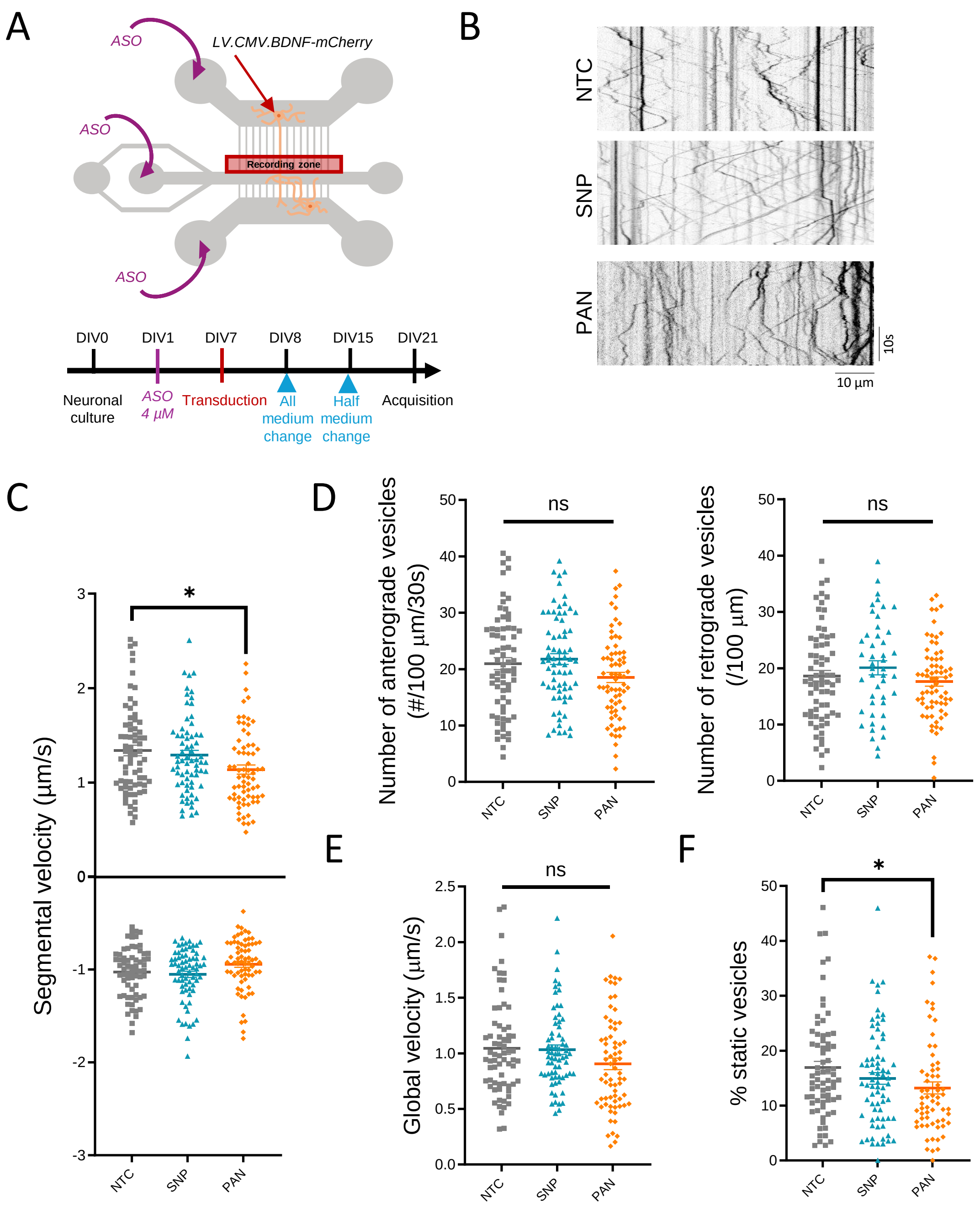

Figure S6. BDNF-mCherry vesicle transport following selective or pan-allelic HTT silencing .

(A) Schematic of the microfluidic device (MFD) used to reconstruct human corticocortical networks and experimental timeline. 69Q iPSC-derived cortical neurons were treated at DIV1 with 4 µM NTC, PAN or SNP ASOs. At DIV7, presynaptic 69Q iPSC–derived cortical neurons were transduced with lentiviral BDNF–mCherry. Live imaging of distal axons within 500-µm microchannels was performed at DIV21.

(B) Representative kymographs showing BDNF–mCherry vesicle trajectories in neurons treated with NTC, PAN, or SNP ASOs.

(C–F) Quantitative analyses of BDNF–mCherry transport. (C) Segmental velocities of anterograde and retrograde vesicles. (D) Number of anterograde and retrograde vesicles per 100 µm of axon. (E) Global vesicle velocity. (F) Percentage of static vesicles.

Data represent mean ± SEM from three independent experiments (≥58 axons per condition). One-way ANOVA followed by Tukey's post hoc test (E, anterograde velocity in C) or Kruskal–Wallis test followed by Dunn's multiple comparison (C, retrograde velocity; D; F); *$p < 0.05$, **$p < 0.01$.

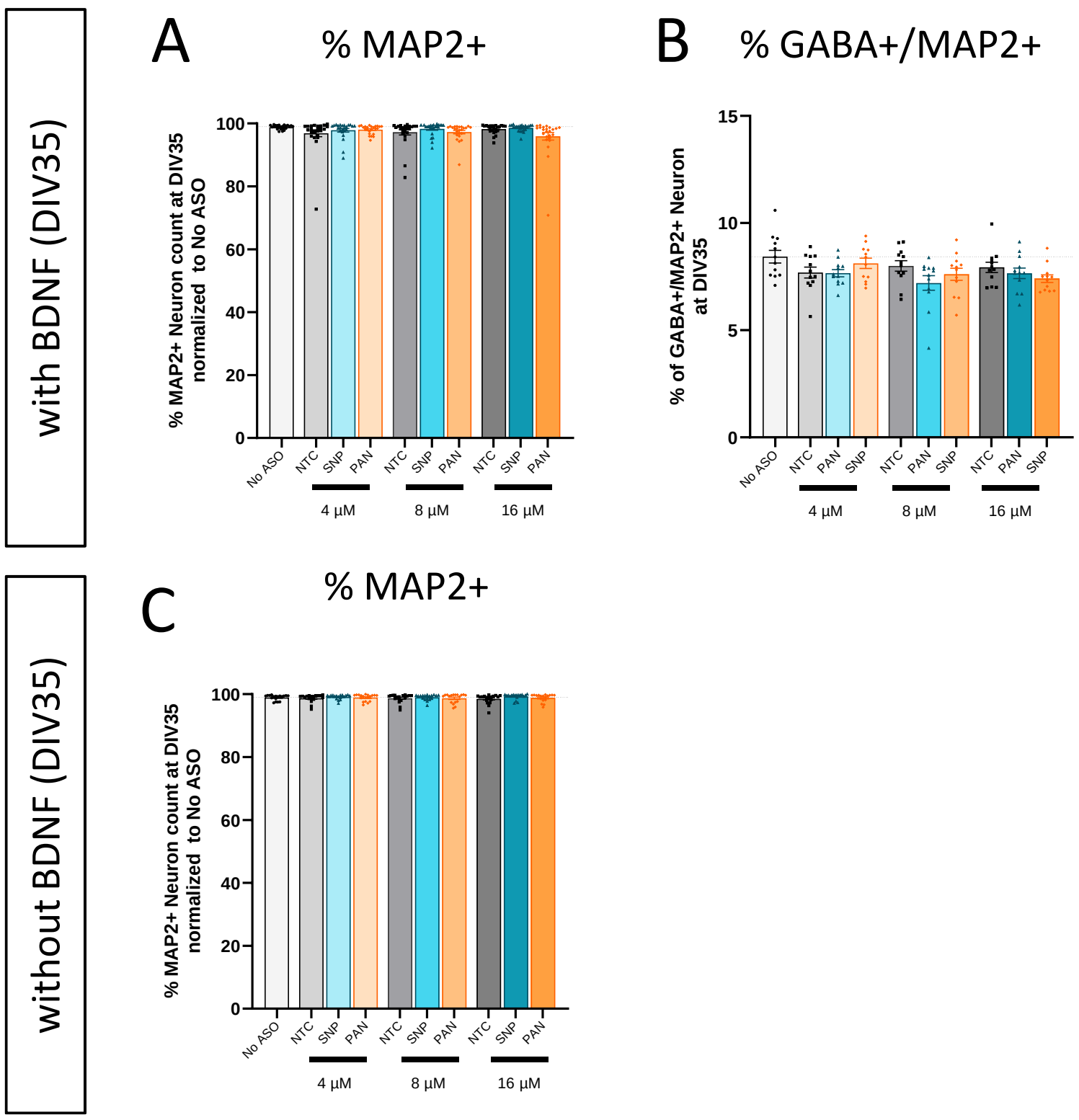

Figure S7. Effects of *HTT* silencing on neuronal subtype composition.

(A-B) Quantification of percentage of $MAP2^+$ and $GABA^+$ neurons in 109Q cortical cultures exposed once at DIV1 to different concentrations (0, 4, 8, 16 µM) of NTC (grey), SNP (teal), or PAN (orange) ASOs. Data are normalized to untreated controls. Cultures maintained with BDNF supplementation from DIV14 onward.

(C) Quantification of MAP2+ and GABA+ neurons in cultures without BDNF supplementation.

Data represent mean ± SEM from ≥3 independent experiments (n=6 cultures, 25 fields per condition). One-way ANOVA with Sidak's or Tukey's post hoc test.

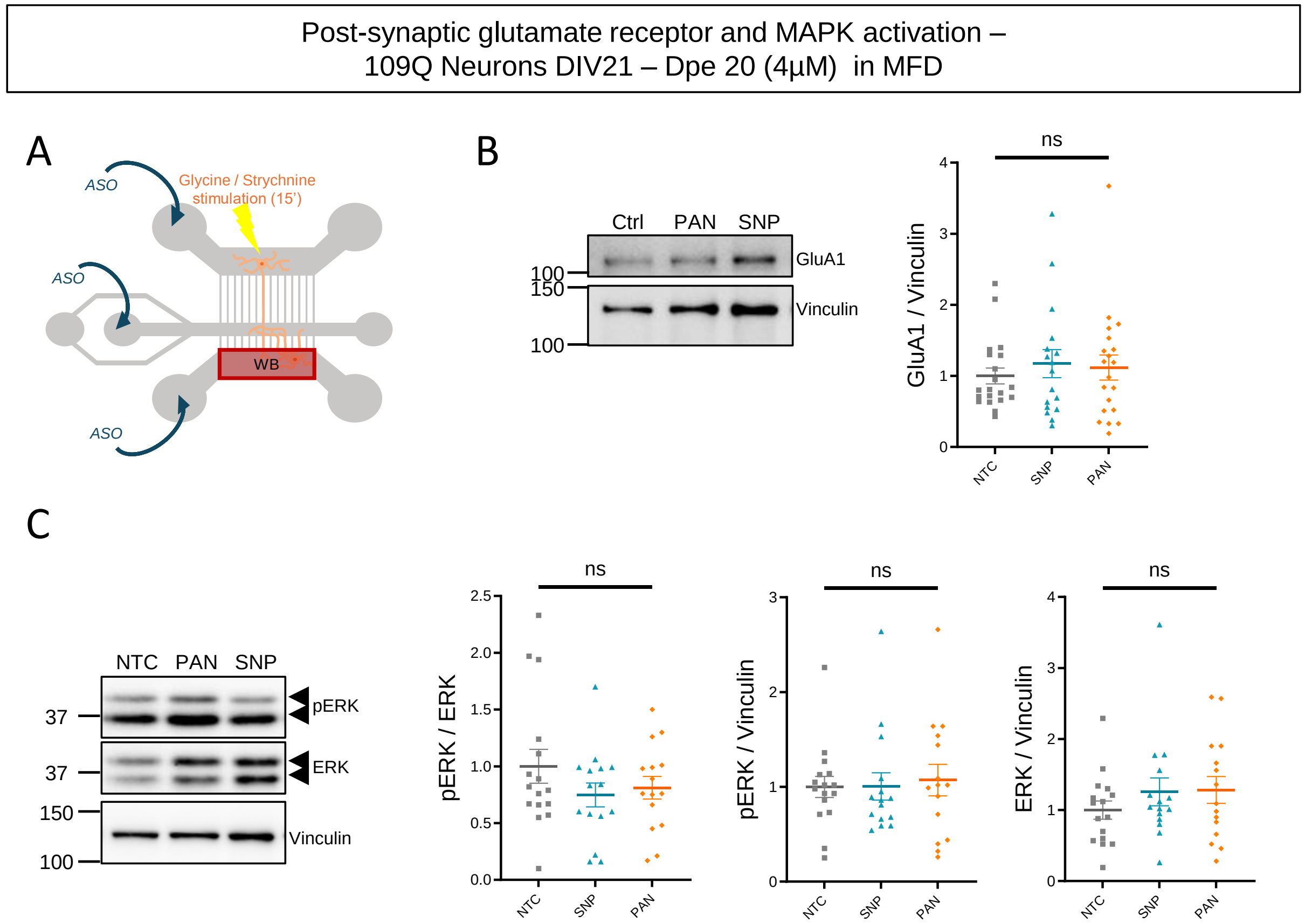

Figure S8. Postsynaptic glutamate receptor and MAPK signaling following selective or pan-allelic *HTT* silencing.

(A) Schematic of the microfluidic device (MFD) used to reconstitute human corticocortical networks, illustrating presynaptic stimulation with glycine/strychnine solution and postsynaptic protein extraction zone after 15 min stimulation performed at DIV21. Presynaptic neurons derived from 109Q iPSCs were treated at DIV1 with 4 µM NTC (grey), SNP (teal), or PAN (orange) ASOs.

(B) Western blots analysis of GluA1 expression normalized to Vinculin in 109Q neuronal networks treated with NTC, SNP, or PAN ASOs.

(C) Western blot analysis of pERK and total ERK expression normalized to Vinculin in 109Q neuronal networks treated with NTC, SNP, or PAN ASOs.

Each point represents protein extracts from one MFD (postsynaptic compartment only); four independent experiments, n = 12–16 per condition. Kruskal–Wallis or one-way ANOVA with appropriate post hoc tests; ns, not significant.

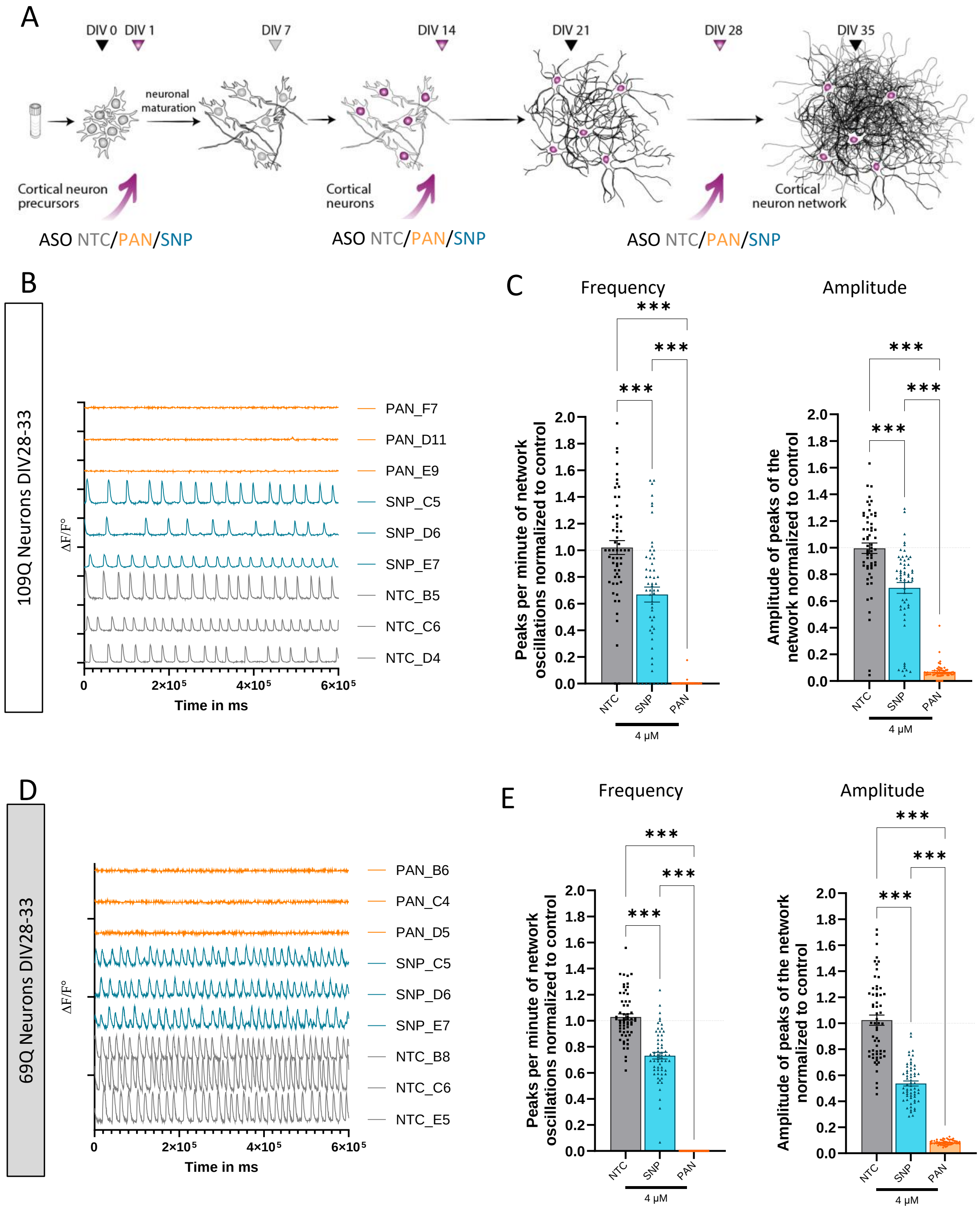
A
DIV 0
DIV 1
DIV 7
DIV 14
DIV 21
DIV 28
DIV 35
neuronal maturation
Cortical neuron precursors
Cortical neurons
Cortical neuron network
ASO NTC/PAN/SNP
ASO NTC/PAN/SNP
ASO NTC/PAN/SNP
B
109Q Neurons DIV28-33
ΔF/F°
PAN_F7
PAN_D11
PAN_E9
SNP_C5
SNP_D6
SNP_E7
NTC_B5
NTC_C6
NTC_D4
0
2×10⁵
4×10⁵
6×10⁵
Time in ms
C
Frequency
Amplitude
***
Peaks per minute of network oscillations normalized to control
Amplitude of peaks of the network normalized to control
NTC
SNP
PAN
4 µM
D
69Q Neurons DIV28-33
ΔF/F°
PAN_B6
PAN_C4
PAN_D5
SNP_C5
SNP_D6
SNP_E7
NTC_B8
NTC_C6
NTC_E5
0
2×10⁵
4×10⁵
6×10⁵
Time in ms
E
Frequency
Amplitude
***
Peaks per minute of network oscillations normalized to control
Amplitude of peaks of the network normalized to control
NTC
SNP
PAN
4 µM

Figure S9. Effects of HTT silencing on network synchronization across genotypes and repeated dosing paradigms.

(A) Schematic of the differentiation and repeated treatment workflow. Synchronized calcium transients in cortical neuronal networks derived from 109Q or 69Q human iPSCs after repeated exposure (DIV1, 14 and 28) to 4 µM NTC (grey), SNP (teal), or PAN (orange) ASOs. Network activity was recorded at DIV28 or DIV33 using Cal-520 fluorescence imaging.

(B & D) Representative 10 min traces of calcium oscillations from 109Q and 69Q neuronal networks.

(C & E) Quantification of normalized oscillation frequency and amplitude.

Data represent mean ± SEM from three independent experiments (20 cultures wells per condition); one-way ANOVA with Sidak's multiple comparisons; *$p < 0.05$, **$p < 0.01$, ***$p < 0.001$.

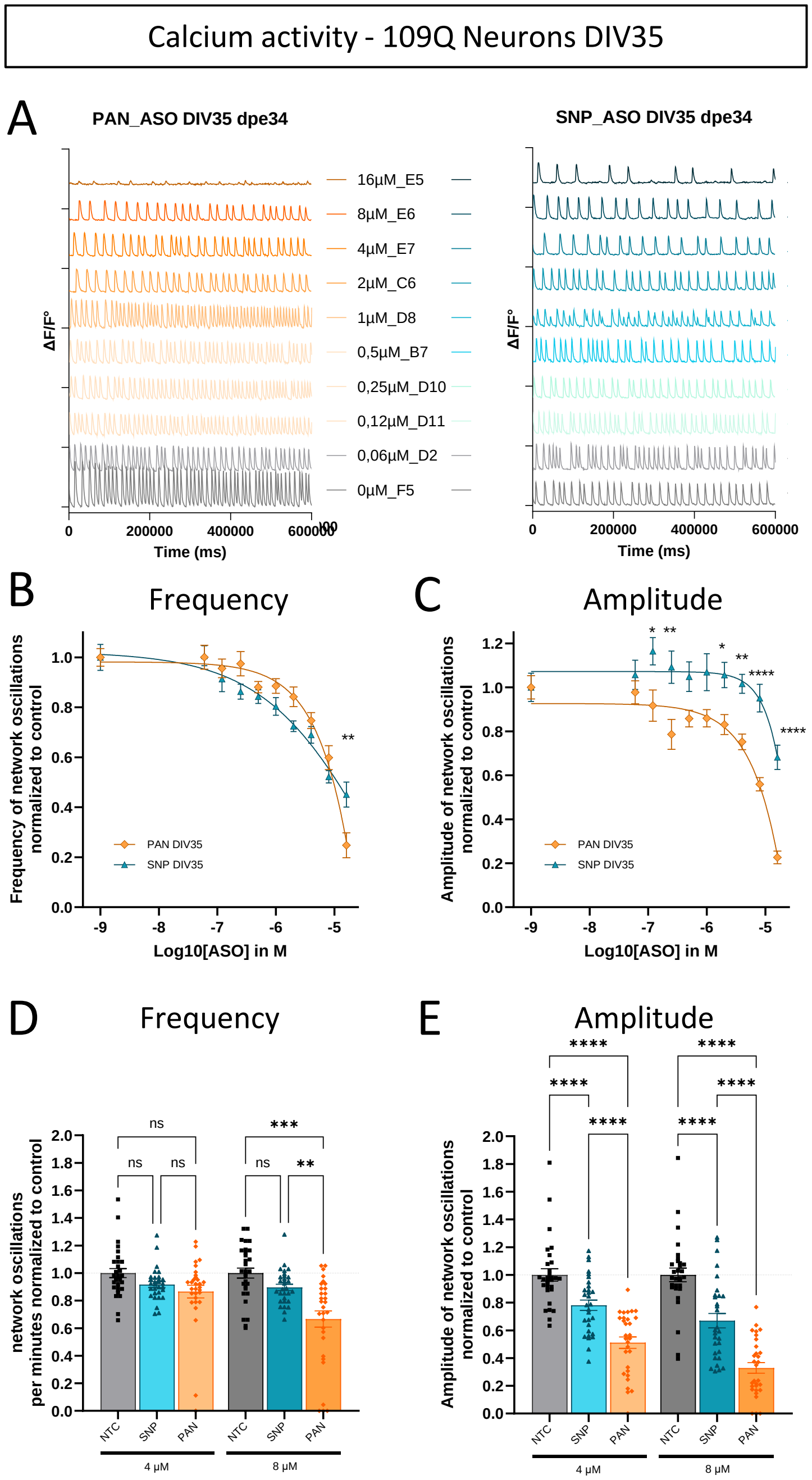

Figure S10. Sustained network activity following selective or pan-allelic *HTT* silencing.

(A–E) Spontaneous calcium oscillations in 109Q cortical networks recorded at DIV35 following a single exposure to 0–16 µM NTC (grey), SNP (teal), or PAN (orange) ASOs at DIV1.

(A) Representative 10 min traces of calcium oscillations.

(B & C) Dose–response curves showing normalized frequency (B) and amplitude (C) of network oscillations.

(D & E) Quantification at 4 µM and 8 µM ASOs relative to NTC. Despite partial loss of SNP ASO efficacy at this time point, network oscillations remained significantly more preserved than with PAN ASO treatment.

Data represent mean ± SEM from three independent experiments (six wells per condition); one-way ANOVA with Sidak's multiple comparisons; *$p < 0.05$, **$p < 0.01$, ***$p < 0.001$, ****$p<0.0001$***$p < 0.001$.
