# Supplementary material for: Allele-selective and pan-allelic huntingtin lowering differentially affect neuronal homeostasis and BDNF transport
Source: Mol Ther Nucleic Acids. 2026 Oct 27;37(4):103160. doi: 10.1016/j.omtn.2026.103160 (PMC13770661; doi:10.1016/j.omtn.2026.103160)
Supplement: Supplementary file 2 [file mmc3.pdf]

Molecular Therapy
Nucleic Acids
Original Article

Allele-selective and pan-allelic huntingtin lowering differentially affect neuronal homeostasis and BDNF transport

Romane A. Lahaye,[1,6] Aude Lemesle,[2,3,6] Laurent Cotter,[2,3,6] Donya El Akrouti,[2,3] Virginie Redeker,[3,4] Mathilde Louçã,[2,3] Hélène Jean-Jacques,[4] Yuanjing Liu,[5] Abbie Maguire,[5] Naoki Iwamoto,[5] Michael Byrne,[5] Chandra Vargeese,[5] Sophie Lenoir,[1] Frédéric Saudou,[1,7] and Anselme L. Perrier[2,3]

[1]Univ. Grenoble Alpes, INSERM U1216, CHU Grenoble Alpes, Grenoble Institut Neurosciences, 38000 Grenoble, France; [2]Université Paris-Saclay, CEA, Molecular Imaging Research Center, Laboratory of Molecular Mechanisms of Proteinopathies: Induced Pluripotent Stem Cells and Aptamers, 92265 Fontenay-aux-Roses, France; [3]Université Paris-Saclay, CEA, CNRS, Laboratoire des Maladies Neurodégénératives: Mécanismes, Thérapies, Imagerie, 92265 Fontenay-aux-Roses, France; [4]Université Paris-Saclay, CNRS, CEA, Institute for Integrative Biology of the Cell, 91198 Gif-sur-Yvette, France; [5]Wave Life Sciences, Cambridge, MA 02138, USA

Huntington's disease (HD) is a devastating genetic neurodegenerative disorder caused by the abnormal expansion of CAG-repeats, which encode a poly-glutamine stretch in the huntingtin (HTT) protein. HD patients typically carry one wild-type and one mutant allele. We compared pan-allelic (PAN) and allele-selective antisense oligonucleotides (ASOs) targeting a single-nucleotide polymorphism (SNP) in the sequence encoding the mutant isoform in human iPSC-derived cortical neurons carrying 69Q or 109Q alleles. SNP ASO selectively reduced mut-*HTT* mRNA and protein, while PAN ASO lowered both alleles. Proteomic profiling revealed that allele-selective mutant *HTT* lowering preserved pathways for neuronal morphogenesis, synaptic function, and mitochondrial metabolism. In human HD neuronal networks reconstituted in microfluidic devices, SNP ASO maintained synaptic connectivity, network synchronization, and BDNF vesicle trafficking. By contrast, PAN ASO impaired axonal transport, energy metabolism, activity-dependent survival signaling, and network oscillations. These findings support the rationale for allele-selective *HTT* silencing to preserve neuronal functions during HTT lowering while limiting extensive depletion of total HTT.

INTRODUCTION

Huntington's disease (HD) is a progressive, autosomal-dominant neurodegenerative disorder characterized by progressive motor, cognitive, and psychiatric impairments that arise from widespread striatal and cortical neuronal dysfunction and loss.[1] A major clinical challenge is the absence of an effective neuroprotective therapy to prevent disease onset or slow its progression.[2]

HD is caused by an expanded CAG trinucleotide repeat in one allele of the huntingtin (*HTT*) gene, producing mutant huntingtin (mut-HTT). This mutant protein carries an elongated polyglutamine tract in its N-terminal region, which promotes abnormal folding and aggregation, disrupts normal protein-protein interactions, and ultimately induces neuronal dysfunction and death.[3] Mut-HTT impairs intracellular trafficking, synaptic signaling, mitochondrial function, and transcriptional regulation, triggering a complex cascade of cellular stress that culminates in neuronal degeneration.[4–6]

By contrast, wild-type huntingtin (wt-HTT) performs multiple essential cellular roles that support neuronal viability and connectivity.[7] HTT acts as a scaffold for molecular motors that transport brain-derived neurotrophic factor (BDNF)-containing vesicles along microtubules. BDNF is critical for neuronal survival and is not synthesized in the striatum; most of striatal BDNF is supplied via cortical afferents.[8–10] Accordingly, wt-HTT is required for proper axonal and vesicular transport, BDNF transcription and trafficking, and protection against excitotoxic and apoptotic stress.[5,11–13]

Given these neuroprotective functions, HTT-lowering strategies must balance efficient reduction of mut-HTT, to remove its toxic dominant and potential dominant-negative effects, with the preservation of wt-HTT physiological functions.[7] Antisense oligonucleotides

Received 7 July 2026; accepted 23 October 2026;
https://doi.org/10.1016/j.omtn.2026.103160.

[6]These authors contributed equally

[7]Present address: Université de Strasbourg, CNRS, UMR7104, Inserm, U1258, Institut de Génétique et de Biologie Moléculaire et Cellulaire, IGBMC, 67400 Illkirch, France

Correspondence: Frédéric Saudou, Univ. Grenoble Alpes, INSERM U1216, CHU Grenoble Alpes, Grenoble Institut Neurosciences, 38000 Grenoble, France.
E-mail: frederic.saudou@inserm.fr
Correspondence: Anselme L. Perrier, Université Paris-Saclay, CEA, Molecular Imaging Research Center, Laboratory of Molecular Mechanisms of Proteinopathies: Induced Pluripotent Stem Cells and Aptamers, 92265 Fontenay-aux-Roses, France.
E-mail: anselme.perrier@cea.fr

Published by Elsevier Inc. on behalf of The American Society of Gene and Cell Therapy.
This is an open access article under the CC BY license (http://creativecommons.org/licenses/by/4.0/).

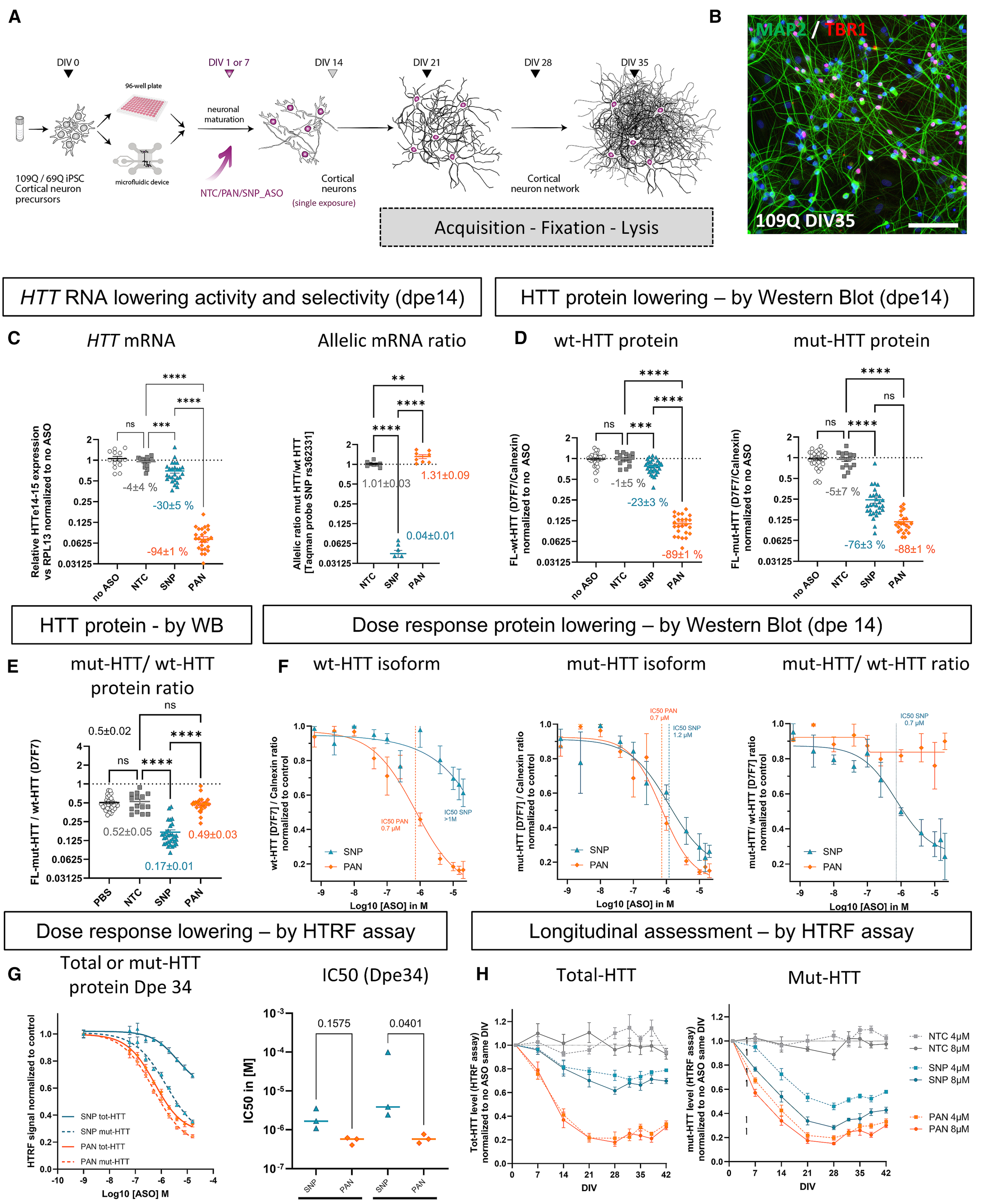

(legend on next page)

(ASOs) and adeno-associated virus (AAV)-mediated expression of micro ribonucleic acid (miRNA) have emerged as promising approaches to lower HTT in the brain of HD patients.[14] Pan-allelic (PAN) ASOs and miRNAs non-selectively reduce both mutant and wild-type *HTT* transcripts and have demonstrated robust suppression of HTT in the central nervous system in early clinical studies[15,16] However, sustained reduction of wt-HTT may be deleterious to neuronal health, supporting the development of allele-selective approaches.[17,18] Several non-selective strategies have produced adverse effects that led to trial discontinuations or unfavorable risk-benefit assessments[16] (clinicaltrials.gov NCT03761849). While some toxicity has been attributed to ASO dosing and repeated intrathecal administration, the branaplam trial discontinuation raises the possibility that reduced expression of wt-HTT may also contribute to adverse outcomes (clinicaltrials.gov NCT05111249[19]). Notwithstanding these negative results, several other clinical trials designed to reduce the expression of both alleles (e.g., NCT04120493, uniQure) are ongoing. Together, these mixed reports and the high number of ongoing strategies that reduce global HTT warrant cautious examination of the consequences of mut-HTT versus global HTT silencing on HTT function and neuroprotective pathways in human neurons.

We have previously shown that the selective lowering of mut-*HTT* with ribonucleic acid (RNA) interference restores BDNF transport in human cortical axons,[20] and there is evidence that restoring this BDNF supply to the striatum is neuroprotective.[21–26] Recent developments of allele-selective ASOs that target single-nucleotide polymorphisms (SNPs) linked to the mut-*HTT* allele enable preferential suppression of mut-HTT and have the potential to limit toxicity.[27–29]

Human-induced pluripotent stem cell (iPSC)-derived neuronal models provide a powerful platform to evaluate HTT-lowering strategies within a human genetic context. Patient-derived cortical neurons recapitulate key disease-relevant phenotypes, including disrupted synaptic signaling, impaired energy metabolism, and defective axonal transport.[30–33] In addition, we have shown that loss of wt-HTT in this human iPSC (hiPSC) model leads to detrimental effects on the maturation and function of healthy neurons, such as reduced dendritic arborization and impaired synaptic homeostasis, which compromise the development of synchronized, network-wide synaptic activity.[18,34] These findings provide a reference for interpreting the consequences of PAN versus selective mut-HTT lowering in our HD neuronal models.

In this study, we performed a systematic comparison of PAN and allele-selective HTT-lowering ASOs in hiPSC-derived cortical neurons from HD patients carrying 109 or 69 CAG repeats. By integrating molecular, proteomic, and functional analyses including long-term HTT silencing, vesicular BDNF trafficking, metabolic profiling, and neuronal network synchronization, we delineate the differential consequences of non-selective versus allele-selective silencing on human cortical neuronal homeostasis.

RESULTS

HTT-lowering activity and selectivity of PAN and allele-selective ASOs in human HD cortical neurons

To evaluate whether selective suppression of mut-HTT can ameliorate pathogenic mechanisms in HD, we generated cortical neurons from HD patient-derived iPSCs carrying 109 or 69 CAG repeats (Figure S1A). Differentiation toward a dorsal forebrain fate was achieved using dual SMAD inhibition followed by SHH inhibition and exposure to FGF2 and Wnt modulators.[18,34] We compared two ASOs (Wave Life Sciences): a PAN ASO targeting the exon 1-intron 1 junction of *HTT*, and an allele-selective ASO (SNP) directed against a SNP unique to the mut-*HTT* transcript (Figure 1A). This stereopure ASO incorporates phosphoryl guanidine (PN) linkages to enhance stability and promote selective RNase H-mediated degradation of mut-*HTT* transcripts.[27,35,36] Patient-specific iPSC-derived cortical neurons exhibited mature cortical identity and network functionality by day *in vitro* 21 (DIV21) (HuC/HuD^+ 98%, $MAP2^+$ 99%, $TBR1^+$ 43%, $CTIP2^+$ 2.5%; $VGLUT1^+$>80%; $GFAP^+$<3%; Figure 1B; Figures S1B and S1C). Calcium imaging confirmed synchronized oscillatory neuronal network activity consistent with mature excitatory-inhibitory coupling of the neurons (Figure S1D).[18]

Neurons were treated gymnotically at DIV6 with 4 μM PAN, SNP, or non-targeting control (NTC) ASO and analyzed 15 days post-exposure (DIV21-dpe15). Quantitative reverse-transcription polymerase chain reaction (RT-qPCR) quantification of spliced *HTT* mRNA using exon-spanning primers (exons 14–15, 64–65) showed a ∼90% reduction with PAN ASO in both 109Q and 69Q neurons (Figure 1C). As expected, SNP ASO, targeting only one allele, produced a smaller reduction (∼40%–50%). Genotyping and phasing of two transcribed SNPs in the *HTT* coding sequence (rs362331 in exon 50; rs362307 in exon 67) showed that both 109Q and 69Q iPSC lines were heterozygous at rs362331 (T on the mut-HTT allele and C on the wt-HTT allele), while only the 109Q

Figure 1. HTT-lowering efficacy and allele selectivity of ASOs in HD iPSC-derived cortical neurons

(A) Schematic of the differentiation and treatment workflow. HD iPSCs carrying 69 or 109 CAG repeats were differentiated into cortical neurons and treated gymnotically with non-targeting control (NTC), pan-allelic (PAN), or allele-selective (SNP) ASOs at DIV1 or 6. (B) Immunostaining at DIV35 for mature cortical neuronal markers ($MAP2^+$/$TBR1^+$). (C) RT-qPCR analysis of spliced *HTT* transcripts (exons 14–15) at DIV21-dpe15 in 109Q neurons following treatment with NTC, PAN, or SNP ASOs. Allele-specific RT-qPCR was used to assess the mut-*HTT*/wt-*HTT* transcript ratio. (D and E) Western blots and quantification of full-length HTT protein (D7F7 antibody) normalized to Calnexin in 109Q neurons. (F) Dose-response western blots analysis of HTT protein levels following treatment with PAN or SNP ASOs. IC_{50} values were 0.7 μM for PAN ASO for both isoforms and 1.2 μM for SNP ASO for mut-HTT. (G) HTRF assays at DIV35-dpe34 of HTT protein levels following treatment with PAN or SNP ASOs. (H) Longitudinal analysis of HTT suppression over approximately 4 weeks post-treatment.

Data are mean ± SEM; one-way ANOVA with post hoc test; *p* values: $^{**}p = 0.01$; $^{****}p < 0.0001$. Scale bars, 50 μm.

line was heterozygous at rs362307 (T on the mut-HTT allele and C on the wt-HTT allele) (Figures S2A–S2D). Allelic ratio measured using these SNPs confirmed ASOs allele selectivity, as the mutant/wild-type *HTT* mRNA ratio dropped to 0.04–0.10 in SNP ASO-treated neurons while remaining above 1 in PAN ASO-treated neurons (Figure 1C; Figure S2).

At the protein level, western blotting (D7F7 antibody) revealed parallel reductions in full-length HTT. PAN ASO decreased both wt-HTT and mut-HTT by over 80% (wt-HTT_69Q = −79% ± 1% and wt-HTT_109Q = −89% ± 1%; mut-HTT_69Q = −76% ± 1% and mut-HTT_109Q = −88% ± 1%), whereas SNP ASO reduced mut-HTT more strongly (mut-HTT_69Q = −46% ± 4% to mut-HTT_109Q = −76% ± 3%) while sparing wt-HTT (wt-HTT_69Q = −16% ± 5% to wt-HTT_109Q = −23% ± 3%) in both 109Q and 69Q neurons (Figure 1D; Figures S3A–S3D). Consequently, the mut-HTT/wt-HTT protein ratio was stable under PAN ASO but markedly decreased under SNP ASO treatment (R_{109Q} = 0.17 ± 0.01; R_{69Q} = 0.56 ± 0.04; $p < 0.001$; Figure 1E; Figure S3E). Dose-response analyses established IC_{50} values of 0.7 μM for PAN ASO across both isoforms, and 1.2 μM for SNP ASO selectively reducing mut-HTT (Figure 1F). Homogenous time-resolved fuorescence (HTRF) quantification of total and mut-HTT in neurons at DIV35-dpe34 confirmed comparable potency and revealed a gradual decline in maximal activity over time, consistent with waning ASO effect after one month (Figure 1G; Figures S3F–S3I). Longitudinal profiling after single exposure to ASOs showed progressive HTT suppression over 2–3 weeks, reaching >80% of maximal effect and remaining below this level up to ∼dpe41 (Figure 1H).

Together, these results demonstrate that PAN ASO mediate potent, non-selective HTT suppression, whereas SNP ASO preferentially reduces mut-HTT with significant preservation of wild-type HTT protein. Allele-selective silencing develops gradually and remains sustained for at least one month after a single exposure, validating allele-selective HTT lowering in human HD cortical neurons.

PAN- and SNP-ASO-mediated HTT lowering in HD human cortical neurons induces specific proteomic signatures

To define the molecular consequences of allele-selective versus PAN HTT lowering, we performed quantitative proteomic comparison of 109Q iPSC-derived cortical neurons exposed once at DIV6 to 4 μM of PAN, SNP, or NTC ASOs, or left untreated (no ASO). Protein extracts were collected at DIV21-Dpe15, digested with trypsin and analyzed by mass spectrometry. Unsupervised clustering revealed distinct proteomic signatures across conditions (Figure 2A). Among the 4,764 proteins identified, differential expression analysis (adjusted p value $\leq$0.05; |fold-change| $\geq$ 1.25) identified 62 proteins altered by PAN ASO treatment relative to controls, 606 proteins differing between PAN ASO- and SNP ASO-treated neurons, and 223 proteins differentially expressed in SNP ASOs-treated cultures compared with PAN ASO and control conditions (Figure 2B; Table S1). Among these proteins, 144 were unique to SNP ASO treatment, indicating a distinct molecular response to selective mut-HTT suppression.

Gene set enrichment analysis (GSEA) of SNP ASO-associated proteins highlighted enrichment in pathways linked to axonogenesis, synaptic vesicle cycling (as observed in the Gene Ontology [GO] biological processes), and mitochondrial function, including mitophagy and oxidative stress regulation (as observed in the Kyoto Encyclopedia of Genes and Genomes [KEGG] pathways a,d MSigDB Hallmarks) (Figure 2C).[37–39] Prominent differentially expressed proteins (DEPs) included (1) cytoskeletal motors (KIF5A/B/C, KIF1A/B, DYNC1H1) essential for axonal transport (Figure S4A); (2) metabolic enzymes (ACACA, LDHB, COX6C, and ATP5PB) involved in ATP production, several of which are downregulated in HD iPSCs[40] (Figure S4B); (3) synaptic proteins (DLG4/PSD-95, VAMP2, STX1B, SNCA, and NAPB) mediating vesicle fusion and neurotransmission (Figure S4C); and (4) Rab GTPases (RAB3B/C, RAB4A, RAB5A, and RAB18) regulating vesicular trafficking, previously shown to be disrupted in HD models[41–44] (Figure S4D).

Together, these findings show that allele-selective mut-HTT suppression by SNP ASOs induces a proteomic signature distinct from global-HTT loss, with changes in proteins associated with neuronal structure, synaptic integrity, and mitochondrial metabolism.

HTT-lowering selectivity of PAN- and SNP-ASO in a human corticocortical neuronal network-on-a-chip

To assess the impact of allele-selective HTT silencing on axonal transport of BDNF in human neuronal circuits, we reconstituted a corticocortical network using 109Q- and 69Q-iPSC-derived cortical neuronal precursors (Figure 3A). Neurons were cultured in microfluidic devices (MFDs) that compartmentalize presynaptic and postsynaptic populations connected via a central synaptic chamber, enabling the formation of polarized and functionally mature cortical networks.[45,46] Neuron precursors were seeded in pre- and postsynaptic chambers and differentiated for 21 days to yield dense, synaptically connected cortical networks. Immunostaining confirmed a high proportion of mature neurons ($MAP2^+$ 86.3%) with predominantly cortical ($TBR1^+$ 78.5%) and glutamatergic ($VGLUT2^+$ 78.1%) identities (Figure 3B). Comparable differentiation efficiency was observed in 69Q-derived networks (Figure S5A). These data confirm that HD-iPSC-derived neurons form physiologically relevant corticocortical circuits within the MFDs.

PAN, SNP, or NTC ASOs (4 μM) were added in all chambers of the MFDs at DIV1 and maintained in the culture medium without replenishment. Western blot analysis performed after 19–20 days confirmed robust and durable silencing of both HTT alleles by PAN ASO and selective suppression of mut-HTT by SNP ASO in both 109Q and 69Q neuronal networks (Figures 3C and 3D; Figures S5B and S5C). We next examined whether HTT silencing affected neuronal identity or differentiation. In 109Q networks, neither PAN nor SNP ASO altered the proportion of cortical ($TBR1^+$) or glutamatergic ($VGLUT2^+$) neurons, nor the overall $MAP2^+$ population (Figure 3E). Similar results were obtained in 69Q networks (Figure S5D). Together, these data demonstrate that SNP ASO achieves potent, selective, and sustained silencing of

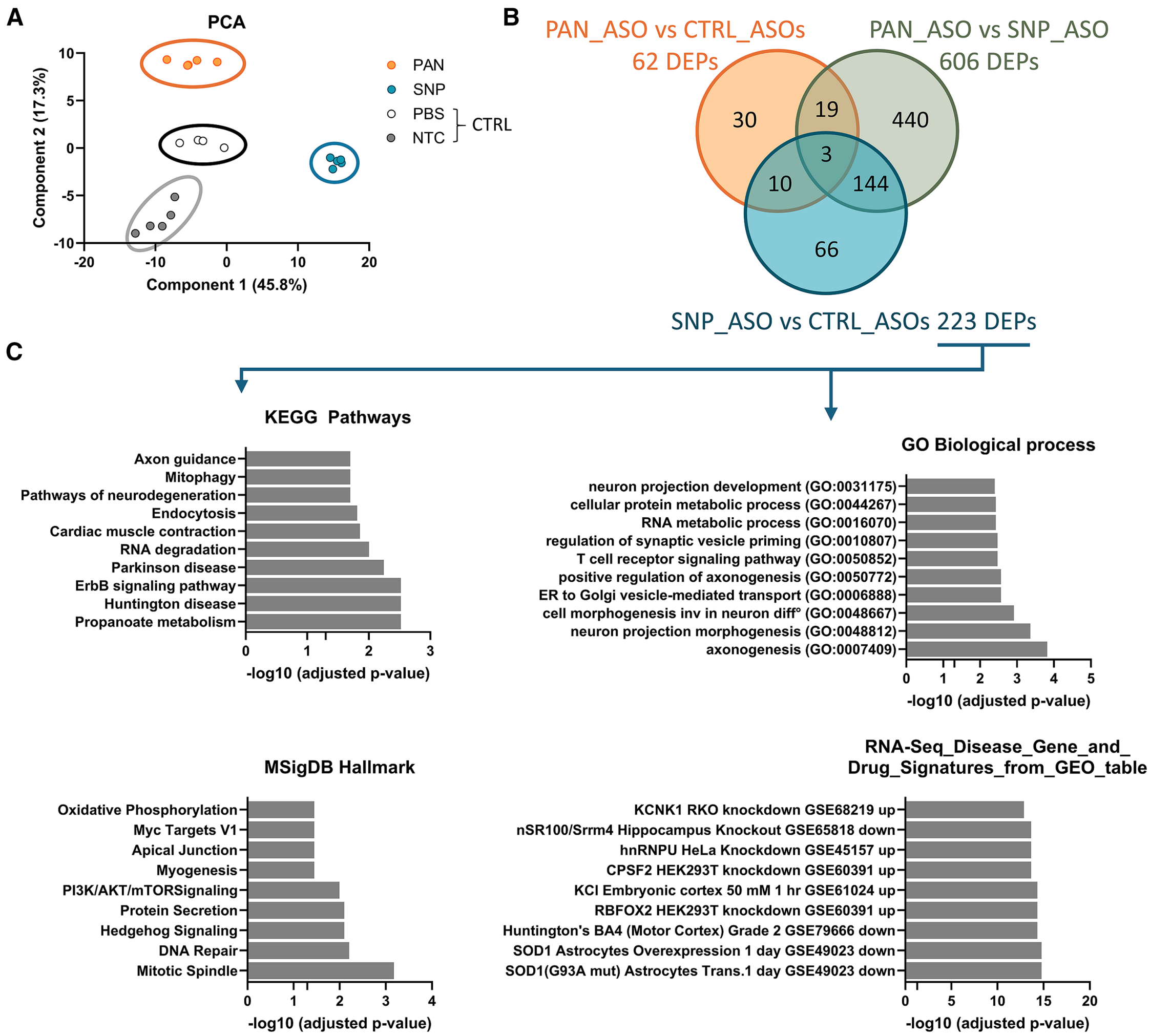

Figure 2. Proteomic signature of allele-selective versus pan-allelic HTT lowering in human HD cortical neurons

(A) Principal-component analysis of proteomic profiles from 109Q iPSC-derived cortical neurons exposed once at DIV6 to 4 μM SNP, PAN, or non-targeting control (NTC) ASO, or left untreated (no ASO). (B) Venn diagram of differentially expressed proteins (DEPs; adjusted *p* value ≤ 0.05; |fold-change| ≥ 1.25) showing overlaps between PAN-, SNP-, and control conditions (NTC & no ASO). Numbers indicate DEPs unique to each comparison or shared across treatments. (C) Gene set enrichment analysis (GSEA) of SNP-associated DEPs showing enrichment in pathways related to neuronal morphogenesis and axonogenesis, Huntington's disease, synaptic signaling and vesicle trafficking, and mitochondrial metabolism and ATP production.

mut-HTT in human corticocortical networks-on-chip without perturbing neuronal differentiation or subtype specification.

Allele-selective and PAN HTT silencing differentially affect axonal BDNF vesicle trafficking

To assess the functional consequences of allele-selective silencing on HTT-dependent transport, we monitored the axonal trafficking of BDNF in human HD neurons cultured in MFDs. Presynaptic neurons derived from 109Q were transduced at DIV0 with *BDNF-mCherry* lentivirus (Figure 4A). Presynaptic neurons derived from 69Q were transduced at DIV7 as they had the tendency to be more sensitive to transduction (Figure S6A). Cells were exposed at DIV1 to 4 μM PAN, SNP, or NTCs. Live imaging at DIV21-captured vesicle dynamics in distal axons confined within 500 μm microchannels (Figure 4B; Figure S6B). Kymograph-based analyses quantified segmental and global velocities, directionality, and the proportion of static vesicles (Figures 4C–4F; Figures S6C–S6F).

Silencing both wt-*HTT* and mut-*HTT* with PAN ASO reduced anterograde and retrograde velocities, decreased the number of anterograde vesicles, and lowered global transport speed relative to NTC ASO-treated neurons (Figures 4C–4E). In contrast, selective suppression of mut-HTT with SNP ASO preserved retrograde transport and global velocity while significantly reducing the proportion of static vesicles (Figures 4E and 4F). Mixed results were obtained in neurons derived from the 69Q-iPSC line (Figure S6). Despite their increased fragility

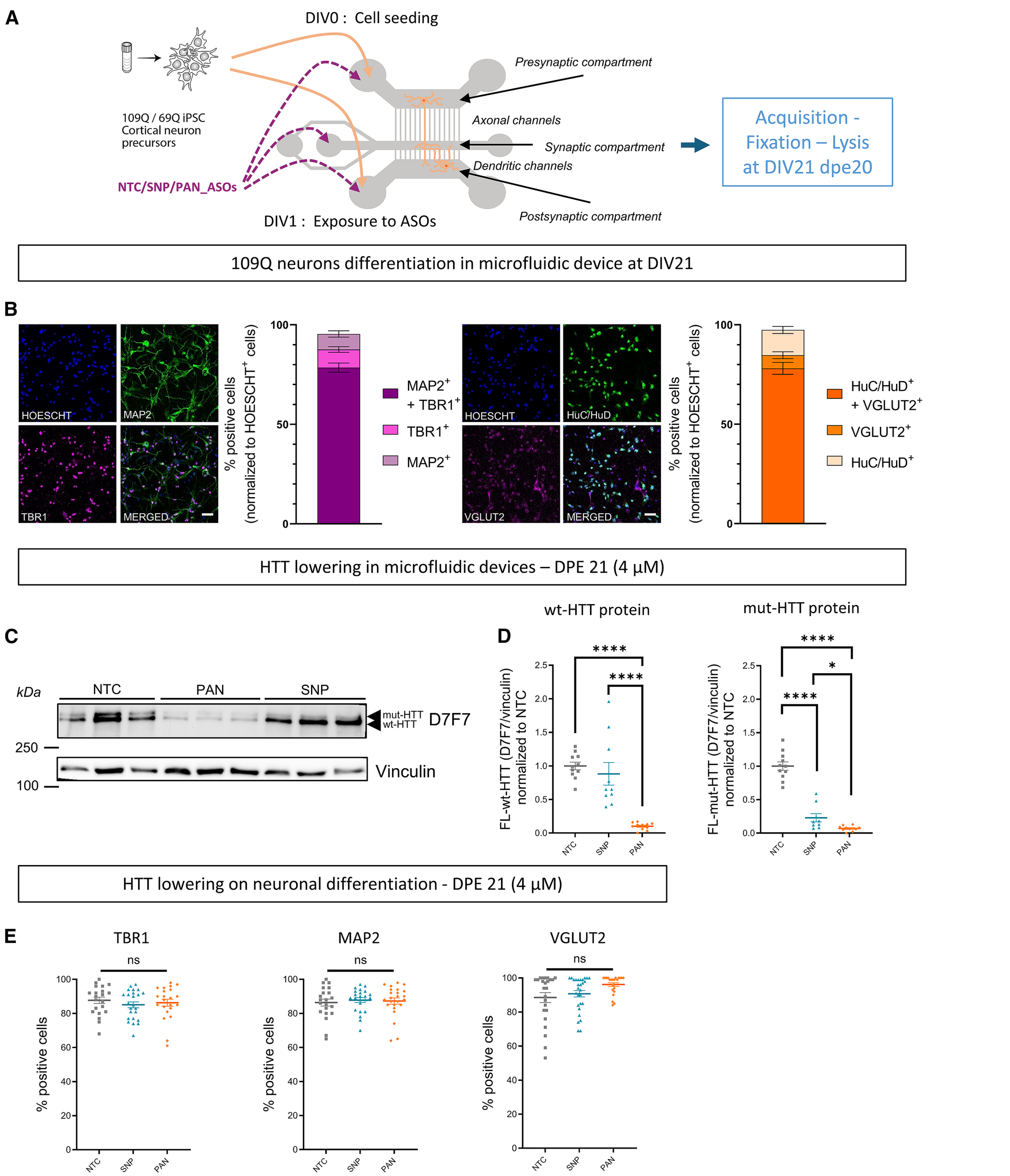

Figure 3. Allele-selective mutant HTT silencing in human corticocortical neuronal networks-on-chip

(A) Schematic of the microfluidic device (MFD) used to reconstitute human corticocortical networks, showing pre- and postsynaptic chambers connected by a central synaptic compartment. (B) Representative immunofluorescence images of 109Q iPSC-derived cortical neurons at DIV21, showing expression of neuronal and cortical markers HuC/D, MAP2, TBR1, and VGLUT2. (C and D) Western blot validation of *HTT* silencing following exposure at DIV1 to 4 μM NTC, PAN, or SNP ASOs for 20 days. Representative blots (C) and quantification of wt-HTT and mut-HTT (D) normalized to Vinculin. (E) Quantification of $TBR1^+$, $MAP2^+$, and $VGLUT2^+$ populations following treatment with NTC, PAN, or SNP ASOs. Data are mean ± SEM; *n* = 3–4 independent networks per condition; one-way ANOVA with Tukey's post hoc test; *p* values: **p* <0.05; ***p* = 0.01; ****p* <0.001; *****p* <0.0001. Scale bars, 50 μm.

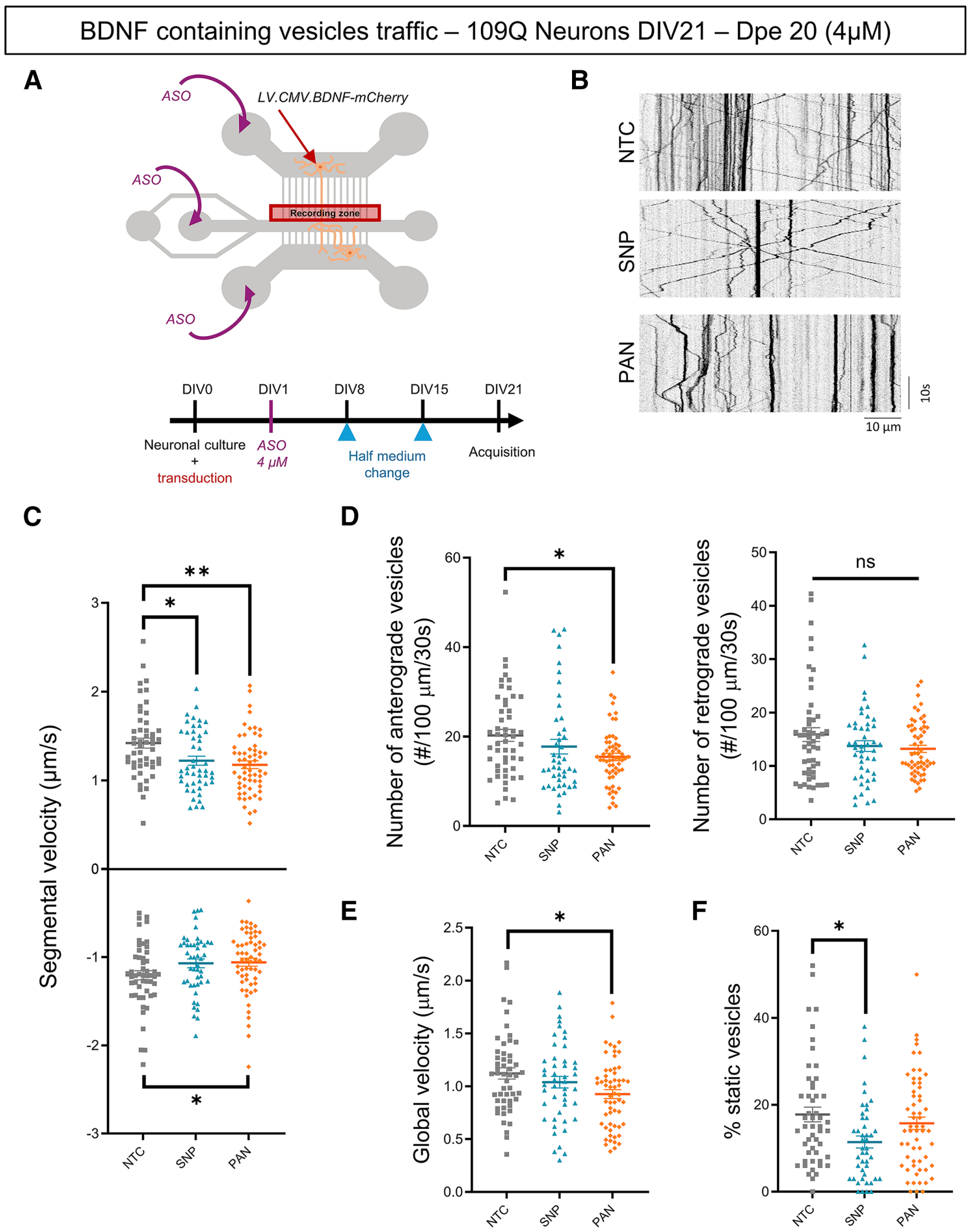

Figure 4. BDNF-mCherry vesicle transport following mutant selective or pan-allelic HTT silencing

(A) Schematic of the microfluidic device (MFD) used to reconstruct human corticocortical networks and experimental timeline. Presynaptic 109Q iPSC-derived cortical neurons were transduced at DIV0 with lentiviral BDNF-mCherry and treated at DIV1 with 4 μM NTC, PAN, or SNP ASOs. Live imaging of distal axons within 500-μm microchannels was performed at DIV21. (B) Representative kymographs showing BDNF-mCherry vesicle trajectories in neurons treated with NTC, PAN, or SNP ASOs. (C–F) Quantitative analyses of BDNF-mCherry transport. (C) Segmental velocities of anterograde and retrograde vesicles. Plotted on a single axis with anterograde velocities represented as positive values (upper portion of the graph) and retrograde velocities as negative values (lower portion of the graph). (D) Number of anterograde and retrograde vesicles per 100 μm of axon. (E) Global vesicle velocity. (F) Percentage of static vesicles.

Data represent mean ± SEM from three independent experiments (≥45 axons per condition). One-way ANOVA followed by Tukey's post hoc test (E, anterograde velocity in C) or Kruskal-Wallis test followed by Dunn's multiple comparison (C, retrograde velocity; D; F); *$p < 0.05$, **$p < 0.01$.

when plated into MFDs, 69Q neurons displayed similar impairments in anterograde BDNF transport but a lower number of static vesicles following PAN ASO treatment. In contrast, SNP ASO treatment, maintained vesicle dynamics at the level found in non-treated 69Q neurons. Together, these data indicate that selective mut-HTT suppression is associated with preservation of BDNF-mCherry vesicle motility relative to PAN HTT lowering, particularly in the 109Q neurons.

Allele-selective and PAN HTT silencing differentially affect neuronal homeostasis in human HD networks

We next compared the impact of PAN versus mutant-selective *HTT* silencing on neuronal homeostasis using human HD cortical neurons. Neuronal cultures were treated once at DIV1 with different concentrations (4, 8, or 16 μM) of PAN, SNP, or NTC ASOs, and survival, apoptosis, ATP content, and activity-dependent immediate-early gene induction were quantified up to DIV35 in the absence or presence of exogenous BDNF (Figures 5A–5G). Neuronal cultures were evaluated at DIV35 rather than DIV21, as this is the stage at which synaptic maturation becomes robust, evidenced by the appearance of fully synchronized calcium transients.[18]

PAN ASO suppression of both wt- and mut-HTT caused a dose-dependent reduction in MAP2+ neuron survival and a marked increase in cleaved caspase-3-positive cells compared to non-treated, NTC-treated or SNP-treated cultures without changing the proportion of MAP2+ cells or that of GABA+ neurons (Figures 5A and 5B; Figure S7). These effects were not rescued by BDNF supplementation, indicating intrinsic toxicity of global HTT loss (Figure 5D). In contrast, SNP ASO-treated neurons maintained viability relative to NTC-treated cultures. Consistent with these observations, PAN ASO significantly lowered intracellular ATP levels both with and without BDNF supplementation, whereas SNP ASO increased ATP content in a dose-dependent manner (Figures 5C, 5F, and 5G). Thus, while total HTT silencing impairs neuronal energetics and viability, selective suppression of mut-HTT enhances metabolic resilience.

To assess neuronal excitability, we quantified synapse density in 109Q networks grown in MFDs and treated with ASOs at DIV1. High-resolution Airyscan imaging of the presynaptic marker Bassoon and the postsynaptic scaffold protein Homer revealed

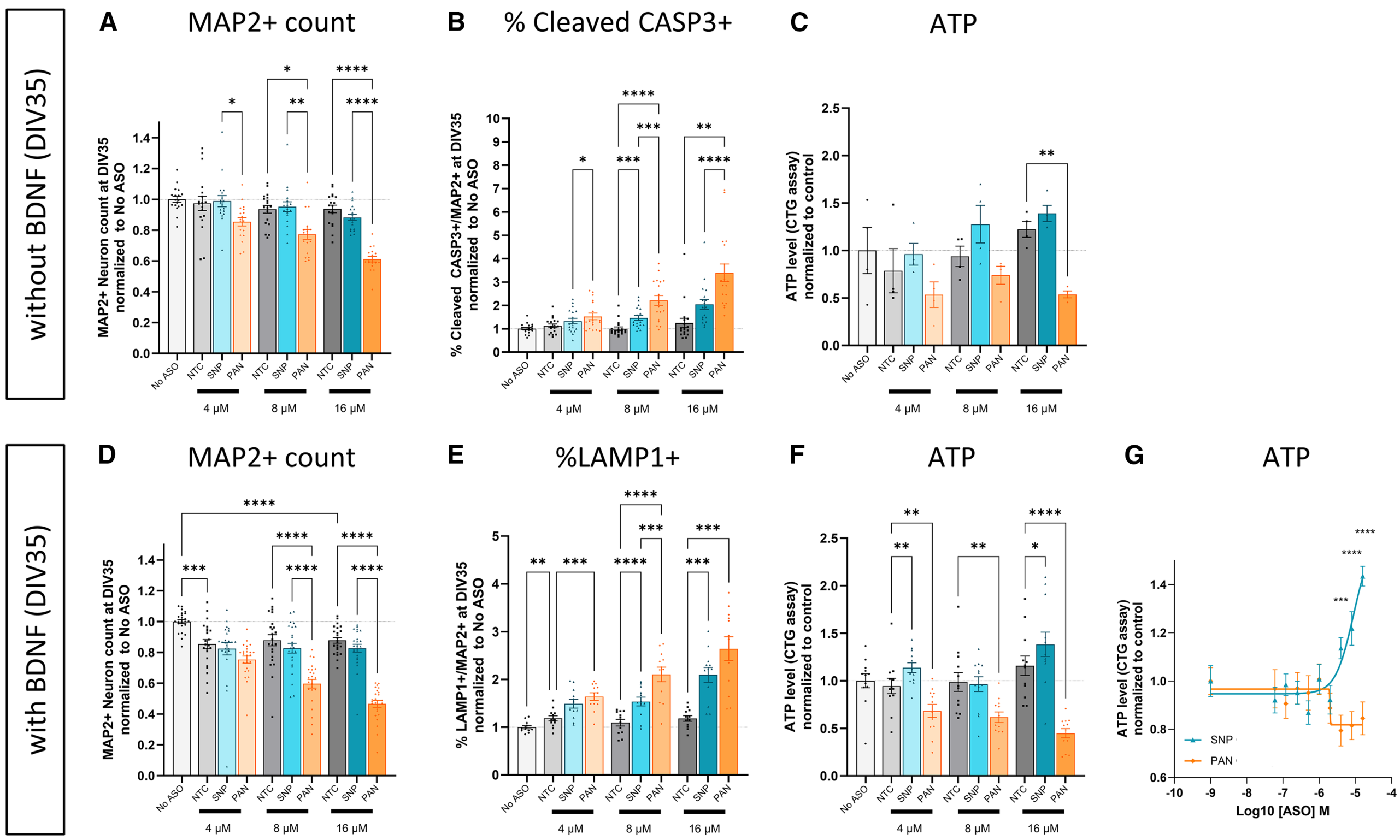

Figure 5. Effects of HTT silencing on neuronal survival, apoptosis, and cellular ATP levels

(A–G) Quantification of neuronal survival and apoptosis in 109Q cortical neurons exposed once at DIV1 to 0, 4, 8, or 16 µM non-targeting control (NTC, gray), allele-selective (SNP, teal), or pan-allelic (PAN, orange) ASOs. Cultures were maintained without (A–C) or with (D–G) BDNF supplementation from DIV14 onward. (A and D) MAP2$^+$ cells counts normalized to untreated control. (B) Percentage of cleaved Caspase-3$^+$ apoptotic neurons. (C, F, and G) Cellular ATP levels measured by CellTiter-Glo assay in neuronal cultures maintained (F and G) with or (C) without BDNF supplementation. (E) Percentage of LAMP1$^+$ neurons. (G) Dose-response analysis of cellular ATP levels following treatment with PAN or SNP ASOs.

Data represent mean ± SEM from ≥3 independent experiments (n = 6 cultures, 25 fields per condition). One-way ANOVA with Sidak's or Tukey's post hoc test; *$p < 0.05$, **$p < 0.01$, ***$p < 0.001$.

that total HTT silencing did not alter overall synapse density, whereas mut-HTT-selective silencing modestly increased the number of Homer-Bassoon colocalized puncta and significantly elevated Homer puncta alone (Figures 6A–6C).

We probed postsynaptic survival signaling after presynaptic activation in HD corticocortical networks in MFDs. At DIV21, glycine/strychnine stimulation of presynaptic neurons was followed by quantification of phosphorylated AKT (pAKT) and ERK (pERK) in the postsynaptic compartment (Figures 6D–6F; Figure S8). PAN ASO treatment markedly reduced the pAKT/AKT ratio, whereas SNP ASO preserved it at control levels (Figure 6F). Neither the post-synaptic glutamate receptor (GluA1) nor pERK/ERK ratios were statistically significantly changed by either ASO (Figure S8).[45]

To assess neuronal excitability, we measured expression of the immediate-early gene *cFOS* under basal conditions and after depolarization with KCl. Basal *cFOS* expression was elevated in both PAN and SNP ASO-treated groups relative to controls, with significantly higher levels in PAN-treated neurons at 16 µM (Figure 6G). Upon KCl stimulation, *cFOS* induction was most robust in SNP-treated neurons, particularly at 8 µM, while PAN ASO-treatment blunted this response, consistent with waning ASO efficacy over time (Figures 6H and 6I).

Together, these findings demonstrate that non-selective HTT silences compromises neuronal survival, energy metabolism, and activity-dependent signaling, whereas allele-selective suppression of mut-HTT preserves metabolic and functional homeostasis.

Allele-selective and PAN HTT silencing differentially affect synaptic connectivity and network activity in human HD neuronal networks

Wild-type HTT is essential for synaptic development and network maturation, and its depletion impairs neuronal connectivity.[17,18] We therefore assessed whether non-selective *HTT* silencing by PAN ASO similarly disrupts synaptic function in HD human neurons, and whether mutant-selective SNP ASO exerts more limited effects.

Synchronized neuronal activity was quantified using the calcium indicator Cal-520 at DIV28 and DIV35 following a single or weekly

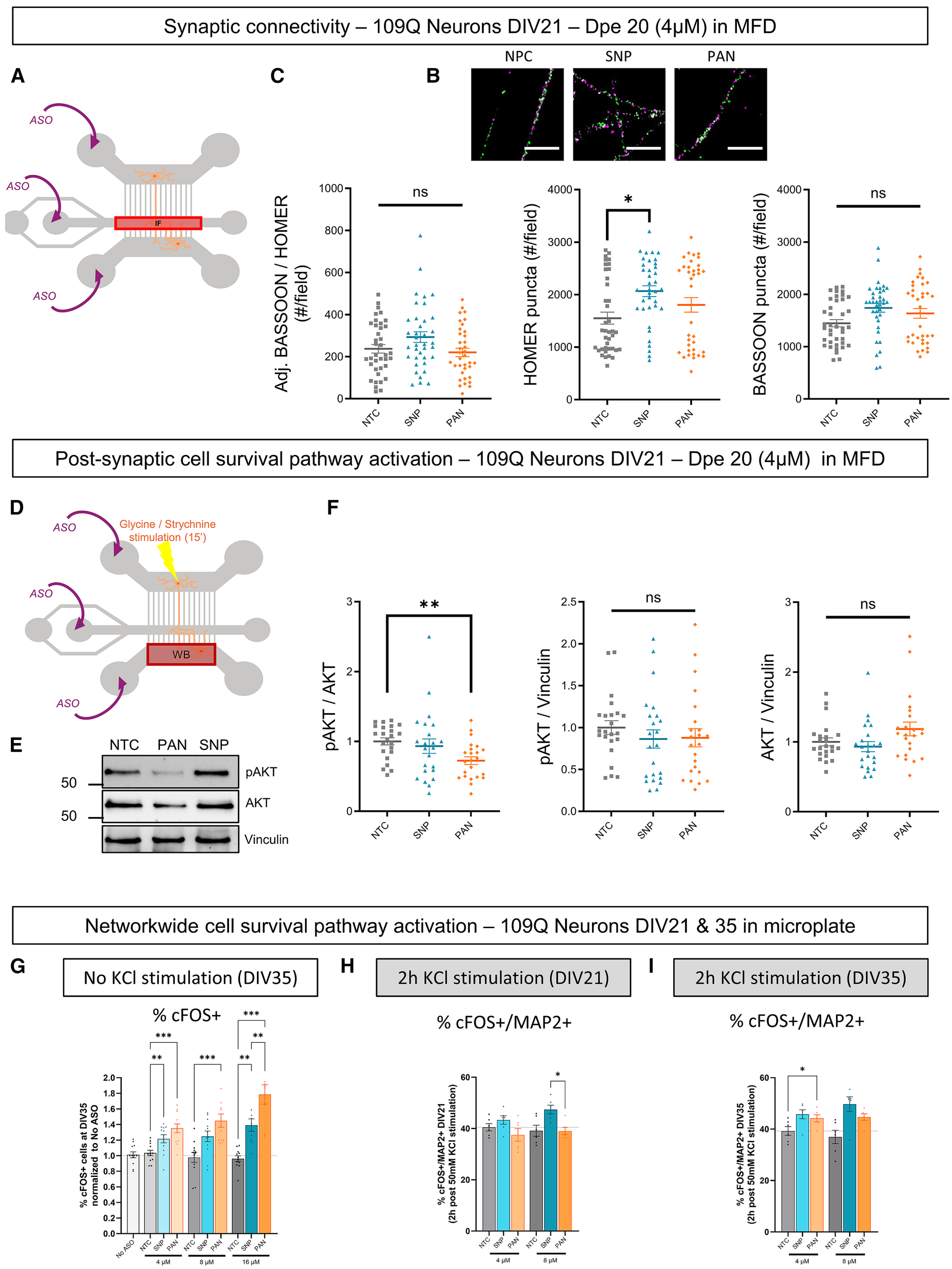

Figure 6. Synaptic markers and activity-dependent signaling following mutant mutant selective pan-allelic HTT silencing
(A) Schematic of microfluidic device (MFD) used to reconstruct human corticocortical networks, indicating ASO treatment (NTC, in gray, SNP in teal, or PAN in orange) and synaptic recording zone. (B) Representative Airyscan images showing immunofluorescence for the postsynaptic marker Homer (green) and presynaptic marker Bassoon (magenta) in the synaptic chamber. (C) Quantification of Homer-Bassoon colocalized synapses and total Homer and Bassoon puncta. (D) Schematic of the microfluidic

(legend continued on next page)

ASO exposure from DIV1. Single exposure to PAN ASO induced a dose-dependent reduction in both the frequency and amplitude (AMP) of spontaneous calcium oscillations, culminating in the near-complete loss of network-wide synchronization at concentrations ≥8 μM (Figures 7A–7H). In contrast, SNP ASO produced significantly more moderate decreases in oscillatory activity. Comparable results were obtained in both 109Q- and 69Q-derived cortical cultures subjected to repeated ASO exposure (DIV1, 14, 28) (Figure S9). At DIV35, when SNP ASO efficacy had partially waned, calcium oscillations remained significantly more preserved in SNP- than PAN-treated networks (Figure S10).

Together, these findings indicate that non-selective HTT suppression disrupts network synchronization and activity-dependent connectivity, while selective mut-*HTT* silencing is associated with preservation of these measures relative to PAN HTT lowering.

DISCUSSION

Lowering mut-HTT remains a leading therapeutic strategy for HD, as recent advances have established its efficacy in targeting the central driver of disease pathogenesis.[47] Nevertheless, non-selective suppression approaches require careful consideration: given the indispensable cellular roles of wild-type HTT, lowering wild-type HTT with a pan-silencing approach could compromise neuronal integrity and viability.[7] To address these concerns, we used patient iPSC-derived cortical neurons to directly compare PAN and allele-selective *HTT* silencing via ASOs. Our findings show that, in human HD neurons, PAN HTT lowering is associated with impairments in neuronal homeostasis, metabolic function, and synaptic network connectivity, whereas allele-selective mut-HTT suppression, which spares substantially more wt-HTT, is associated with preservation of these functions. Selective mut-HTT silencing was associated with preservation of vesicle transport and synaptic measures relative to PAN HTT lowering. While the overall effects of allele-selective versus PAN HTT lowering were consistent across the 69Q and 109Q models, some effects, particularly those on BDNF-mCherry vesicle trafficking, were more pronounced and consistent in 109Q neurons. These observations are in line with our previous studies demonstrating detrimental effects of substantial wt-HTT depletion in human neuronal models,[18,34] although the present study do not formally establish that the phenotypes induced by PAN ASO are caused specifically by the loss of wt-HTT since the changes in HTT overall content are very different between PAN and SNP conditions. Bragg et al. recently reported greater molecular benefit of mutant-selective than PAN HTT lowering in Q111 mice49. However, their mutant-selective ASO achieved greater mHTT lowering and reduced the toxic HTT1a species, indicating that it is uncertain whether the improved outcomes can be attributed solely to preservation of wt-HTT. Bragg et al. recently reported greater molecular benefit with an allele-selective ASO that achieved both greater mHTT lowering and reduction of the toxic HTT1a species compared with a pan-lowering ASO,[29] making it uncertain whether the improved outcomes can be attributed solely to preservation of wt-HTT.

A limitation of the present study is that healthy WT neurons were not included as an experimental comparator. Thus, the present data do not establish that allele-selective HTT lowering restores HD-associated phenotypes to the state of untreated healthy neurons. Rather, our findings indicate that, within HD neuronal cultures, selective mut-HTT lowering is associated with better preservation of neuronal functions than PAN HTT lowering. This interpretation is consistent with our previous studies in hiPSC-derived neurons showing that substantial HTT depletion, including genetic HTT inactivation and RNA-mediated HTT lowering, can impair neuronal maturation and network function.[18,34]

Allele-selective ASOs achieve potent and durable mut-HTT suppression

A single gymnotic exposure to stereopure SNP-targeting ASO provided robust (>80%–90%), durable (1 month), and highly selective reduction of mut-HTT mRNA and protein, with minimal impact on wt-HTT. These results are consistent with previous reports highlighting improvements in ASO stability and intracellular persistence with stereopure backbone chemistry.[27,35,48] The substantial allele discrimination achieved in human neurons *in vitro* supports the clinical feasibility of SNP-targeted HTT-lowering strategies (e.g., WVE-003, NCT05032196, which was the first demonstration of allele-selective lowering in a clinical trial; RG6496, NCT07246941, which has recently initiated first-in-human dosing as of this publication), suggesting infrequent dosing may sustain therapeutic benefit while minimizing off-target effects.[27] However, such strategies remain complex, requiring appropriate dosing and, be limited to patients responsive to the SNP-specific ASOs. As such, Roche has recently stopped the development of RG6496, an allele-selective ASO targeting a mut-HTT-linked SNP (POINT-HD trial (NCT07246941)).

Mut-HTT selective depletion alters pathways compromised in HD

Quantitative proteomics revealed that non-selective *HTT* silencing and allele-selective silencing caused significant yet distinct changes

device (MFD) used to reconstitute human corticocortical networks, showing the postsynaptic protein extraction zone at DIV21 following 15 min of presynaptic stimulation with glycine/strychnine solution. Neurons derived from 109Q iPSCs were treated at DIV1 with 4 μM NTC (Gray), SNP (teal), or PAN (orange) ASOs. (E and F) Western blot analysis of pAKT and total AKT expression, normalized to Vinculin, in 109Q neuronal networks treated with NTC, SNP, or PAN ASOs. (G–I) Quantification of cFOS expression under basal conditions (G) and after 50 mM KCl stimulation (H and I) at DIV21 or DIV35.

Data represent mean ± SEM. For microplate cultures, data are from ≥3 independent experiments (*n* = 6 cultures, 25 fields per condition). One-way ANOVA with Sidak's or Tukey's post hoc test; **p* < 0.05, ***p* < 0.01, ****p* < 0.001. For MFD experiments, *n* = 36 fields per condition for (A–C); Kruskal-Wallis test with Dunn's multiple comparisons; ns, not significant; **p* < 0.05, ***p* < 0.01. For (D–F), four independent experiments (*n* = 12–16, MFDs per condition); one-way ANOVA with Tukey's post hoc test; **p* < 0.05, ***p* < 0.01 for (D–F). Scale bars, 5 μm.

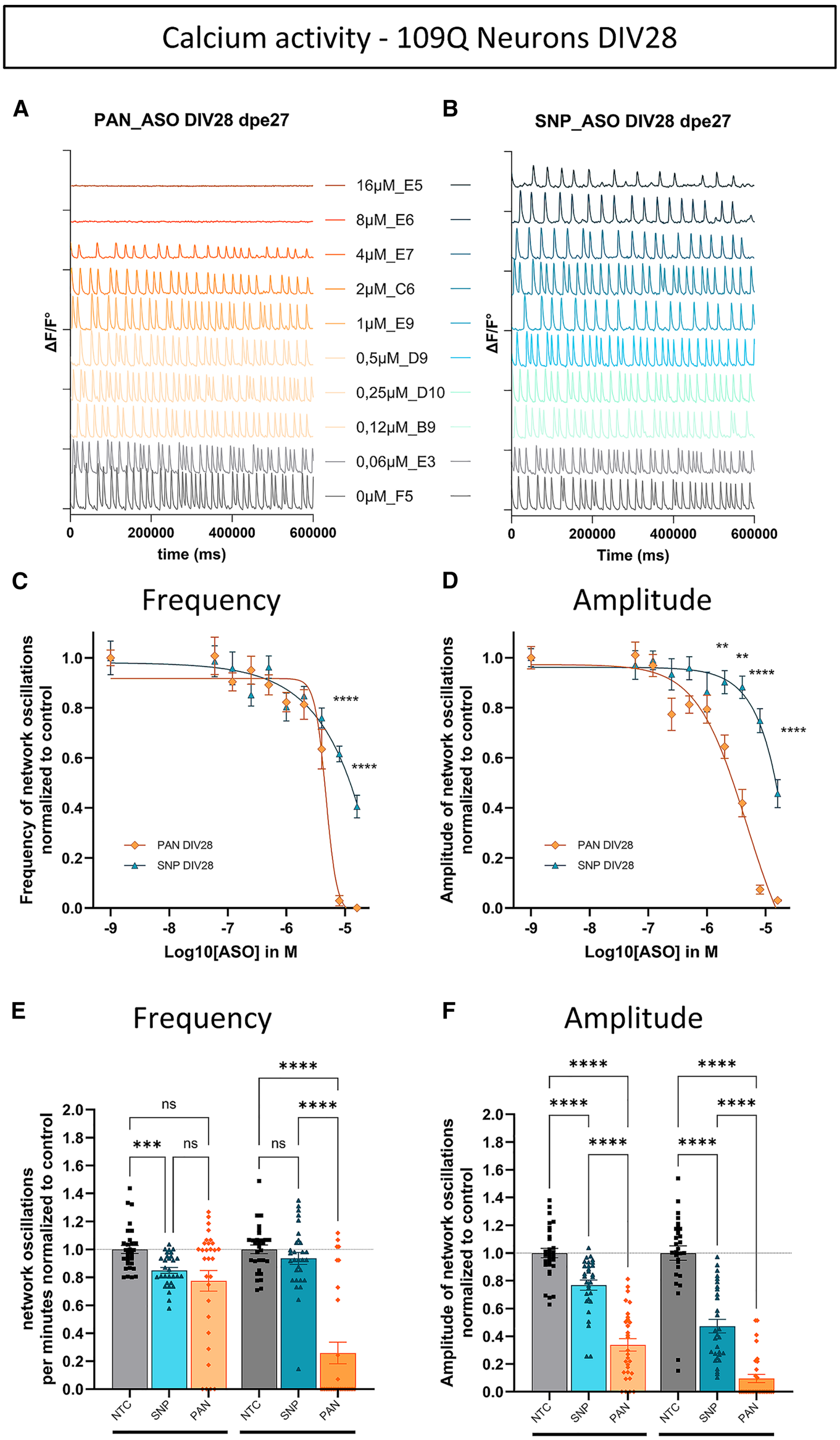

Figure 7. Synaptic network activity following allele-selective or pan-allelic HTT silencing

(A–H) Quantification of spontaneous calcium oscillations in cortical networks derived from 109Q human iPSCs following a single exposure at DIV1 to 0–16 μM non-targeting control (NTC, gray), allele-selective (SNP, teal), or pan-allelic (PAN, orange) ASOs. Network activity was assessed at DIV28 by whole-well Cal-520 fluorescence recording using a kinetic plate reader. (A and B) Representative 10 min traces showing synchronized calcium transients across conditions. (C and D) Dose-response analysis of normalized oscillation frequency and amplitude. (G and H) Quantification of mean frequency (G) and amplitude (H) after exposure to 4 or 8 μM ASOs.

Data represent mean ± SEM from three independent experiments (six wells per condition); one-way ANOVA with Sidak's multiple comparisons; $^{**}p < 0.01$, $^{***}p < 0.001$, $^{****}p < 0.0001$.

in the abundance of proteins. Specifically, allele-selective silencing modulated a narrower set of proteins (223), which are frequently pivotal to the scaffolding functions of wt-HTT and/or are compromised in HD or in neurodegeneration (KEGG Huntington disease-hsa05016, pathways of neurodegeneration-hsa05022 and Parkinson disease-hsa05012). The proteins in question were enriched in a variety of biological processes, including axonogenesis (GO:0007409), axon guidance (hsa04360), and neuron morphogenesis (GO:0048812). These processes have been found to be compromised by mut-HTT in various models.[32,49–52] In addition, these proteins have been implicated in vesicle transport, protein secretion, and endocytosis, which have also been shown to be altered in HD models.[12,43,44,53] Furthermore, these 223 proteins are enriched in proteins involved in mitochondrial function and metabolism (mitophagy, oxidative phosphorylation) also altered in HD.[40,54,55] Finally, these proteins have been associated with synaptic function, such as the regulation of synaptic vesicle priming (GO0010807), or mirrors the signature of a compensatory responses to KCNK1 loss in R6/2 mice. Comparison with the 606 proteins differing between PAN- and SNP-treated neurons revealed partial overlap in enriched pathways, with approximately half of the top SNP ASO-associated KEGG pathways and a subset of the Hallmark and GO biological process terms also significantly enriched in this comparison. Thus, PAN and SNP ASO treatments induce partly overlapping but distinct proteomic responses. However, because the ASOs differ in target sequence, allele selectivity, and extent of HTT lowering, these differences cannot be unequivocally attributed to the HTT-lowering strategy, and some may be ASO-specific.

Consistent with these changes, global-HTT suppression led to a reduction in ATP levels, an enhancement in caspase-3 cleavage, and a decline in $MAP2^+$ neuron survival. These effects were not rescued by exogenous BDNF, indicating that wt-HTT loss impairs intrinsic metabolic programs. Conversely, selective mut-HTT silencing was associated with higher ATP content and maintained viability relative to PAN HTT lowering. These findings are consistent with preservation of cellular metabolic function under conditions in which a more extensive HTT depletion including the WT allele produces metabolic and viability deficits. Collectively, these results substantiate the notion that wt-HTT plays a pivotal role in preserving neuronal proteostasis and bioenergetic capacity. Importantly, in the absence of a healthy WT proteomic reference, the SNP-associated proteomic profile cannot be fully interpreted as a restoration toward a normal neuronal proteomic state, but rather as a response distinct from that induced by PAN HTT lowering.

Wild-type HTT is critical for synaptic integrity and network activity

Calcium imaging revealed that global *HTT* silencing severely disrupted network synchronization, causing a dose-dependent reduction in oscillatory frequency and amplitude, and a near-complete loss of coordinated activity. These functional deficits were paralleled by impaired AKT activation and altered expression of synaptic proteins, consistent with the established role of wt-HTT in synapse assembly and plasticity.[17,18] By contrast, SNP ASO produced only modest decreases in activity and even increased the density of $Homer^+$ synapses, consistent with relative preservation of synaptic connectivity following selective mut-HTT suppression. These results highlight the importance of preserving wt-HTT to sustain network homeostasis and underscore that excessive total HTT reduction, as achieved in halted tominersen trials,[56,57] may underlie clinical worsening.

Selective mut-HTT suppression ameliorates axonal transport and preserves function

Selective silencing of mut-*HTT* was associated with the preservation of BDNF-mCherry vesicle motility, reduced vesicle stalling, and maintained balanced anterograde and retrograde transport relative to PAN HTT lowering. Because mut-HTT has been implicated in altered with motor protein engagement and microtubule dynamics,[5,58,59] its selective removal likely re-establishes normal cargo trafficking, promoting efficient BDNF delivery to synaptic terminals. These effects were observed together with the preservation of calcium network activity and synaptic structure observed after SNP ASO treatment. However, the present experiments do not establish restoration to a healthy neuronal state.

Therapeutic implications

Our data provide mechanistic evidence supporting the rationale for preserving wt-HTT during therapeutic HTT lowering in HD. The present findings suggest that more extensive depletion of total HTT can be associated with adverse effects on neuronal functions, whereas selective mut-HTT lowering, which preserves substantially more total HTT, most of it being WT HTT, is associated with preservation of these functions. Consistent with this interpretation, previous animal and human studies have shown that substantial or transient HTT depletion can disrupt neuronal functions, highlighting the importance of carefully controlling the extent of HTT lowering during therapeutic development.[60,61] The SNP ASO used in this study and the clinical candidate WVE-003, both target SNP3 (rs362273). Based on literature reports, we expect up to 40% of the population of individuals with HD would be amenable to a SNP3-targeting ASO.[62–64] The results of the present study are broadly consistent with the publicly-reported findings of the SELECT-HD trial (NCT05032196), in which WVE-003 lowered mut-HTT while preserving wt-HTT. Notably, mut-HTT-lowering was associated with slowing of caudate atrophy. Subsequent research endeavors should concentrate on optimizing the administration of ASO, with a particular focus on the dosing and timing of the intervention, with the objective of achieving maximal mitigation of mut-HTT. Our human corticocortical networks in microplate format and in MFD, which recapitulate long-range connectivity and functional maturation, offer a scalable preclinical platform to define these boundaries.

Conclusions

Together, these data support the notion that wt-HTT is indispensable for neuronal metabolism, transport, and network synchronization in human HD neurons. Non-selective silencing has been shown to undermine these processes, leading to energetic collapse and synaptic instability. In contrast, allele-selective strategies were associated with preservation of cellular and network functions relative to more extensive HTT depletion. These mechanistic insights support the rationale for SNP-based, mutant-selective HTT-lowering therapies, emphasizing the need to maintain physiological wt-HTT expression for safe and durable clinical benefit.

MATERIALS AND METHODS

Oligonucleotides

The SNP ASO used in this study has been described previously as "HTT-1533"[27] and targets SNP3 (rs362273). The PAN ASO has been described previously as "MutASO."[29]

hiPSC cultures

The HD-hiPSC "109Q" line (repository lD ND42222, XX, 109 CAG repeats, passage 42) was obtained from Coriell Institute repository. The HD-hiPSC "69Q" line (GM21756, 69 CAG repeats, clone 1 passage 10) was produced for Wave Life Sciences by iXCells Biotechnologies from HD patient fibroblasts obtained from the NIGMS Human Genetic Cell Repository (repository lD GM21756). HD-iPSCs were maintained on vitronectin-coated (Life Technologies) plates in mTeSR Plus medium (STEMCELL Technologies). Cultures were fed every other day and passaged via manual dissociation using 0.02% EDTA (0.25 mM; pH 7.2; Sigma-Aldrich) every four to five days.

Cortical differentiation of hiPSCs

hiPSC colonies were treated (day *in vitro* 0; DIV0) as previously described[65] in N2B27 medium consisting of 50% Gibco DMEM/F-12 (GlutaMAX supplement, Life Technologies) and 50% Neurobasal

Medium (Life Technologies), 1% Gibco *N*-2 Supplement (Life Technologies), 2% Gibco B-27 Supplement without vitamin A (Life Technologies), and 50 μM of 2-Mercaptoethanol (Life Technologies). Neural differentiation was initiated, passaging the hiPSCs in N2B27 medium supplemented with SB431542 (20 μM; Tocris Bioscience), LDN-193189 (100 nM; Sigma-Aldrich), XAV-939 (1 μM; Tocris Bioscience), and Y-27632 Dihydrochloride (10 μM; STEMCELL Technologies) in low-adherence culture plates (Greiner Bio-one) for 6 h. Then hiPSC aggregates were transferred on poly-L-ornithine (PO; Sigma-Aldrich) and laminin (Life Technologies)-coated dishes. For that, plates were coated with 5.1 μg/cm^2 PO for 6 h at 37°C. Afterward, culture plates were washed three times with phosphate-buffered saline (PBS, Sigma-Aldrich) and subsequently coated with 0.72 μg/cm^2 of laminin overnight at 37°C. Media were changed every day from DIV0 to DIV20. At DIV1, Y-27632 Dihydrochloride was removed. From DIV5 to DIV9, SB431542 was removed and human fibroblast growth factor (FGF)-basic (10 ng/mL; PeproTech) and cyclopamine (1 μM; Sigma-Aldrich) were added. From DIV10 to DIV17, LDN-193189 and XAV-939 were removed and CHIR99021 (0.4 μM; Ozyme) was added. At DIV17, cortical neuron precursors were enzymatically dissociated using accutase (Life Technologies), resuspended at 5 × 10^6 cells/mL in Cryostor (Sigma-Aldrich) cell cryopreservation medium, frozen, and stored in liquid nitrogen vapor at −150°C. Shipments of cortical progenitors to F. Saudou lab were conducted on dry ice.

Neuronal maturation of cortical progenitors

Cortical neuron precursors were resuspended in N2B27 medium, supplemented with BDNF (20 ng/mL; PeproTech), dbcAMP (100 μM; Sigma-Aldrich), DAPT (10 μM; Tocris Bioscience), and Y-27632 Dihydrochloride (10 μM; STEMCELL Technologies). Subsequently, cells were plated on poly-L-ornithine (PO, Sigma-Aldrich) and laminin (Life technologies)-coated dishes, achieving a final density of approximately 100,000 to 150,000 cells/cm^2. Multi-well culture plates (6-well plate Corning Costar, 12-well plate Corning Costar, or 96-well plate Corning CellBIND) were coated with 5.1 μg/cm^2 PO for 6 h at 37 °C. Afterward, culture plates were washed three times with phosphate-buffered saline (PBS, Sigma-Aldrich) and subsequently coated with 0.72 μg/cm^2 of laminin overnight at 37 °C. At day *in vitro* 1 (DIV1), the entire medium was replaced with N2B27 medium supplemented with BDNF (20 ng/mL), dbcAMP (100 μM), DAPT (10 μM), and PD0332991 (2.5–5 μM, Tocris Bioscience). At DIV7, the medium underwent half of the medium replacement with BrainPhys neuronal medium N2-A & SM1 kit (BrainPhys medium; STEMCELL Technologies), supplemented with BDNF (20 ng/mL), dbcAMP (100 μM), DAPT (5 μM), and PD0332991 (1–2.5 μM). At DIV14, half of the medium was again replaced with BrainPhys medium, supplemented with BDNF (20 ng/mL), dbcAMP (100 μM), DAPT (1 μM), and PD0332991 (0.5 μM). Medium was then completely replaced every week from DIV21 with BrainPhys medium, supplemented with BDNF (20 ng/mL), dbcAMP (100 μM) and PD0332991 (0.5–1.43 μM). If applicable, ASOs diluted in PBS were added gymnotically at DIV1 or 7, or DIV1 and 14 and 28, depending on the experiments.

ASO delivery

ASOs were received from WAVE Life Sciences in a lyophilized form and stored at −80 °C. ASOs were resuspended in PBS at 2 mM and stored at −20 °C. Neurons were exposed to different concentrations of ASOs, diluted in the culture medium at DIV1, 7, 14, and/or 28.

Neuronal maturation of cortical progenitors in MFDs

MFDs were generated as previously described.[66] Devices were coated with poly-D-lysine (5MG, Merck) in the proximal and synaptic compartment and poly-D-lysine/laminin (MERK) in the distal compartment. hiPSCs-derived cortical neuron precursors were plated in N2B27 at a final density of ∼10,000 cell/mm^2 in the proximal and the distal compartments of the microfluidic device. For BDNF trafficking experiment, 109Q cells were infected at DIV0 with lentivirus CMV.*BDNF-mCherry*[66] in the proximal chamber. The 69Q cells were infected later at DIV7 with the same lentivirus as they had the tendency to be more sensitive to transduction compared to the 109Q cells. The day after plating, we replaced the medium with fresh N2B27-supplemented medium. Similar medium composition was used than described in the neuronal maturation section. For 69Q neurons transduced with *BDNF-mCherry* lentivirus at DIV6, medium was changed at DIV7 and replaced with fresh supplemented medium containing ASOs at 4 μM.

Immunofluorescence analysis

Neuronal cultures were fixed using a solution containing 4% paraformaldehyde (PFA, Electron Microscopy Sciences) and 4% sucrose (Sigma-Aldrich) diluted in PBS for 10 min at room temperature (RT). Following fixation, cells underwent three washes with PBS and were then incubated overnight at 4°C with primary antibodies. The primary antibodies were diluted in PBS supplemented with 2% bovine serum albumin (BSA, Sigma-Aldrich) and 0.2% Triton X-100 (Sigma-Aldrich). The specific antibodies used are TBR1 (1:500, rabbit, Abcam), MAP2 (1:3,000, chicken, BioLegend), HuC/HuD (1:500, mouse, Life Technologies), GABA (1:4,000, rabbit, Sigma-Aldrich), cFos (2H2, 1:500, mouse, Abcam), EGR1 (15F7, 1:500, rabbit, Cell Signaling Technology), GFAP (1:1,000, Millipore), and VGLUT2 (1:500, Synaptic System). Following this incubation, cells were washed three times with PBS and then incubated for 1 h at RT with DAPI Dihydrochloride (1:1,000, Calbiochem) and combinations of secondary antibodies. The secondary antibodies were diluted in PBS supplemented with 2% BSA and 0.2% Triton X-100 and included Alexa Fluor 488 donkey anti-mouse IgG (1:1,000, Life Technologies), Alexa Fluor Plus 555 donkey anti-rabbit IgG (1:1,000, Life Technologies), and Alexa Fluor 647 donkey anti-chicken IgG (1:1,000, Jackson ImmunoResearch). Following another three washes with PBS, images were captured using the HCS CellInsight CX7 Platform (Life Technologies) and subsequently processed using ImageJ software.

For experimentation in MFDs, 69Q or 109Q human neurons were respectively fixed at DIV20 or DIV21 with a PFA/sucrose solution (4%/4% in PBSEuromedex) for 20 min at RT and then washed three times with PBS to remove the buffer. Neurons were permeabilized

with 0.3% Triton X-100 (Merck) in PBS for 10 min at RT. Neurons were then incubated for 1 h at RT with a blocking solution (1% BSA [Euromedex], 2% normal goat serum (Ab7481, Abcam). The compartment of interest was then incubated with primary antibodies against MAP2 (1:500, ab5392, Abcam), TRB1 (1:500, ab31940, Abcam), HuC/HuD (1:250, A-21271, Invitrogen), VGlut2 (1:500, 135403, Synaptic System), HOMER (1:500, 160006, Synaptic System) or BASSOON (1:500, ab82958, Abcam) overnight at 4°C. MFDs were washed three times with PBS for 10 min before incubation with appropriate fluorescent secondary antibodies for 1 h at RT. Then, secondary antibodies were washed as described previously for primary antibodies, and a dye (Hoescht, Invitrogen, 1:5,000) was added for 10 min at RT, then washed with PBS for nuclear staining.

Presynaptic neuron stimulation in microfluidic chambers

To study cell survival pathways activation in postsynaptic neurons, presynaptic neurons were stimulated at DIV21 for 15 min at 37°C with a 1× glycine/strychnine solution (dilution of 10× solution: 25 mM HEPES pH 7.5 [Euromedex], 124 mM NaCl [Merck], 3 mM KCl [Merck], 2 mM CaCl2 + 2H2O [Merck], 10 mM glucose [Merck], 200 μM glycine [Euromedex], and 1 μM strychnine [Merck] in sterile water). After stimulation, postsynaptic neurons were lysed for protein quantification as described subsequently.

Western blotting analysis

Protein samples were extracted from cortical neuron cultures using a RIPA lysis solution (Sigma-Aldrich) at 8 μL/cm^2 or 15 μL per well of 96-well plate, supplemented with phosphatase inhibitor cocktail tablet (Roche) and protease inhibitor cocktail (Sigma-Aldrich). Total protein quantification was performed (except for 96-well plate extracts) with the Pierce BCA protein assay kit (Thermo Fisher Scientific) following the manufacturer's instructions. Samples were diluted in ultra-pure H2O, sodium dodecyl sulfate (SDS) XT Sample buffer (4X, Bio-Rad Laboratories), and DL Dithiothreitol solution (DTT 20X, Sigma-Aldrich), then denatured at 70°C (or 90° for 96-well plate extracts) for 10 min.

For gel electrophoresis, 4 μg of proteins (or 14 of 15 μL of 96-well plate extracts) or a molecular weight marker (HiMark pre-stained HMW protein standard, Thermo Fisher Scientific) were loaded into a 4%–20% Criterion TGX precast polyacrylamide protein gel (Bio-Rad Laboratories). SDS-PAGE was performed in Tris-Glycine-SDS buffer (Bio-Rad Laboratories) for 2 h at 50 V and 3 h at 100 V. The proteins were transferred with iBlot2 NC Regular Stacks (Thermo Fisher Scientific) for 7 min at 20 V onto nitrocellulose membranes.

The membranes were incubated for 1 h at RT under agitation in a blocking buffer composed of PBS-BSA 5% (PBS supplemented with 5% BSA), followed by an overnight incubation at 4°C with primary antibodies against Huntingtin D7F7 (1:1,000, rabbit, Cell Signaling Technology) and Huntingtin poly-glutamines (1:1,000, mouse, Sigma-Aldrich) diluted in PBS-T-BSA [PBS-BSA 5% supplemented with 0.1% Tween 20 (Sigma-Aldrich)]. After three washes with PBS-T [PBS supplemented with 0.1% Tween 20] for 10 min each, the membranes were incubated with secondary antibodies diluted in PBS-T-BSA: donkey anti-rabbit 800CW IRDye (1:15,000, Li-COR Biosciences) and donkey anti-mouse 680RD IRDye (1:15,000, Li-COR Biosciences) for 1 h at RT. Three additional washes in PBS-T were performed prior to quantification of antibody binding using a Li-Cor Odyssey CLx imager and Image Studio Lite 5.2 software. For protein extracts from 96-well plates, antibody binding was quantified using an Invitrogen iBright FL1500 Imaging System (Thermo Fisher Scientific) and Fiji/ImageJ software.

For reference protein quantification, the same membranes were reused. They were incubated overnight at 4°C with an antibody directed against Calnexin (1:2,500, rabbit, Sigma-Aldrich) diluted in PBS-T-BSA. Following three washes, the membranes were incubated with donkey anti-rabbit 800CW IRDye (1:15,000, Li-COR Biosciences) for 1 h at RT. After three final washes, the membranes were recorded on the Li-Cor Odyssey CLx imager or iBright FL1500 Imaging System (Thermo Fisher Scientific). Signal comparison for bands of full-length HTT or cleavage fragments of wt- and mut-HTT isoforms was conducted either against each other or normalized to the Calnexin signal of the same sample.

For protein analysis in MFDs, neurons were lysed with lysis buffer (0.5% Triton X-100, 150 mM NaCl, 50 mM HEPES, pH 7.32 with protease, and phosphatase inhibitors). For HTT silencing in human neurons, extracts form pre- and postsynaptic compartments were combined. For post-synaptic response after presynaptic stimulation, only the postsynaptic compartment was extracted. Laemmli buffer was added, and proteins were denatured at 95°C for 5 min. They were loaded on acrylamide gel (one well corresponding to one microfluidic device). After electrophoresis and transfer onto PVDF membranes previously activated with absolute ethanol, the membranes were blocked with 5% BSA in Tris-buffered Tween 20 (TBST) (25 mM Tris HCl (pH 7.5), 137 mM NaCl, 2.68 mM KCl and 0.1% Tween 20) for 1 h, and then incubated with antibodies directed against HTT (1:1,000; 5656S Ozyme), vinculin (1:10,000, V9131, Cell Signaling Technology), GluA1 (1:1,000; AB1504, Millipore), pAKT (1:1,000; 40585, Cell Signaling Technology), and pERK (1:2000, 9106, Cell Signaling Technology) overnight at 4°C. Membranes were washed three times for 10 min in TBST and incubated with a 1:5,000 horse-radish peroxidase-conjugated anti-mouse or anti-rabbit antibodies for 1 h at RT. Blots were washed three times with TBST for 10 min and revealed with ECL PRIME (Cytiva). Then, membranes containing phosphorylated proteins were stripped with stripping buffer (Thermo Fisher Scientific) for 20 min and re-blocked in 5% BSA in TBST. AKT (1:1,000, 9272, Cell Signaling Technology) and ERK (1:1,000, 9102 Cell Signaling Technology) antibodies were incubated overnight at 4°C on the same membranes as previously. Similar washing, secondary antibodies incubations and ECL PRIME revelation were done.

HTRF measurement of total and mut-HTT

Total- and mut-HTT levels in HD-iPSC-derived neuronal cultures were quantified using HTRF assays following the manufacturer's

instructions (HTRF HTT Mutant Kit #64HTTMPEG, HTT Total Kit #64HTTTPEG, Revvity). Briefly, cells were lysed directly in 96-well culture plates using 30 μL/well of PBS supplemented with 0.1% Triton X-100 and incubated for 15 min at 4 °C. Lysates were then stored at −80°C for at least 2 h prior to analysis. Ten microliters of each lysate were transferred to low-volume HTRF 96-well plates (Revvity). Ten microliters of the antibody mix were added, and plates were sealed and incubated at RT protected from light for 24–48 h prior to reading. HTRF signals were acquired using a CLARIOstar Plus microplate reader (BMG Labtech) with the following settings: excitation filter EX-TR, emission filters 620 nm (10 nm bandwidth) and 665 nm (10 nm bandwidth), dichroic mirror LP-TR, integration delay 60 μs, integration time 400 μs, 200 flashes per well, and a gain set of 2,400. Raw data were exported using MARS software.

CellTiter-Glo measurement of ATP level

Cellular ATP levels were measured as a readout of viability using the CellTiter-Glo 2.0 assay (Promega). Lysates (same extract than for the HTRF assays) prepared in PBS +0.1% Triton X-100 were equilibrated to RT for 30 min along with the CTG reagent, protected from light. Three microliters of each lysate were transferred to black 96-well plates (Greiner) diluted with 37 μL of PBS +0.1% Triton X-100. CTG reagent (40 μL/well) was added to all wells and plates were then incubated for 10 min at RT in the dark to stabilize luminescent signals. Luminescence was measured using a GloMax plate reader (Promega) with an integration time of 0.8 s per well.

RNA preparation

Total RNAs were extracted from neuronal cultures using the RNeasy Plus Micro kit (QIAGEN), following the manufacturer's instructions, with RNA elution in 14 μL. Neuronal cultures were incubated in 350 μL RLT Plus buffer, scraped, and vortexed for 30 s. The lysates were then placed in gDNA eliminator spin columns provided by the kit and centrifuged for 30 s at 13,000 *g*. Subsequently, one volume (350 μL) of 70% ethanol was added to the flow-throughs. The samples were transferred to RNeasy MinElute spin columns and centrifuged for 15 s at 13,000 *g*. RW1 buffer (700 μL) was added to the columns, followed by centrifugation for 15 s at 13,000 *g*. RPE buffer (500 μL) was added to the columns, and the whole mixture was centrifuged for another 15 s at 13,000 *g*. Subsequently, 80% ethanol (500 μL) was added to the columns, and the mixture was centrifuged for 2 min at 13,000 *g* and again for 5 min at 13,000 g with the lids open to allow drying. Finally, RNAs were eluted with 14 μL RNase-free water added to the center of the column membranes, followed by a final centrifugation of 1 min at 13,000 *g*. The quantity of RNA in each sample was determined using a NanoDrop spectrophotometer (Thermo Fisher Scientific).

RT-qPCR

Complementary DNA (cDNAs) were synthesized from 100 ng of total RNA for each sample using the PrimeScript RT reagent kit with gDNA eraser (Takara Bio). Genomic DNA (gDNA) was eliminated from the RNA samples by adding 2 μL gDNA Eraser Buffer and 1 μL gDNA Eraser enzyme provided by the Takara kit. The samples were incubated in a thermal cycler for 2 min at 42°C and then centrifuged. Reverse transcription reactions were prepared with 4 μL PrimeScript buffer, 1 μL PrimeScript RT enzyme mix, 1 μL RT primer mix, and 4 μL RNase-free water added to the samples. The samples were incubated in a thermal cycler for 15 min at 37°C and 30 s at 85°C.

For the SYBR Green reverse-transcription PCR (RT-PCR) quantification, cDNAs were diluted 1:20 with UltraPure distilled water (Thermo Fisher Scientific) to a final concentration of 250 pg/μL. PCR amplification was performed using iTaq universal SYBR Green Supermix (Bio-Rad Laboratories). The PCR mix included 4 μL iTaq SYBR Green supermix, 0.52 μL H2O, and 0.24 μL (from 10 μM stock) of specific primers for *HTTe6465*, *HTTe1415*, *PPIA*, and *hRPL13*. The PCR mix was added to the wells of the FrameStar 384 wells PCR plate (4titude). Three microliters of diluted cDNAs were loaded into each test well, and the plate was centrifuged for 1 min at 2,000 *g*. PCR was conducted on a CFX384 Real-Time System (C1000 Touch Thermal Cycler, Bio-Rad Laboratories) and analyzed with CFX Maestro software. iTaq supermix was activated at 95°C for 3 min, followed by 40 cycles of 10 s at 95°C and 30 s at 60°C. The final step involved amplicon dissociation from 65°C to 95°C. The median Ct of each gene (3 technical replicates) was calculated, and the results were expressed as the relative expression of each gene normalized to the reference gene h*RPL13*, whose stability across treatments was verified using *PPIA* (Figure S2A). **Primer sequences:** HTTe6465: 5′-AGT GAT TGT TGC TAT GGA GCG G-3′ and 5′-GCT GCT GGT TGG ACA GAA ACT C-3; HTTe1415: 5′-CAA GCC TTG CCG CAT CAA AG-3′ and 5′- CCC CCT GTT AGC AAA AAC GAA-3′; PPIA: 5′-TGG TGT TTG GCA AAG TGA AA-3′ and 5′-TGT CCA CAG TCA GCA ATG GT-3′; hRPL13: 5′-CCT GGA GGA GAA GAG GAA AGA GA-3′ and 5′-TTG AGG ACC TCT GTG TAT TTG TCA A-3′. For the TaqMan probe method, PCR was performed for allelic ratio determination using TaqPath ProAmp Master Mix (Thermo Fisher Scientific) combined with TaqMan SNP Genotyping assays (Thermo Fisher Scientific). The targeted SNPs were located in exon 50 (rs362331) and in exon 67 (rs362307) of *HTT*. The PCR mix included 10 ng of cDNA (same as used for RT-qPCR), 2.5 μL TaqMan master mixes, 0.125 μL of 40× SNP genotyping assay, and 1.375 μL of H2O. The median of the Ct difference (ΔCt = Ct_probe 1 [mut allele] – Ct_probe 2 [wt allele]) of each targeted allele (3 technical replicates) was used to calculate the *HTT* allelic ratio (allelic ratio Rmut/wt = $2^{(-\Delta Ct)}$). The results were normalized to the median allelic ratio of the samples treated with the NTC ASO. The TaqMan SNP genotyping assays were used to assess relative changes in the mutant-to-wild-type HTT transcript ratio rather than the absolute abundance of either allele. Allelic ratios were therefore normalized to the NTC-treated condition to minimize the influence of potential differences in amplification efficiency between the two allele-specific probes.

Calcium imaging of coordinated activity of neuronal network

To assess spontaneous neuronal network activity, human cortical neurons were plated in 96-well plates at a density of 150,000 cells/cm^2. Intracellular calcium dynamics were monitored using the

calcium-sensitive dye CAL520-AM (Abcam). At days *in vitro* (DIV) 28–35, neurons were incubated with 2 μM CAL520-AM diluted in Hanks' Balanced Salt Solution (HBSS) with calcium and magnesium (Gibco) supplemented with HEPES for 45 min at 37°C in 5% CO_2, followed by a 15 min incubation in HBSS without calcium and magnesium supplemented with HEPES, 0.1 mM $MgCl_2$ and 3 mM $CaCl_2$ (Gibco) under the same conditions. Fluorescence signals were acquired using the FDSS/μCELL plate reader (Hamamatsu Photonics) for 10 min at 37°C, beginning 5 min after plate placement in the recording chamber. Following acquisition, the recording buffer was replaced with BrainPhys medium (STEMCELL Technologies) supplemented with cytokines. Calcium transients were analyzed as relative fluorescence intensity changes ($\Delta F/F_0$). Quantified parameters included AMP, defined as the mean of the top 1% of $\Delta F/F_0$ peak AMPs over 10 min; frequency (peaks/min), calculated from the number of oscillations per minute with peaks defined by $\Delta F/F_0 > 0.1$; the coefficient of variation of amplitude (CV-AMP), corresponding to the mean CV of maximal $\Delta F/F_0$ AMPs; and interpeak time variability (CV peak-to-peak time), calculated as the CV of inter-event intervals over the recording duration.

Protein samples preparation for mass spectrometry

Protein extracts were diluted in RIPA lysis buffer (Thermo Fisher Scientific) to a final concentration of 0.3 μg/μL. For denaturation, 40 μL of protein extract (12 μg total protein) was mixed with 40 μL of 2× LDS Bolt buffer (Life Technologies) containing 1× reducing agent (Life Technologies), followed by incubation at 90°C for 10 min. Samples were stored at −80°C until proteolytic digestion. Tryptic digestion was performed using the Single-Pot Solid-Phase-enhanced Sample Preparation (SP3) method, as previously described[18] and adapted from[67] with prior denaturation in Laemmli buffer. After reduction alkylation as described,[18] tryptic digestion was performed by addition in 100 μL of sequencing-grade trypsin (Trypsin Gold, Promega; 6 ng/μLin 50 mM ammonium bicarbonate, pH 8.0) and overnight incubation at 37°C with agitation (1,000 rpm). After centrifugation (5 min, 4°C, 15,000 rpm), tryptic peptides were recovered from the supernatant using a magnetic rack and stored at −80°C until liquid chromatography-tandem mass spectrometry (LC-MS/MS) analysis.

MS analysis

Tryptic peptide mixtures (0.25 μg in 2 μL) were analyzed by nanoLC-MS/MS using a NanoElut e2 system (Bruker) coupled to a timsTOF Pro 2 mass spectrometer (Bruker). Peptides were analyzed using exactly the same chromatographic peptide trapping and analytical separation methods, and the same mass spectrometry parameters for MS/MS fragmentation in the PASEF (parallel accumulation-serial fragmentation) data-dependent acquisition mode than previously described.[18] Raw data were processed and converted to .mgf files using Bruker DataAnalysis software. Protein identification was performed with Mascot v.2.6.2 (Matrix Science) against the SwissProt Human database (release 2022_02; 42,363 entries), using a trypsin specificity with up to 2 missed cleavages, peptide, and fragment tolerances of 15 ppm and 0.05 Da, respectively and following modifications; carbamidomethylation of cysteine and methionine oxidation set as fixed and variable modifications, respectively. Decoy database searches were included. Peptide and protein identifications were validated using Scaffold v.5.0.1 (Proteome Software) with probabilities >91% and >96%, respectively, to achieve false discovery rate (FDR) <0.1%, as calculated by the Percolator algorithm. Protein probabilities were assigned by Protein Prophet, and indistinguishable proteins were grouped by parsimony. Data were filtered to include proteins identified by $\geq$ 2 peptides at $\geq$ 95% probability and FDR <0.1%. Label-free quantification (LFQ) was performed using both spectral counting (Scaffold) and extracted ion current (XIC)-based intensity (MaxQuant v.2.0.3.0). XIC-based quantification employed the Andromeda search engine with parameters identical to those described earlier. Relative protein abundances were determined using the maxLFQ algorithm with default normalization and four-dimensional feature alignment, including collision cross section (CCS) matching.

Statistical analyses were performed as previously described[18] on four or five biological replicates. In scaffold for spectral counts and in Perseus v.1.6.6 for MaxLFQ data. LFQ intensities were $\log_2$-transformed, and categorical annotations applied for the experimental groups (SNP, PAN, NTC, and PBS). Proteins were retained for further quantification if detected in $\geq$3 replicates within at least one condition. Analyses were conducted with and without missing value imputation using low-intensity random sampling. ANOVA with Benjamini-Hochberg FDR $\leq$0.05 identified significantly regulated proteins. Quality control included principal-component analysis (PCA) and hierarchical clustering (Pearson's correlation) based on Z score-normalized intensities. Fold-change analyses were generated from maxLFQ data, and proteins with p values $\leq$0.05 and absolute fold-change $\geq$1.25 were considered significantly altered between conditions.

Proteomic data deposition

The mass spectrometric proteomics datasets have been deposited at the ProteomeXchange Consortium via the PRIDE[68] partner repository (http://proteomecentral.proteomexchange.org) with the dataset identifiers PXD xxx (https://www.ebi.ac.uk/pride/archive/projects/PXD xxx).

Live-cell and confocal imaging in microfluidic chambers

Acquisitions for BDNF trafficking were performed using an inverted microscope (Axio Observer, Zeiss) coupled to a spinning-disk confocal system (CSU-W1-T3, Yokogawa) connected to a wide-field electron-multiplying CCD camera (ProEM+1024, Princeton Instrument) maintained at 37°C and 5% CO_2. For the study of BDNF transport, images were captured every 200 ms for 30 s to follow BDNF-mCherry vesicles (×63 oil-immersion objective, 1.46 NA) in the axonal microchannels. Kymographs were generated using the KymoToolBox plugin for ImageJ,[59] with a length of 100 μm (x axis) and a total time of 30 s (y axis).

For characterization of the human network and synaptic density study, images were acquired into compartment of interest using an

inverted confocal microscope (LSM 710, Zeiss) with a 20× objective (0.8 NA) or a 68× immersion objective (1.4 NA) coupled to an Airyscan detector. HuC/HuD, MAP2, TBR1, or VGLUT2 positive cells were counted manually on ImageJ software and were reported to the total number of cells using Hoechst staining. Results were expressed as a percentage of immunopositive cells. Homer/Bassoon counting was performed on ICY software (https://icy.bioimageanalysis.org; De Chaumont et al.[69]). The number of Homer and Bassoon spots as well as the number of adjacent spots were automatically counted with the plugin ICY SODA.[70] The dimension of each field analyzed corresponds to 65.89 μm per 65.89 μm.

Automated cell imaging

Images were acquired using the HCS CellInsight CX7 High Content Screening Platform (Thermo Fisher Scientific) from 25 randomly selected fields per well in 96-well plates. All experiments were performed in at least three independent replicates. Image analyses were conducted using the Cellomics Bioapplication (v.4.2). Nuclei were segmented following uniform smoothing correction and threshold-based intensity segmentation. Nuclei-to-cell body pairing was performed using the "MinimumCellBodyNucleusOverlap" parameter. Cell bodies were defined by $MAP2^+$ morphology combined with fixed threshold intensity criteria.

Quantification and statistical analyses

All statistical analyses were performed using GraphPad Prism (GraphPad Software, Inc.) software. All experiments consisted of at least three independent replicates and were conducted blindly. All statistical analyses were performed using GraphPad Prism (GraphPad Software, Inc.) software. Experiments were independently repeated at least three times. For automated microscopy analyses, each independent experiment included at least six replicate wells per treatment condition. Twenty-five fields were acquired and analyzed per well, and measurements from individual cells and fields were aggregated to generate a single value for each well. Thus, individual cells and fields were considered subsamples and were not treated as independent observations. Statistical analyses were performed using wells as the experimental units, pooling wells from the independent experiments.

The normality of the data distribution was tested by performing D'Agostino and Pearson's test with threshold set at $\alpha = 0.05$. When the *n* was too small to be analyzed by a normality test (e.g., western blotting analyses), the normality was assumed. When comparing two groups, we used the unpaired two-tailed Student's *t* test when the data were normally distributed or the Mann-Whitney test when the data were not normally distributed. When comparing multiple groups, we used one-way analysis of variance (ANOVA) followed by Tukey's post hoc analysis when the data were normally distributed or Kruskal-Wallis test followed by a Dunn's post hoc analysis when the data were not normally distributed. When comparing multiple groups longitudinally, we used two-way ANOVA followed by Tukey's post hoc analysis. Data are expressed as means ± SEM and are presented as the means. Statistical significance: ns non-significant for p value >0.05, *p value $\leq$0.05, **p value $\leq$0.01, ***p value $\leq$0.001.

DATA AND CODE AVAILABILITY

Data will be made available on request. The mass spectrometry proteomics data have been deposited to the ProteomeXchange Consortium[71] via the PRIDE[72] partner repository with the dataset identifier PXD084081 and https://doi.org/10.6019/PXD084081.

ACKNOWLEDGMENTS

The authors are grateful to the scientific team at Wave Life Sciences for design, synthesis, and validation of NTC, PAN, and SNP ASO. We acknowledge the Proteomic-Gif (SICaPS) platform (supported by IBiSA, Ile de France Region, Plan Cancer, and CNRS and Paris-Saclay University).

The present research is supported by Wave Life Sciences (A.L.P. and F.S.); R.A.L. was supported by the contract with Wave Life Sciences and FRM (FDT20230401658). A.L. was supported by the contract with Wave Life Sciences and the French Investissements d'Avenir Program NeurATRIS (ANR-11-INBS-0011 to A.L.P.). L.C. was supported by the contract with Wave Life Sciences. M.L. received salary support from the Association Huntington France (AHF), the national "Infrastructure de recherche" NeurATRIS, and the CEA. This work was partly funded by the Agence Nationale de la Recherche (Laboratoire d'Excellence Revive, Investissement d'Avenir; ANR-10-LABX- 73, to A.L.P.), the French Investissements d'Avenir Program NeurATRIS (ANR-11-INBS-0011 and ANR-24-INBS-0013 to A.L.P.), ANR-22-CE17-0044-01 SAVEHD (to F.S.) and Fondation pour la Recherche Médicale, FRM, EQ202403018074 (to F.S.). A.L.P. and V.R. are employees of the Institut national de la santé et de la recherche médicale (Inserm). F.S. is an employee of Univ. Grenoble Alpes (UGA) and University Hospital Grenoble Alpes (CHUGA). Y.L., A.M., N.I., M.B., and C.V. are employees of Wave Life Sciences.

AUTHOR CONTRIBUTIONS

R.A.L., A.L., and L.C. contributed equally to this work. A.L.P. and F.S. jointly supervised this work and share correspondence responsibilities. A.L.P. and F.S. conceived the study. A.L., L.C., and D.E.A. generated iPSC-derived neuronal cultures. R.A.L. and S.L. performed microfluidic and synaptic assays. A.L. and L.C. conducted biochemical analyses. A.L., L.C., D.E.A., and M.L. conducted functional and immunofluorescence analyses of neuronal cultures. H.J.-J. and V.R. performed proteomic analyses. A.L.P., F.S., R.A.L., A.L., L.C., and S.L. performed data analysis and statistics. A.L.P., F.S., R.A.L., A.L., and L.C. wrote the manuscript. Y.L., A.M., N.I., M.B., and C.V., reviewed and edited the manuscript. A.L.P. and F.S. provided supervision and funding. All authors reviewed and approved the manuscript.

DECLARATION OF INTERESTS

This work was funded in part by Wave Life Sciences. A.L.P. has provided paid consulting and conducted sponsored research for Wave Life Sciences. F.S. is a co-founder of and holds shares in HuntX Pharma and has provided paid consulting and conducted sponsored research for Wave Life Sciences.

Y.L., N.I., M.B., and C.V. are employees and shareholders of Wave Life Sciences, and have a patent related to this work. A.M. is an employee and shareholder of Wave Life Sciences.

SUPPLEMENTAL INFORMATION

Supplemental information can be found online at https://doi.org/10.1016/j.omtn.2026.103160.

REFERENCES

1. Ross, C.A., and Tabrizi, S.J. (2011). Huntington's disease: from molecular pathogenesis to clinical treatment. Lancet Neurol. *10*, 83–98. https://doi.org/10.1016/S1474-4422(10)70245-3.
2. Farag, M., Tabrizi, S.J., and Wild, E.J. (2026). Huntington's disease clinical trials update: October 2025. J. Huntingtons Dis. *15*, 156–166. https://doi.org/10.1177/18796397251399751.

3. The Huntington's Disease Collaborative Research (1993). A novel gene containing a trinucleotide repeat that is expanded and unstable on Huntington's disease chromosomes. The Huntington's Disease Collaborative Research Group. Cell *72*, 971–983.
4. Zuccato, C., Tartari, M., Crotti, A., Goffredo, D., Valenza, M., Conti, L., Cataudella, T., Leavitt, B.R., Hayden, M.R., Timmusk, T., et al. (2003). Huntingtin interacts with REST/NRSF to modulate the transcription of NRSE-controlled neuronal genes. Nat. Genet. *35*, 76–83. https://doi.org/10.1038/ng1219.
5. Gauthier, L.R., Charrin, B.C., Borrell-Pagès, M., Dompierre, J.P., Rangone, H., Cordelières, F.P., De Mey, J., MacDonald, M.E., Lessmann, V., Humbert, S., and Saudou, F. (2004). Huntingtin controls neurotrophic support and survival of neurons by enhancing BDNF vesicular transport along microtubules. Cell *118*, 127–138. https://doi.org/10.1016/j.cell.2004.06.018.
6. Cepeda, C., and Levine, M.S. (2022). Synaptic Dysfunction in Huntington's Disease: Lessons from Genetic Animal Models. Neuroscientist *28*, 20–40. https://doi.org/10.1177/1073858420972662.
7. Saudou, F., and Humbert, S. (2016). The Biology of Huntingtin. Neuron *89*, 910–926. https://doi.org/10.1016/j.neuron.2016.02.003.
8. Altar, C.A., Cai, N., Bliven, T., Juhasz, M., Conner, J.M., Acheson, A.L., Lindsay, R.M., and Wiegand, S.J. (1997). Anterograde transport of brain-derived neurotrophic factor and its role in the brain. Nature *389*, 856–860. https://doi.org/10.1038/39885.
9. Baquet, Z.C., Gorski, J.A., and Jones, K.R. (2004). Early striatal dendrite deficits followed by neuron loss with advanced age in the absence of anterograde cortical brain-derived neurotrophic factor. J. Neurosci. *24*, 4250–4258. https://doi.org/10.1523/JNEUROSCI.3920-03.2004.
10. Zuccato, C., and Cattaneo, E. (2007). Role of brain-derived neurotrophic factor in Huntington's disease. Prog. Neurobiol. *81*, 294–330. https://doi.org/10.1016/j.pneurobio.2007.01.003.
11. Zuccato, C., Ciammola, A., Rigamonti, D., Leavitt, B.R., Goffredo, D., Conti, L., MacDonald, M.E., Friedlander, R.M., Silani, V., Hayden, M.R., et al. (2001). Loss of huntingtin-mediated BDNF gene transcription in Huntington's disease. Science *293*, 493–498. https://doi.org/10.1126/science.1059581.
12. Caviston, J.P., and Holzbaur, E.L.F. (2009). Huntingtin as an essential integrator of intracellular vesicular trafficking. Trends Cell Biol. *19*, 147–155. https://doi.org/10.1016/j.tcb.2009.01.005.
13. Dragatsis, I., Levine, M.S., and Zeitlin, S. (2000). Inactivation of Hdh in the brain and testis results in progressive neurodegeneration and sterility in mice. Nat. Genet. *26*, 300–306. https://doi.org/10.1038/81593.
14. Kotowska-Zimmer, A., Przybyl, L., Pewinska, M., Suszynska-Zajczyk, J., Wronka, D., Figiel, M., and Olejniczak, M. (2022). A CAG repeat-targeting artificial miRNA lowers the mutant huntingtin level in the YAC128 model of Huntington's disease. Mol. Ther. Nucleic Acids *28*, 702–715. https://doi.org/10.1016/j.omtn.2022.04.031.
15. Tabrizi, S.J., Ghosh, R., and Leavitt, B.R. (2019). Huntingtin Lowering Strategies for Disease Modification in Huntington's Disease. Neuron *101*, 801–819. https://doi.org/10.1016/j.neuron.2019.01.039.
16. Tabrizi, S.J., Estevez-Fraga, C., van Roon-Mom, W.M.C., Flower, M.D., Scahill, R.I., Wild, E.J., Muñoz-Sanjuan, I., Sampaio, C., Rosser, A.E., and Leavitt, B.R. (2022). Potential disease-modifying therapies for Huntington's disease: lessons learned and future opportunities. Lancet Neurol. *21*, 645–658. https://doi.org/10.1016/S1474-4422(22)00121-1.
17. Burrus, C.J., McKinstry, S.U., Kim, N., Ozlu, M.I., Santoki, A.V., Fang, F.Y., Ma, A., Karadeniz, Y.B., Worthington, A.K., Dragatsis, I., et al. (2020). Striatal Projection Neurons Require Huntingtin for Synaptic Connectivity and Survival. Cell Rep. *30*, 642–657.e6. https://doi.org/10.1016/j.celrep.2019.12.069.
18. Louçã, M., El Akrouti, D., Lemesle, A., Louessard, M., Dufour, N., Baroin, C., de la Fouchardière, A., Cotter, L., Jean-Jacques, H., Redeker, V., and Perrier, A.L. (2024). Huntingtin lowering impairs the maturation and synchronized synaptic activity of human cortical neuronal networks derived from induced pluripotent stem cells. Neurobiol. Dis. *200*, 106630. https://doi.org/10.1016/j.nbd.2024.106630.
19. Borowsky, B., Ramos, H., Caputo, A., Hartmann, A., Faller, T., Peters, T., Sui, Y., Liu, F., Meadowcroft, M., David, O.J., et al. (2026). Oral splicing modulator branaplam in Huntington's disease: a phase 2 randomized controlled trial. Nat. Med. *32*, 103–112. https://doi.org/10.1038/s41591-025-04117-4.
20. Drouet, V., Ruiz, M., Zala, D., Feyeux, M., Auregan, G., Cambon, K., Troquier, L., Carpentier, J., Aubert, S., Merienne, N., et al. (2014). Allele-Specific Silencing of Mutant Huntingtin in Rodent Brain and Human Stem Cells. PLoS One *9*, e99341. https://doi.org/10.1371/journal.pone.0099341.
21. Borrell-Pagès, M., Canals, J.M., Cordelières, F.P., Parker, J.A., Pineda, J.R., Grange, G., Bryson, E.A., Guillermier, M., Hirsch, E., Hantraye, P., et al. (2006). Cystamine and cysteamine increase brain levels of BDNF in Huntington disease via HSJ1b and transglutaminase. J. Clin. Investig. *116*, 1410–1424. https://doi.org/10.1172/JCI27607.
22. Pardo, R., Colin, E., Régulier, E., Aebischer, P., Déglon, N., Humbert, S., and Saudou, F. (2006). Inhibition of Calcineurin by FK506 Protects against Polyglutamine-Huntingtin Toxicity through an Increase of Huntingtin Phosphorylation at S421. J. Neurosci. *26*, 1635–1645. https://doi.org/10.1523/JNEUROSCI.3706-05.2006.
23. Zala, D., Colin, E., Rangone, H., Liot, G., Humbert, S., and Saudou, F. (2008). Phosphorylation of mutant huntingtin at S421 restores anterograde and retrograde transport in neurons. Hum. Mol. Genet. *17*, 3837–3846. https://doi.org/10.1093/hmg/ddn281.
24. Pineda, J.R., Pardo, R., Zala, D., Yu, H., Humbert, S., and Saudou, F. (2009). Genetic and pharmacological inhibition of calcineurin corrects the BDNF transport defect in Huntington's disease. Mol. Brain *2*, 33. https://doi.org/10.1186/1756-6606-2-33.
25. Verny, C., Bachoud-Lévi, A.-C., Durr, A., Goizet, C., Azulay, J.-P., Simonin, C., Tranchant, C., Calvas, F., Krystkowiak, P., Charles, P., et al. (2017). A randomized, double-blind, placebo-controlled trial evaluating cysteamine in Huntington's disease: Trial Evaluating Cysteamine in HD. Mov. Disord. *32*, 932–936. https://doi.org/10.1002/mds.27010.
26. Virlogeux, A., Scaramuzzino, C., Lenoir, S., Carpentier, R., Louessard, M., Genoux, A., Lino, P., Hinckelmann, M.-V., Perrier, A.L., Humbert, S., and Saudou, F. (2021). Increasing brain palmitoylation rescues behavior and neuropathology in Huntington disease mice. Sci. Adv. *7*, eabb0799. https://doi.org/10.1126/sciadv.abb0799.
27. Iwamoto, N., Liu, Y., Frank-Kamenetsky, M., Maguire, A., Tseng, W.C., Taborn, K., Kothari, N., Akhtar, A., Bowman, K., Shelke, J.D., et al. (2024). Preclinical evaluation of stereopure antisense oligonucleotides for allele-selective lowering of mutant HTT. Mol. Ther. Nucleic Acids *35*, 102246. https://doi.org/10.1016/j.omtn.2024.102246.
28. Skotte, N.H., Southwell, A.L., Østergaard, M.E., Carroll, J.B., Warby, S.C., Doty, C.N., Petoukhov, E., Vaid, K., Kordasiewicz, H., Watt, A.T., et al. (2014). Allele-Specific Suppression of Mutant Huntingtin Using Antisense Oligonucleotides: Providing a Therapeutic Option for All Huntington Disease Patients. PLoS One *9*, e107434. https://doi.org/10.1371/journal.pone.0107434.
29. Bragg, R.M., Landles, C., Smith, E.J., Osborne, G.F., Mathews, E.W., Cantle, J.P., Bates, G.P., and Carroll, J.B. (2026). Selective targeting of mutant huntingtin intron 1 improves rescue provided by antisense oligonucleotides in Huntington's disease mice. Sci. Transl. Med. *18*, eadv0702. https://doi.org/10.1126/scitranslmed.adv0702.
30. HD iPSC Consortium (2012). Induced pluripotent stem cells from patients with Huntington's disease show CAG-repeat-expansion-associated phenotypes. Cell Stem Cell *11*, 264–278. https://doi.org/10.1016/j.stem.2012.04.027.
31. (2020). Bioenergetic deficits in Huntington's disease iPSC-derived neural cells and rescue with glycolytic metabolites. Hum. Mol. Genet. *29*, 1757–1771. https://doi.org/10.1093/hmg/ddy430.
32. Mehta, S.R., Tom, C.M., Wang, Y., Bresee, C., Rushton, D., Mathkar, P.P., Tang, J., and Mattis, V.B. (2018). Human Huntington's Disease iPSC-Derived Cortical Neurons Display Altered Transcriptomics, Morphology, and Maturation. Cell Rep. *25*, 1081–1096.e6. https://doi.org/10.1016/j.celrep.2018.09.076.
33. Wu, G.-H., Smith-Geater, C., Galaz-Montoya, J.G., Gu, Y., Gupte, S.R., Aviner, R., Mitchell, P.G., Hsu, J., Miramontes, R., Wang, K.Q., et al. (2023). CryoET reveals organelle phenotypes in huntington disease patient iPSC-derived and mouse primary neurons. Nat. Commun. *14*, 692. https://doi.org/10.1038/s41467-023-36096-w.

34. Louessard, M., Cailleret, M., Jarrige, M., Bigarreau, J., Lenoir, S., Dufour, N., Rey, M., Saudou, F., Deglon, N., and Perrier, A.L. (2024). Mono- and Biallelic Inactivation of Huntingtin Gene in Patient-Specific Induced Pluripotent Stem Cells Reveal HTT Roles in Striatal Development and Neuronal Functions. J. Huntingtons Dis. *13*, 41–53. https://doi.org/10.3233/JHD-231509.

35. Iwamoto, N., Butler, D.C.D., Svrzikapa, N., Mohapatra, S., Zlatev, I., Sah, D.W.Y., Meena, Standley, S.M., Lu, G., Apponi, L.H., et al. (2017). Control of phosphorothioate stereochemistry substantially increases the efficacy of antisense oligonucleotides. Nat. Biotechnol. *35*, 845–851. https://doi.org/10.1038/nbt.3948.

36. Kandasamy, P., McClorey, G., Shimizu, M., Kothari, N., Alam, R., Iwamoto, N., Kumarasamy, J., Bommineni, G.R., Bezigian, A., Chivatakarn, O., et al. (2022). Control of backbone chemistry and chirality boost oligonucleotide splice switching activity. Nucleic Acids Res. *50*, 5443–5466. https://doi.org/10.1093/nar/gkac018.

37. Chen, E.Y., Tan, C.M., Kou, Y., Duan, Q., Wang, Z., Meirelles, G.V., Clark, N.R., and Ma'ayan, A. (2013). Enrichr: interactive and collaborative HTML5 gene list enrichment analysis tool. BMC Bioinf. *14*, 128. https://doi.org/10.1186/1471-2105-14-128.

38. Kuleshov, M.V., Jones, M.R., Rouillard, A.D., Fernandez, N.F., Duan, Q., Wang, Z., Koplev, S., Jenkins, S.L., Jagodnik, K.M., Lachmann, A., et al. (2016). Enrichr: a comprehensive gene set enrichment analysis web server 2016 update. Nucleic Acids Res. *44*, W90–W97. https://doi.org/10.1093/nar/gkw377.

39. Xie, Z., Bailey, A., Kuleshov, M.V., Clarke, D.J.B., Evangelista, J.E., Jenkins, S.L., Lachmann, A., Wojciechowicz, M.L., Kropiwnicki, E., Jagodnik, K.M., et al. (2021). Gene Set Knowledge Discovery with Enrichr. Curr. Protoc. *1*, e90. https://doi.org/10.1002/cpz1.90.

40. Lopes, C., Tang, Y., Anjo, S.I., Manadas, B., Onofre, I., de Almeida, L.P., Daley, G.Q., Schlaeger, T.M., and Rego, A.C.C. (2020). Mitochondrial and Redox Modifications in Huntington Disease Induced Pluripotent Stem Cells Rescued by CRISPR/Cas9 CAGs Targeting. Front. Cell Dev. Biol. *8*, 576592. https://doi.org/10.3389/fcell.2020.576592.

41. White, J.A., Krzystek, T.J., Hoffmar-Glennon, H., Thant, C., Zimmerman, K., Iacobucci, G., Vail, J., Thurston, L., Rahman, S., and Gunawardena, S. (2020). Excess Rab4 rescues synaptic and behavioral dysfunction caused by defective HTT-Rab4 axonal transport in Huntington's disease. Acta Neuropathol. Commun. *8*, 97. https://doi.org/10.1186/s40478-020-00964-z.

42. Pal, A., Severin, F., Lommer, B., Shevchenko, A., and Zerial, M. (2006). Huntingtin-HAP40 complex is a novel Rab5 effector that regulates early endosome motility and is up-regulated in Huntington's disease. J. Cell Biol. *172*, 605–618. https://doi.org/10.1083/jcb.200509091.

43. Ravikumar, B., Imarisio, S., Sarkar, S., O'Kane, C.J., and Rubinsztein, D.C. (2008). Rab5 modulates aggregation and toxicity of mutant huntingtin through macroautophagy in cell and fly models of Huntington disease. J. Cell Sci. *121*, 1649–1660. https://doi.org/10.1242/jcs.025726.

44. White, J.A., Anderson, E., Zimmerman, K., Zheng, K.H., Rouhani, R., and Gunawardena, S. (2015). Huntingtin differentially regulates the axonal transport of a sub-set of Rab-containing vesicles in vivo. Hum. Mol. Genet. *24*, 7182–7195. https://doi.org/10.1093/hmg/ddv415.

45. Virlogeux, A., Moutaux, E., Christaller, W., Genoux, A., Bruyère, J., Fino, E., Charlot, B., Cazorla, M., and Saudou, F. (2018). Reconstituting Corticostriatal Network on-a-Chip Reveals the Contribution of the Presynaptic Compartment to Huntington's Disease. Cell Rep. *22*, 110–122. https://doi.org/10.1016/j.celrep.2017.12.013.

46. Lenoir, S., Lahaye, R.A., Vitet, H., Scaramuzzino, C., Virlogeux, A., Capellano, L., Genoux, A., Gershoni-Emek, N., Geva, M., Hayden, M.R., and Saudou, F. (2022). Pridopidine rescues BDNF/TrkB trafficking dynamics and synapse homeostasis in a Huntington disease brain-on-a-chip model. Neurobiol. Dis. *173*, 105857. https://doi.org/10.1016/j.nbd.2022.105857.

47. Saade, J., and Mestre, T.A. (2024). Huntington's Disease: Latest Frontiers in Therapeutics. Curr. Neurol. Neurosci. Rep. *24*, 255–264. https://doi.org/10.1007/s11910-024-01345-y.

48. Kandasamy, P., Liu, Y., Aduda, V., Akare, S., Alam, R., Andreucci, A., Boulay, D., Bowman, K., Byrne, M., Cannon, M., et al. (2022). Impact of guanidine-containing backbone linkages on stereopure antisense oligonucleotides in the CNS. Nucleic Acids Res. *50*, 5401–5423. https://doi.org/10.1093/nar/gkac037.

49. Monk, R., Lee, K., Jones, K.S., and Connor, B. (2021). Directly Reprogrammed Huntington's Disease Neural Precursor Cells Generate Striatal Neurons Exhibiting Aggregates and Impaired Neuronal Maturation. Stem Cell. *39*, 1410–1422. https://doi.org/10.1002/stem.3420.

50. Pecho-Vrieseling, E., Rieker, C., Fuchs, S., Bleckmann, D., Esposito, M.S., Botta, P., Goldstein, C., Bernhard, M., Galimberti, I., Müller, M., et al. (2014). Transneuronal propagation of mutant huntingtin contributes to non-cell autonomous pathology in neurons. Nat. Neurosci. *17*, 1064–1072. https://doi.org/10.1038/nn.3761.

51. Capizzi, M., Carpentier, R., Denarier, E., Adrait, A., Kassem, R., Mapelli, M., Couté, Y., and Humbert, S. (2022). Developmental defects in Huntington's disease show that axonal growth and microtubule reorganization require NUMA1. Neuron *110*, 36–50.e5. https://doi.org/10.1016/j.neuron.2021.10.033.

52. Villegas, L.D., Mathiesen, A.V., Rasmussen, I., von Broich, M., Qvist, F.L., Skotte, N., Bardile, C.F., Budtz-Jørgensen, E., Freude, K., Schmid, B., et al. (2025). A potential role for NADPH oxidase (NOX1/2) in mutant huntingtin-induced anomalous neurite outgrowth. Mol. Cell. Neurosci. *134*, 104012. https://doi.org/10.1016/j.mcn.2025.104012.

53. Xu, C., Chen, S., Chen, X., Ho, K.H., Park, C., Yoo, H., Lee, S.-H., and Park, H. (2023). Altered exocytosis of inhibitory synaptic vesicles at single presynaptic terminals of cultured striatal neurons in a knock-in mouse model of Huntington's disease. Front. Mol. Neurosci. *16*, 1175522. https://doi.org/10.3389/fnmol.2023.1175522.

54. Quintanilla, R.A., and Johnson, G.V.W. (2009). Role of Mitochondrial Dysfunction in the Pathogenesis of Huntington's Disease. Brain Res. Bull. *80*, 242–247. https://doi.org/10.1016/j.brainresbull.2009.07.010.

55. HD iPSC Consortium (2020). Bioenergetic deficits in Huntington's disease iPSC-derived neural cells and rescue with glycolytic metabolites. Hum. Mol. Genet. *29*, 1757–1771. https://doi.org/10.1093/hmg/ddy430.

56. Kwon, D. (2021). Failure of genetic therapies for Huntington's devastates community. Nature *593*, 180. https://doi.org/10.1038/d41586-021-01177-7.

57. Estevez-Fraga, C., Tabrizi, S.J., and Wild, E.J. (2024). Huntington's Disease Clinical Trials Corner: March 2024. J. Huntingtons Dis. *13*, 1–14. https://doi.org/10.3233/JHD-240017.

58. Liot, G., Zala, D., Pla, P., Mottet, G., Piel, M., and Saudou, F. (2013). Mutant Huntingtin alters retrograde transport of TrkB receptors in striatal dendrites. J. Neurosci. *33*, 6298–6309. https://doi.org/10.1523/JNEUROSCI.2033-12.2013.

59. Zala, D., Hinckelmann, M.-V., Yu, H., Lyra da Cunha, M.M., Liot, G., Cordelières, F.P., Marco, S., and Saudou, F. (2013). Vesicular glycolysis provides on-board energy for fast axonal transport. Cell *152*, 479–491. https://doi.org/10.1016/j.cell.2012.12.029.

60. Southwell, A.L., Kordasiewicz, H.B., Langbehn, D., Skotte, N.H., Parsons, M.P., Villanueva, E.B., Caron, N.S., Østergaard, M.E., Anderson, L.M., Xie, Y., et al. (2018). Huntingtin suppression restores cognitive function in a mouse model of Huntington's disease. Sci. Transl. Med. *10*, eaar3959. https://doi.org/10.1126/scitranslmed.aar3959.

61. Marchionini, D.M., Liu, J.-P., Ambesi-Impiombato, A., Kerker, K., Cirillo, K., Bansal, M., Mushlin, R., Brunner, D., Ramboz, S., Kwan, M., et al. (2022). Benefits of global mutant huntingtin lowering diminish over time in a Huntington's disease mouse model. JCI Insight *7*, e161769. https://doi.org/10.1172/jci.insight.161769.

62. Carroll, J.B., Warby, S.C., Southwell, A.L., Doty, C.N., Greenlee, S., Skotte, N., Hung, G., Bennett, C.F., Freier, S.M., and Hayden, M.R. (2011). Potent and selective antisense oligonucleotides targeting single-nucleotide polymorphisms in the Huntington disease gene/allele-specific silencing of mutant huntingtin. Mol. Ther. *19*, 2178–2185. https://doi.org/10.1038/mt.2011.201.

63. Miller, J.R.C., Pfister, E.L., Liu, W., Andre, R., Träger, U., Kennington, L.A., Lo, K., Dijkstra, S., Macdonald, D., Ostroff, G., et al. (2017). Allele-Selective Suppression of Mutant Huntingtin in Primary Human Blood Cells. Sci. Rep. *7*, 46740. https://doi.org/10.1038/srep46740.

64. Pfister, E.L., Kennington, L., Straubhaar, J., Wagh, S., Liu, W., DiFiglia, M., Landwehrmeyer, B., Vonsattel, J.-P., Zamore, P.D., and Aronin, N. (2009). Five

siRNAs targeting three SNPs may provide therapy for three-quarters of Huntington's disease patients. Curr. Biol. *19*, 774–778. https://doi.org/10.1016/j.cub.2009.03.030.

65. Gribaudo, S., Bousset, L., Courte, J., Melki, R., Peyrin, J.-M., and Perrier, A.L. (2023). Propagation of Distinct α-Synuclein Strains Within Human Reconstructed Neuronal Network and Associated Neuronal Dysfunctions. Methods Mol. Biol. *2551*, 357–378. https://doi.org/10.1007/978-1-0716-2597-2_24.

66. Lenoir, S., Genoux, A., Agasse, F., Saudou, F., and Humbert, S. (2021). Recreating mouse cortico-hippocampal neuronal circuit in microfluidic devices to study BDNF axonal transport upon glucocorticoid treatment. STAR Protoc. *2*, 100382. https://doi.org/10.1016/j.xpro.2021.100382.

67. Hughes, C.S., Moggridge, S., Müller, T., Sorensen, P.H., Morin, G.B., and Krijgsveld, J. (2019). Single-pot, solid-phase-enhanced sample preparation for proteomics experiments. Nat. Protoc. *14*, 68–85. https://doi.org/10.1038/s41596-018-0082-x.

68. Perez-Riverol, Y., Bai, J., Bandla, C., García-Seisdedos, D., Hewapathirana, S., Kamatchinathan, S., Kundu, D.J., Prakash, A., Frericks-Zipper, A., Eisenacher, M., et al. (2022). The PRIDE database resources in 2022: a hub for mass spectrometry-based proteomics evidences. Nucleic Acids Res. *50*, D543–D552. https://doi.org/10.1093/nar/gkab1038.

69. De Chaumont, F., Dallongeville, S., Chenouard, N., Hervé, N., Pop, S., Provoost, T., Meas-Yedid, V., Pankajakshan, P., Lecomte, T., Le Montagner, Y., et al. (2012). Icy: an open bioimage informatics platform for extended reproducible research. Nat. Methods *9*, 690–696. https://doi.org/10.1038/nmeth.2075.

70. Lagache, T., Grassart, A., Dallongeville, S., Faklaris, O., Sauvonnet, N., Dufour, A., Danglot, L., and Olivo-Marin, J.-C. (2018). Mapping molecular assemblies with fluorescence microscopy and object-based spatial statistics. Nat. Commun. *9*, 698. https://doi.org/10.1038/s41467-018-03053-x.

71. Deutsch, E.W., Bandeira, N., Perez-Riverol, Y., Sharma, V., Carver, J.J., Mendoza, L., Kundu, D.J., Bandla, C., Kamatchinathan, S., Hewapathirana, S., et al. (2026). The ProteomeXchange consortium in 2026: making proteomics data FAIR. Nucleic Acids Res. *54*, D459–D469. https://doi.org/10.1093/nar/gkaf1146.

72. Perez-Riverol, Y., Bandla, C., Kundu, D.J., Kamatchinathan, S., Bai, J., Hewapathirana, S., John, N.S., Prakash, A., Walzer, M., Wang, S., and Vizcaíno, J.A. (2025). The PRIDE database at 20 years: 2025 update. Nucleic Acids Res. *53*, D543–D553. https://doi.org/10.1093/nar/gkae1011.

OMTN, Volume *37*

Supplemental information

Allele-selective and pan-allelic huntingtin lowering differentially affect neuronal homeostasis and BDNF transport

Romane A. Lahaye, Aude Lemesle, Laurent Cotter, Donya El Akrouti, Virginie Redeker, Mathilde Louçã, Hélène Jean-Jacques, Yuanjing Liu, Abbie Maguire, Naoki Iwamoto, Michael Byrne, Chandra Vargeese, Sophie Lenoir, Frédéric Saudou, and Anselme L. Perrier

Table S1. Differentially expressed proteins identified by quantitative proteomic profiling following pan-allelic or allele-selective HTT ASO treatment in 109Q iPSC-derived cortical neurons.

See excel file Table S1.xlsx

Table S2. Antibodies used for immunostaining and Western blot

Target	Supplier	Application	Dilution	Reference
TBR1	Abcam	IF	1:500	ab31940
MAP2	Abcam	IF	1:500	ab5392
GABA	Sigma-Aldrich	IF	1:4000	A2052
cFos	Abcam	IF	1:500	2H2
EGR1	Cell Signaling	IF	1:500	15F7
GFAP	Millipore	IF	1:1000	AB5541
VLGUT2	Synaptic System	IF	1:500	135403
HOMER	Synaptic System	IF	1:500	160006
BASSOON	Abcam	IF	1:500	ab82958
Huntingtin	Cell Signaling	WB	1:1000	D7F7
Huntingtin	Ozyme	WB	1:1000	5656S
Calnexin	Sigma-Aldrich	WB	1:2500	C4731
GluA1	Millipore	WB	1:1000	AB1504
Vinculin	Cell Signaling	WB	1:10000	V9131
pAKT	Cell Signaling	WB	1:1000	40585
pERK	Cell Signaling	WB	1:2000	9106
AKT	Cell Signaling	WB	1:1000	9272
ERK	Cell Signaling	WB	1:1000	9102

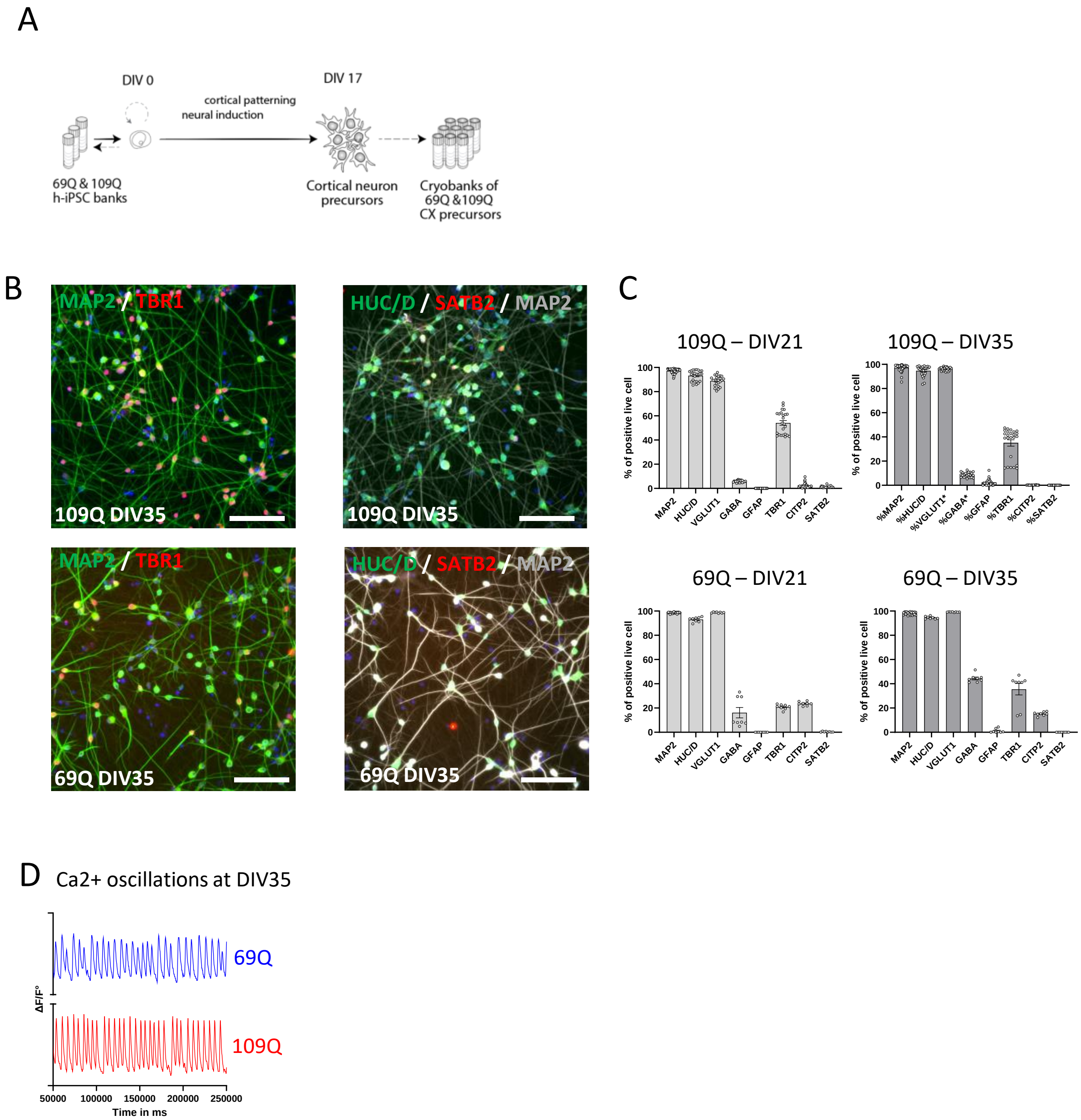

Figure S1. Characterization of human iPSC-derived neuronal population.

(A) Schematic of the differentiation. HD iPSCs carrying 69 or 109 CAG repeats were differentiated into cortical neurons.

(B) Immunostaining and (C) quantification of different markers of neuronal (MAP2, GABA), glial (GFAP), and cortical identities (TBR1) of 109Q iPSC- and 69Q-iPSC-derived cells at DIV21 and DIV35 after seeding of neuron precursor. Data are mean ± SEM; Scale bar: 50µm.

(D) Representative 200-second traces from the 10-min recordings of calcium oscillations in wells with 109Q or 69Q human cortical neuronal networks at DIV35 (synchronized calcium transient).

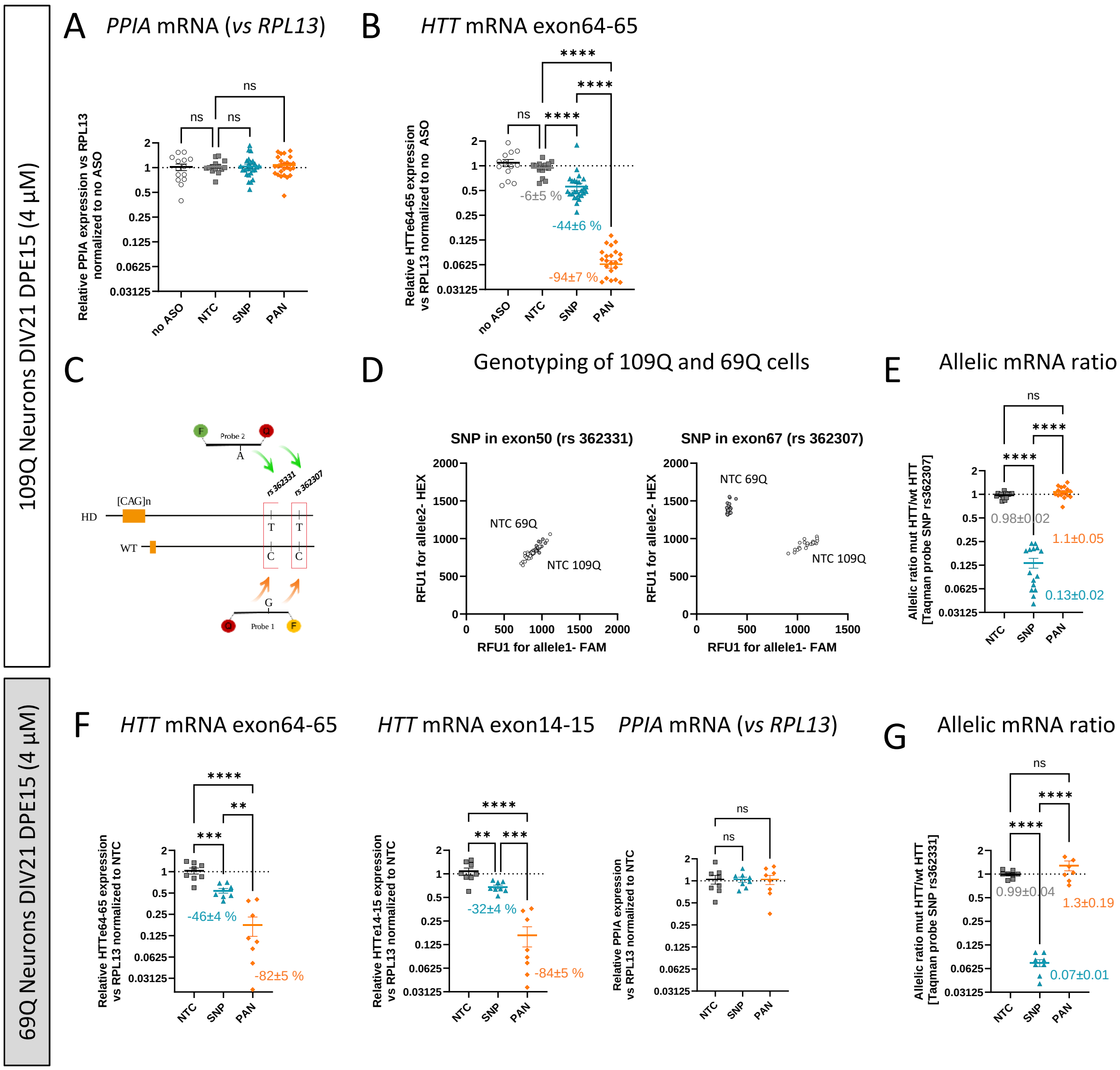

Figure S2. *HTT* mRNA-lowering efficacy and allele selectivity of ASOs in HD iPSC-derived cortical neurons.

(A) qRT–PCR analysis of *PPIA* relative to *RPL13* at DIV21–dpe15 showing stable expression of this second reference gene relative to *RPL13*.

(B) qRT–PCR analysis of spliced *HTT* transcripts (exons 64–65) at DIV21–dpe15 following treatment with NTC, PAN, or SNP ASOs.

(C) Schematic of TaqMan probe binding on both SNPs on either allele and allele-specific qPCR analysis.

(D) Genotyping of 109Q and 69Q lines for SNP rs362331 and rs362307. Distribution of the maximum fluorescence intensity of each probe after qRT-PCR of multiple 109Q and 69Q cDNA samples. Both 109Q and 69Q lines were heterozygous for SNP rs 362331, whereas only the 109Q line was heterozygous for SNP rs362307.

(E) Allelic ratio of mut- and wt-*HTT* mRNA in DIV21–dpe15 109Q neurons calculated based on the Ct difference of both probes for the same sample ($R_{mut/wt} = 2^{(-\Delta Ct)}$), normalized to the ratio of control samples.

(F) qRT–PCR analysis of spliced *HTT* transcripts (exons 14–15) at DIV21–dpe15 in 69Q neurons following treatment with NTC, PAN, or SNP ASOs. qRT–PCR analysis of *PPIA* relative to *RPL13* at DIV21–dpe15 in 69Q neurons.

(G) Allelic ratio of mut- and wt-*HTT* mRNA of DIV21–dpe15 neurons calculated based on the Ct difference of both probes for the same sample ($R_{mut/wt} = 2^{(-\Delta Ct)}$), normalized to the ratio of control samples.

Data are mean ± SEM; One-way ANOVA with post hoc test; p-values: *; <0.05; ** = 0.01; *** <0.001; **** < 0.0001.

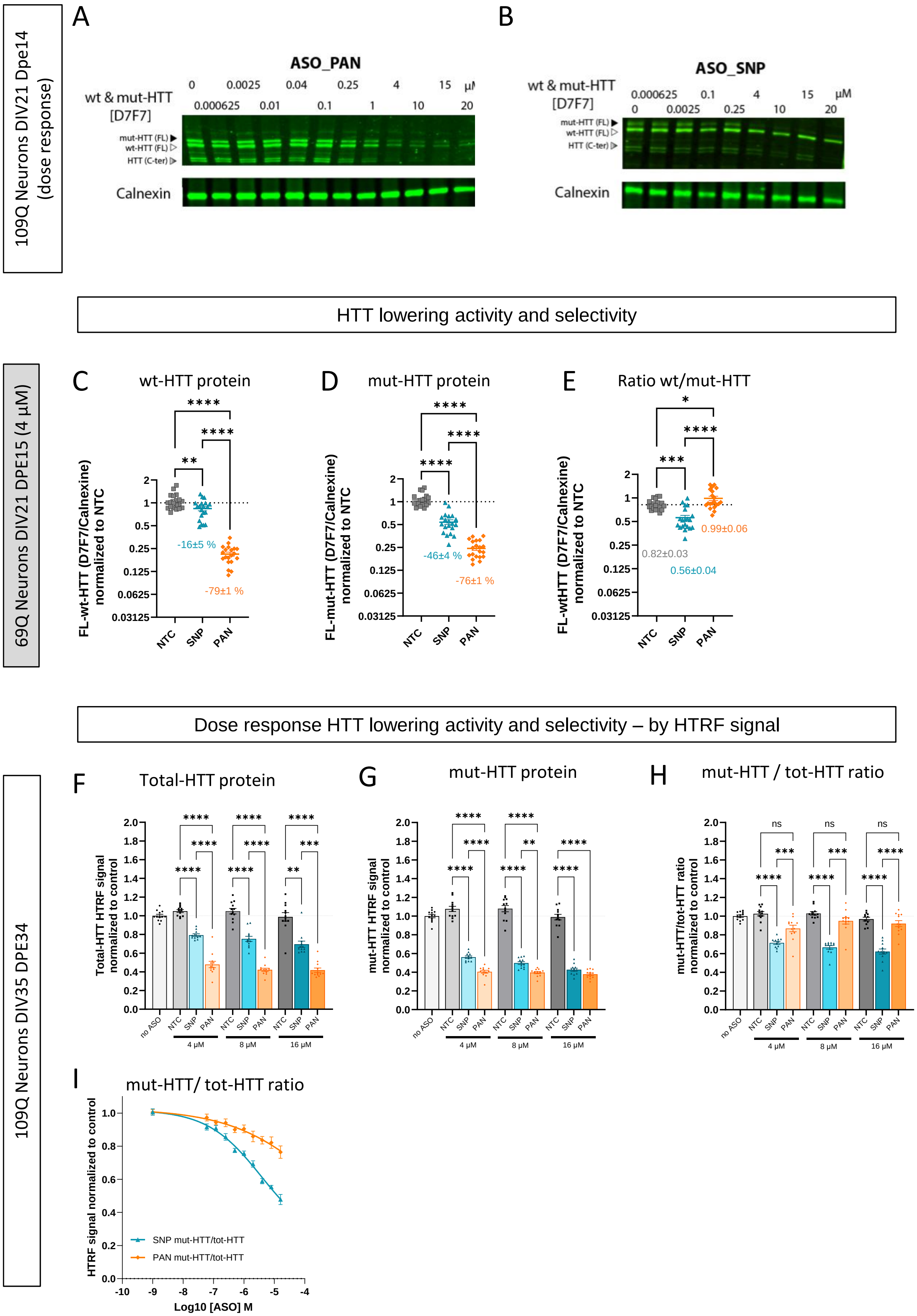
109Q Neurons DIV21 Dpe14 (dose response)
A
ASO_PAN
wt & mut-HTT [D7F7]
0 0.000625 0.0025 0.01 0.04 0.1 0.25 1 4 10 15 20 µM
mut-HTT (FL)
wt-HTT (FL)
HTT (C-ter)
Calnexin
B
ASO_SNP
wt & mut-HTT [D7F7]
0 0.000625 0.0025 0.1 0.25 4 10 15 20 µM
mut-HTT (FL)
wt-HTT (FL)
HTT (C-ter)
Calnexin
HTT lowering activity and selectivity
69Q Neurons DIV21 DPE15 (4 µM)
C
wt-HTT protein
FL-wt-HTT (D7F7/Calnexine) normalized to NTC
-16±5 %
-79±1 %
NTC
SNP
PAN
D
mut-HTT protein
FL-mut-HTT (D7F7/Calnexine) normalized to NTC
-46±4 %
-76±1 %
E
Ratio wt/mut-HTT
FL-wtHTT (D7F7/Calnexine) normalized to NTC
0.82±0.03
0.56±0.04
0.99±0.06
Dose response HTT lowering activity and selectivity – by HTRF signal
109Q Neurons DIV35 DPE34
F
Total-HTT protein
Total-HTT HTRF signal normalized to control
G
mut-HTT protein
mut-HTT HTRF signal normalized to control
H
mut-HTT / tot-HTT ratio
mut-HTT/tot-HTT ratio normalized to control
no ASO
4 µM
8 µM
16 µM
I
mut-HTT/ tot-HTT ratio
HTRF signal normalized to control
SNP mut-HTT/tot-HTT
PAN mut-HTT/tot-HTT
Log10 [ASO] M

Figure S3. HTT full-length protein-lowering efficacy and allele selectivity of ASOs in HD iPSC-derived cortical neurons.

(A-B) Representative western blot images of wild-type and mutant full-length and C-terminal fragment of HTT protein (D7F7 antibody) and Calnexin in 109Q neurons (DIV21-dpe15) exposed once to 0-20 µM of PAN or SNP ASO.

(C-E) Western blots and quantification of full-length HTT protein (D7F7 antibody) normalized to Calnexin in 69Q neurons at DIV21-dpe15 following treatment with NTC, PAN, or SNP ASOs.

(F-I) HTRF assays for total and mut-HTT protein at DIV35–dpe34 of 109Q neurons exposed to 4, 8 or 16 µM of ASOs.

Data are mean ± SEM ; One-way ANOVA with post hoc test; p-values: *; <0.05; ** = 0.01; *** <0.001; **** < 0.0001.

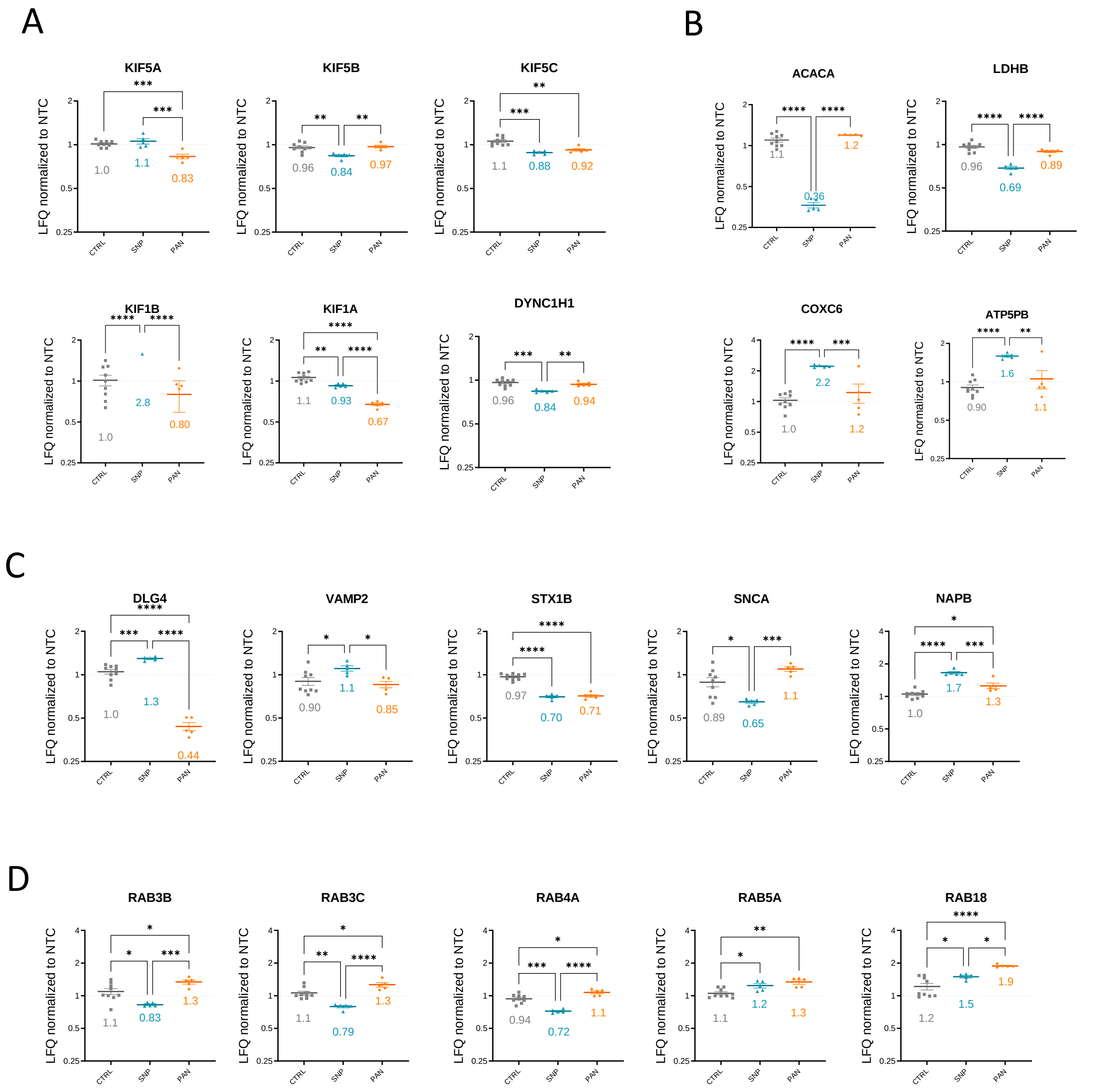

Figure S4. Proteomic signature of allele-selective versus pan-allelic HTT lowering in human HD cortical neurons.

Label free quantification signals normalized to NTC treated neurons for selected proteins involved in (A) cytoskeletal motors (KIF5A/B/C, KIF1A/B, DYNC1H1) essential for axonal transport, (B) metabolic enzymes (ACACA, LDHB, COX6C, ATP5PB) involved in ATP production, (C) synaptic proteins (DLG4/PSD-95, VAMP2, STX1B, SNCA, NAPB) mediating vesicle fusion and neurotransmission, and (D) Rab GTPases (RAB3B/C, RAB4A, RAB5A, RAB18) regulating vesicular trafficking. In the graphs, the condition labeled "CTRL" represents the combined control samples (NTC-treated and no-ASO), whereas normalization was performed exclusively relative to the NTC-treated group.

Data are mean ± SEM ; One-way ANOVA with post hoc test; p-values: *; <0.05; ** = 0.01; *** <0.001; **** < 0.0001.

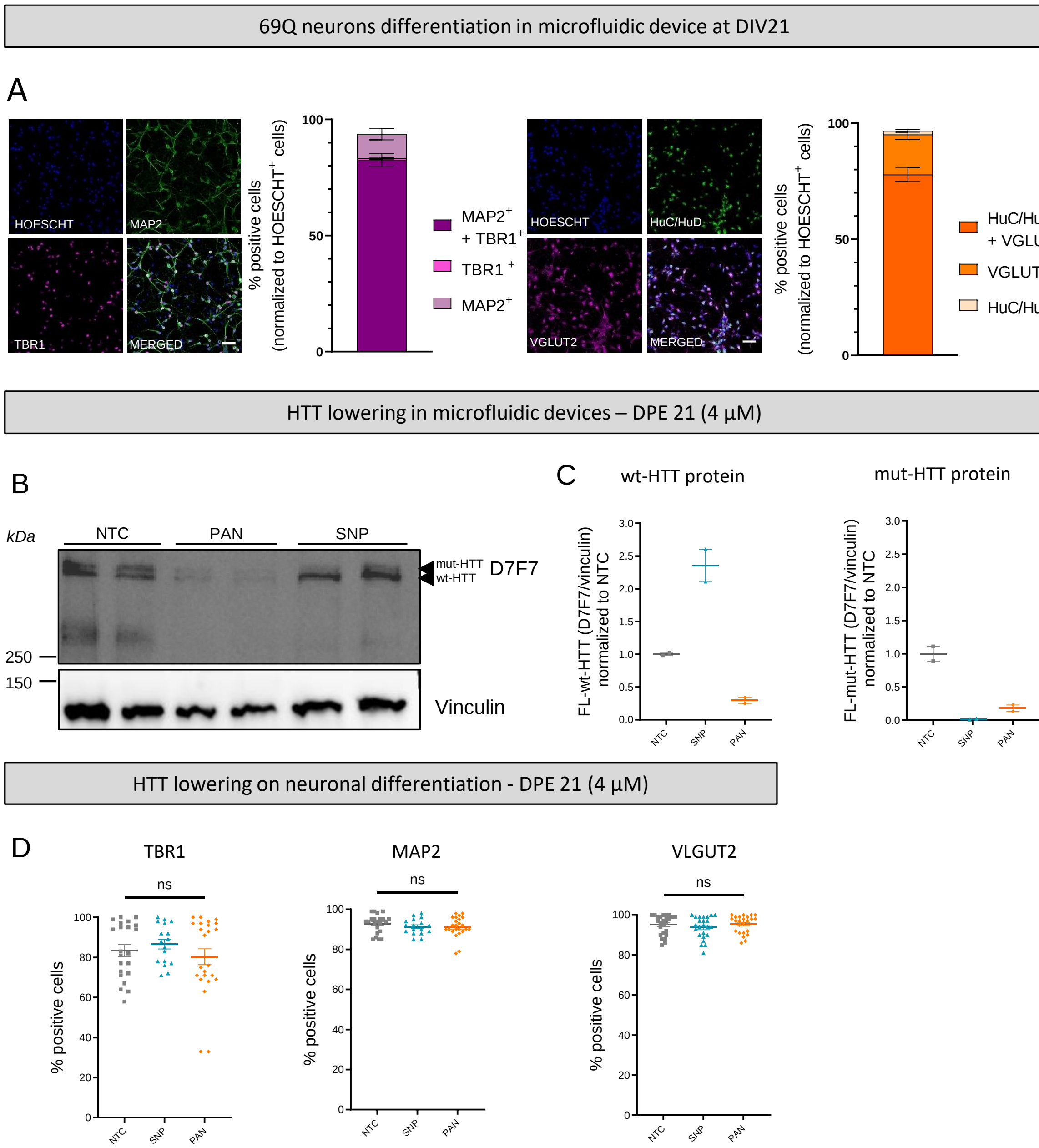

Figure S5. HTT silencing in human 69Q corticocortical neuronal networks-on-chip.

(A) Representative immunofluorescence images of 69Q iPSC-derived cortical neurons at DIV21, showing neuronal (HuC/D, MAP2) and cortical glutamatergic identity (TBR1, VGLUT2) markers.

(B-C) Western blot validation of *HTT* silencing following exposure of 69Q cells at DIV1 to 4 µM NTC, PAN or SNP ASOs for 20 days. Representative blots (B) and quantification of wt-HTT and mut-HTT (C) normalized to Vinculin.

(D) Quantification of TBR1^{+}, MAP2^{+}, and VGLUT2^{+} populations.

Data are mean ± SEM; n = 3–4 independent networks per condition; one-way ANOVA with Tukey's post hoc test;

; p-values: *; <0.05; ** = 0.01; *** <0.001; **** < 0.0001. Scale bar: 50 µm.

BDNF containing vesicles traffic – 69Q Neurons DIV21 – Dpe 20 (4µM)

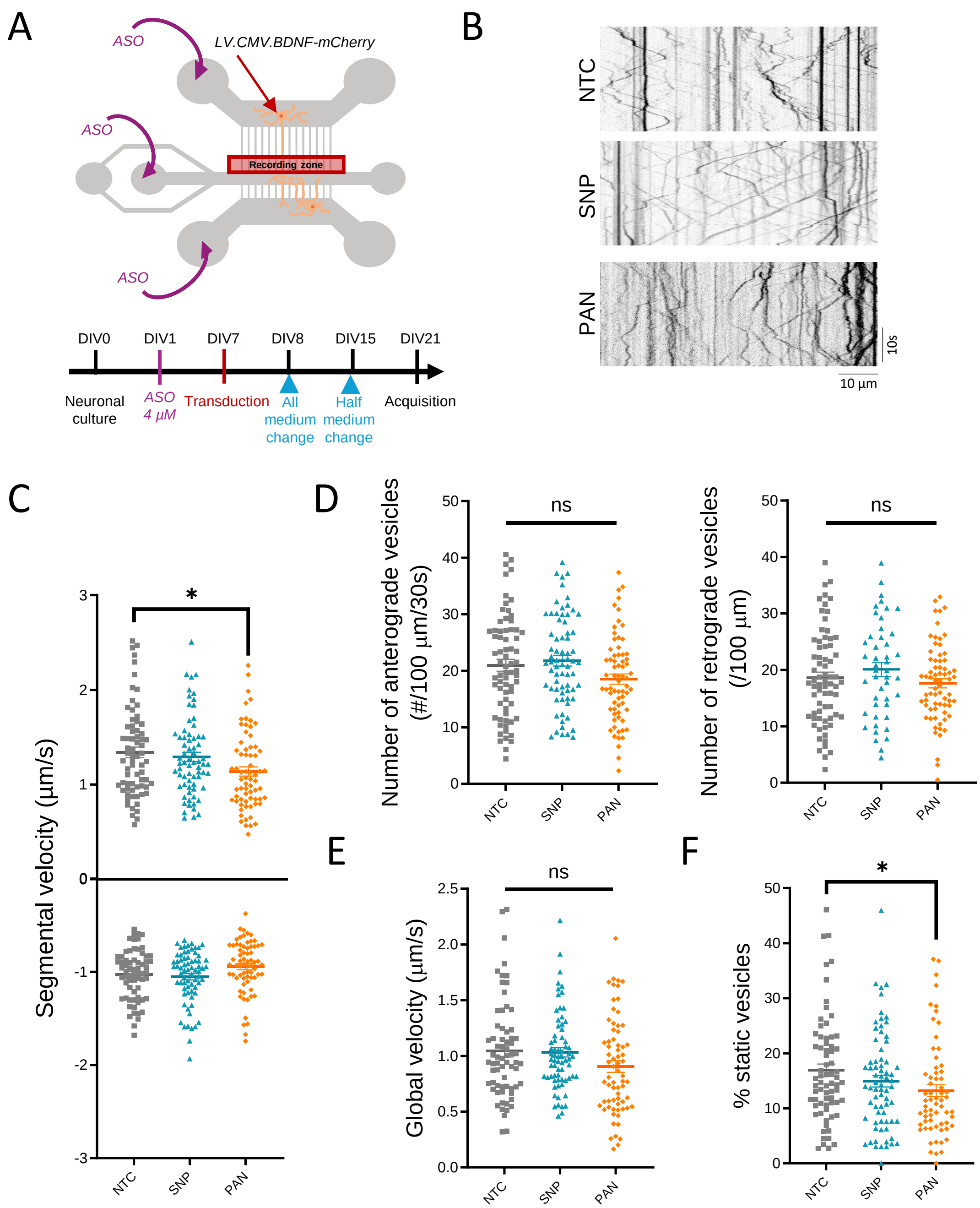

Figure S6. BDNF-mCherry vesicle transport following selective or pan-allelic HTT silencing .

(A) Schematic of the microfluidic device (MFD) used to reconstruct human corticocortical networks and experimental timeline. 69Q iPSC-derived cortical neurons were treated at DIV1 with 4 µM NTC, PAN or SNP ASOs. At DIV7, presynaptic 69Q iPSC–derived cortical neurons were transduced with lentiviral BDNF–mCherry. Live imaging of distal axons within 500-µm microchannels was performed at DIV21.

(B) Representative kymographs showing BDNF–mCherry vesicle trajectories in neurons treated with NTC, PAN, or SNP ASOs.

(C–F) Quantitative analyses of BDNF–mCherry transport. (C) Segmental velocities of anterograde and retrograde vesicles. (D) Number of anterograde and retrograde vesicles per 100 µm of axon. (E) Global vesicle velocity. (F) Percentage of static vesicles.

Data represent mean ± SEM from three independent experiments (≥58 axons per condition). One-way ANOVA followed by Tukey's post hoc test (E, anterograde velocity in C) or Kruskal–Wallis test followed by Dunn's multiple comparison (C, retrograde velocity; D; F); *$p < 0.05$, **$p < 0.01$.

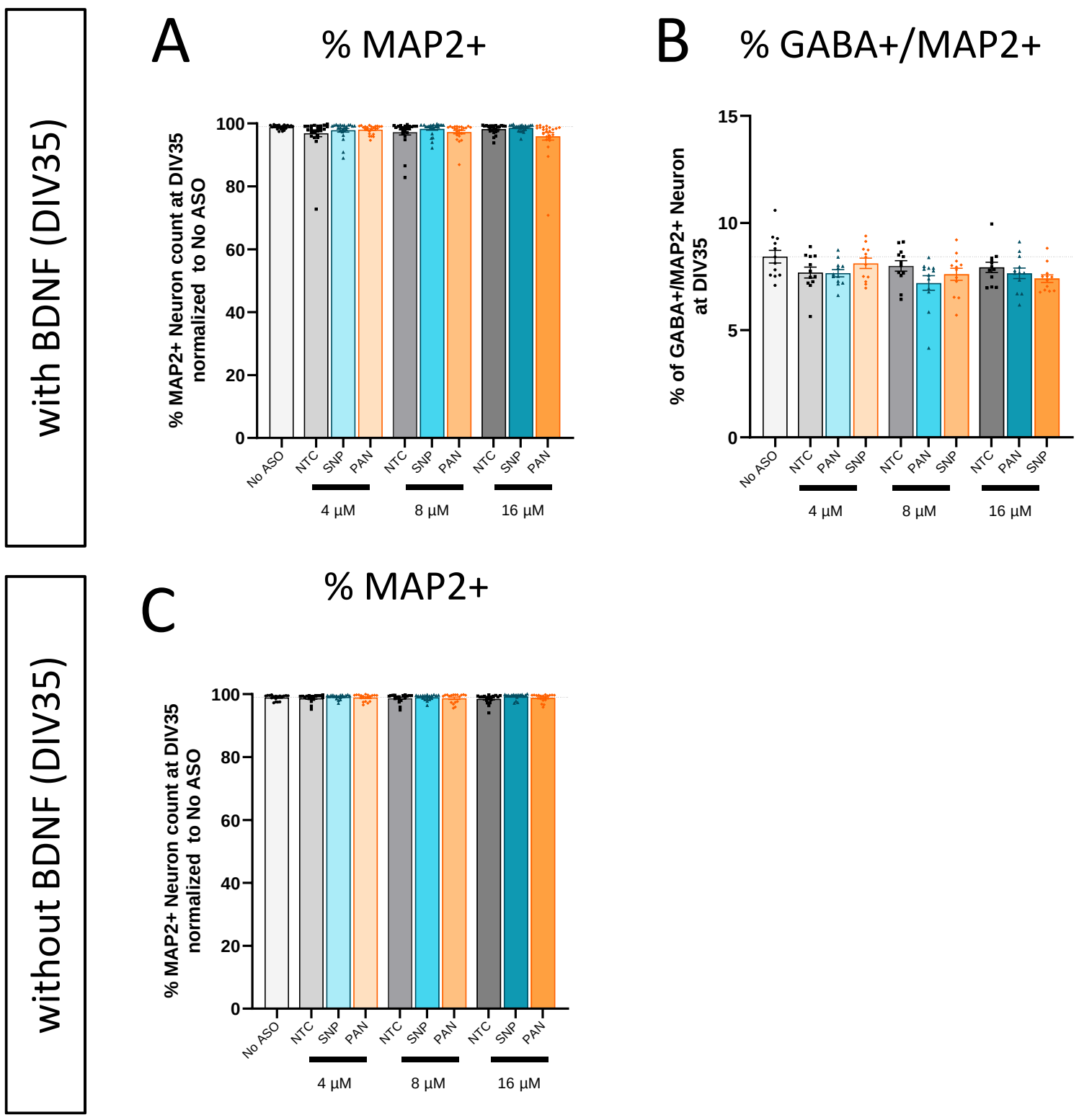

Figure S7. Effects of *HTT* silencing on neuronal subtype composition.

(A-B) Quantification of percentage of $MAP2^+$ and $GABA^+$ neurons in 109Q cortical cultures exposed once at DIV1 to different concentrations (0, 4, 8, 16 µM) of NTC (grey), SNP (teal), or PAN (orange) ASOs. Data are normalized to untreated controls. Cultures maintained with BDNF supplementation from DIV14 onward.

(C) Quantification of MAP2+ and GABA+ neurons in cultures without BDNF supplementation.

Data represent mean ± SEM from ≥3 independent experiments (n=6 cultures, 25 fields per condition). One-way ANOVA with Sidak's or Tukey's post hoc test.

Post-synaptic glutamate receptor and MAPK activation –
109Q Neurons DIV21 – Dpe 20 (4µM) in MFD

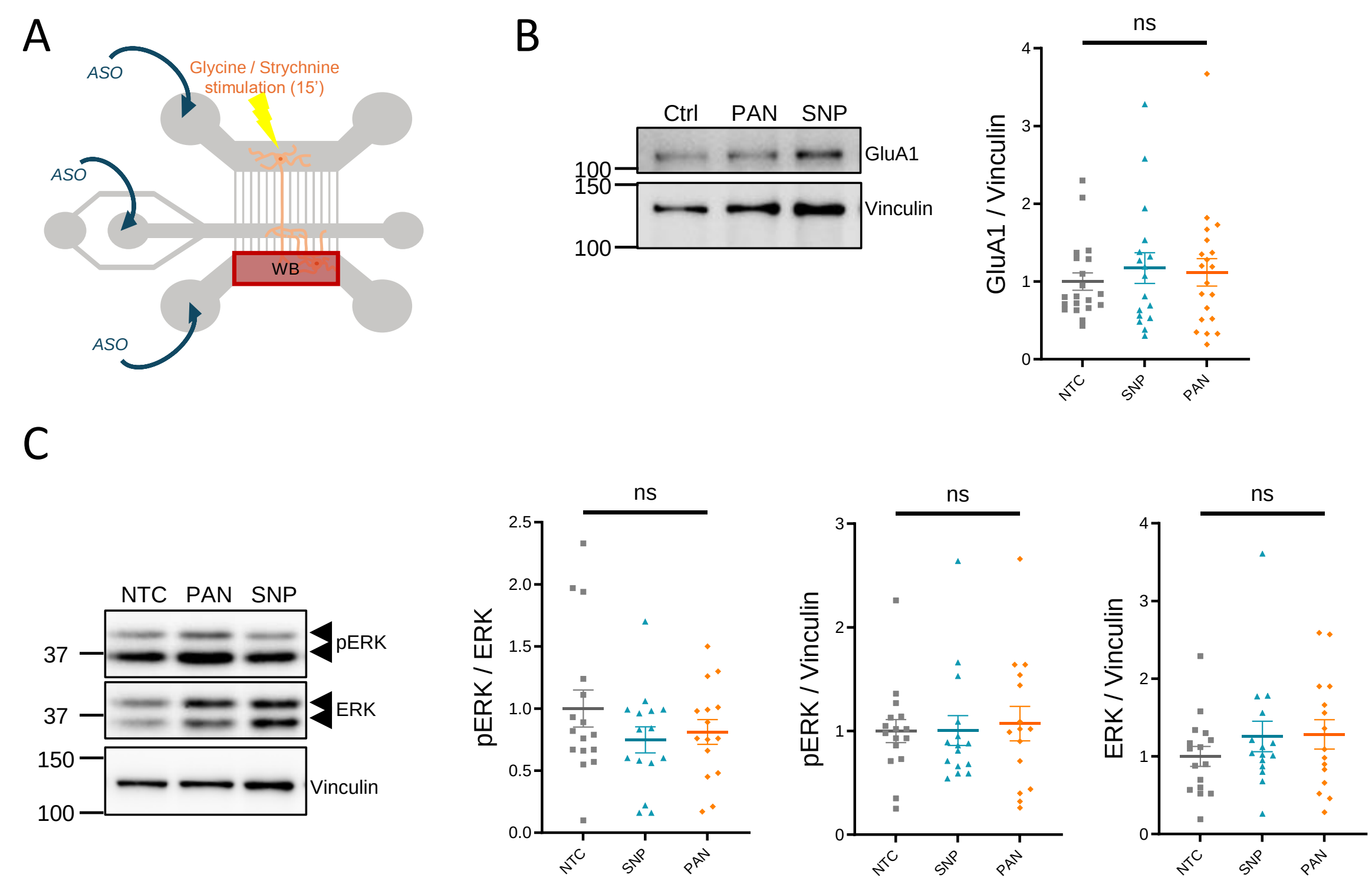

Figure S8. Postsynaptic glutamate receptor and MAPK signaling following selective or pan-allelic *HTT* silencing.

(A) Schematic of the microfluidic device (MFD) used to reconstitute human corticocortical networks, illustrating presynaptic stimulation with glycine/strychnine solution and postsynaptic protein extraction zone after 15 min stimulation performed at DIV21. Presynaptic neurons derived from 109Q iPSCs were treated at DIV1 with 4 µM NTC (grey), SNP (teal), or PAN (orange) ASOs.

(B) Western blots analysis of GluA1 expression normalized to Vinculin in 109Q neuronal networks treated with NTC, SNP, or PAN ASOs.

(C) Western blot analysis of pERK and total ERK expression normalized to Vinculin in 109Q neuronal networks treated with NTC, SNP, or PAN ASOs.

Each point represents protein extracts from one MFD (postsynaptic compartment only); four independent experiments, n = 12–16 per condition. Kruskal–Wallis or one-way ANOVA with appropriate post hoc tests; ns, not significant.

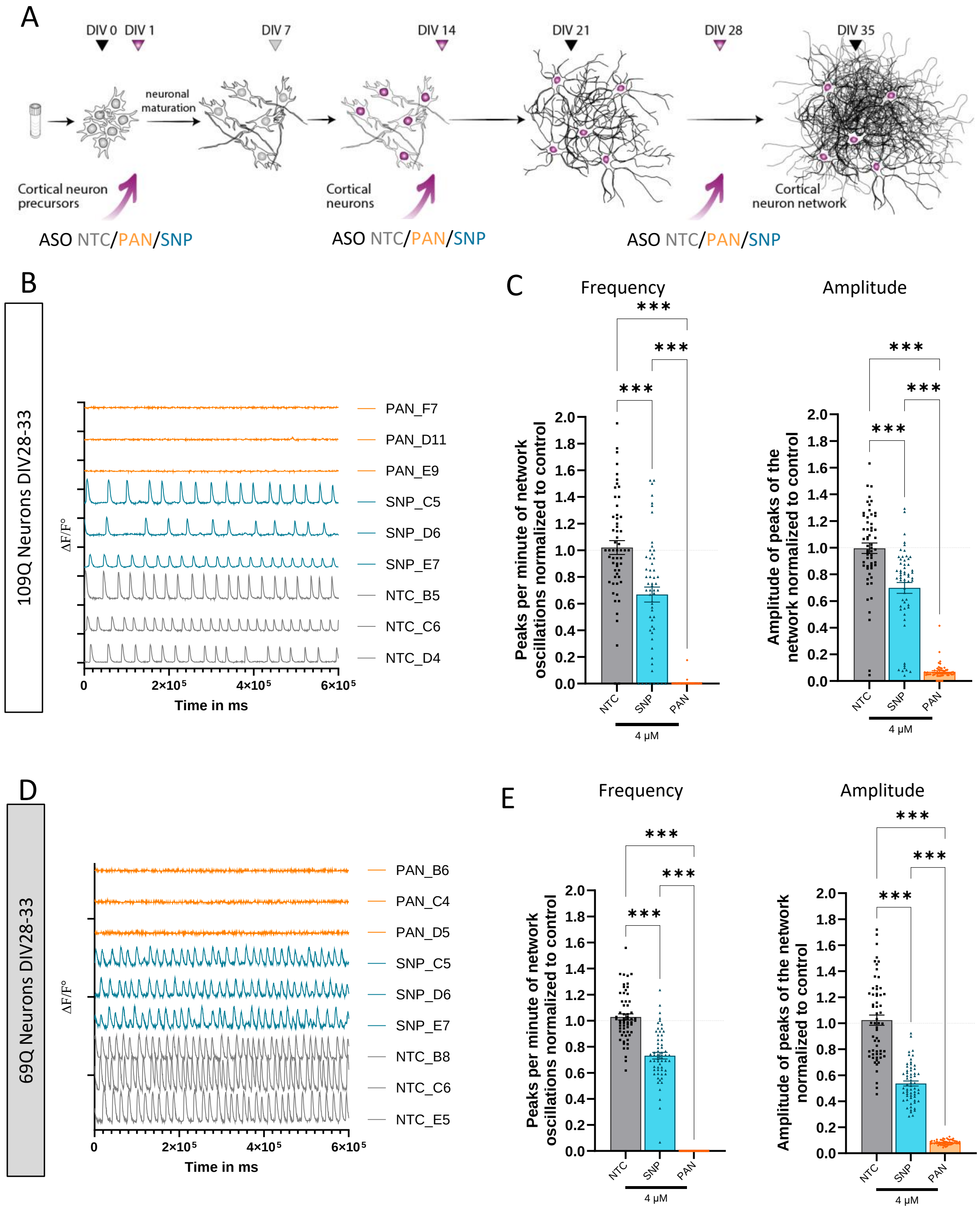
A
DIV 0
DIV 1
DIV 7
DIV 14
DIV 21
DIV 28
DIV 35
neuronal maturation
Cortical neuron precursors
Cortical neurons
Cortical neuron network
ASO NTC/PAN/SNP
B
109Q Neurons DIV28-33
ΔF/F°
PAN_F7
PAN_D11
PAN_E9
SNP_C5
SNP_D6
SNP_E7
NTC_B5
NTC_C6
NTC_D4
Time in ms
C
Frequency
Amplitude
Peaks per minute of network oscillations normalized to control
Amplitude of peaks of the network normalized to control
NTC
SNP
PAN
4 µM
D
69Q Neurons DIV28-33
PAN_B6
PAN_C4
PAN_D5
SNP_C5
SNP_D6
SNP_E7
NTC_B8
NTC_C6
NTC_E5
E

Figure S9. Effects of HTT silencing on network synchronization across genotypes and repeated dosing paradigms.

(A) Schematic of the differentiation and repeated treatment workflow. Synchronized calcium transients in cortical neuronal networks derived from 109Q or 69Q human iPSCs after repeated exposure (DIV1, 14 and 28) to 4 µM NTC (grey), SNP (teal), or PAN (orange) ASOs. Network activity was recorded at DIV28 or DIV33 using Cal-520 fluorescence imaging.

(B & D) Representative 10 min traces of calcium oscillations from 109Q and 69Q neuronal networks.

(C & E) Quantification of normalized oscillation frequency and amplitude.

Data represent mean ± SEM from three independent experiments (20 cultures wells per condition); one-way ANOVA with Sidak's multiple comparisons; *$p < 0.05$, **$p < 0.01$, ***$p < 0.001$.

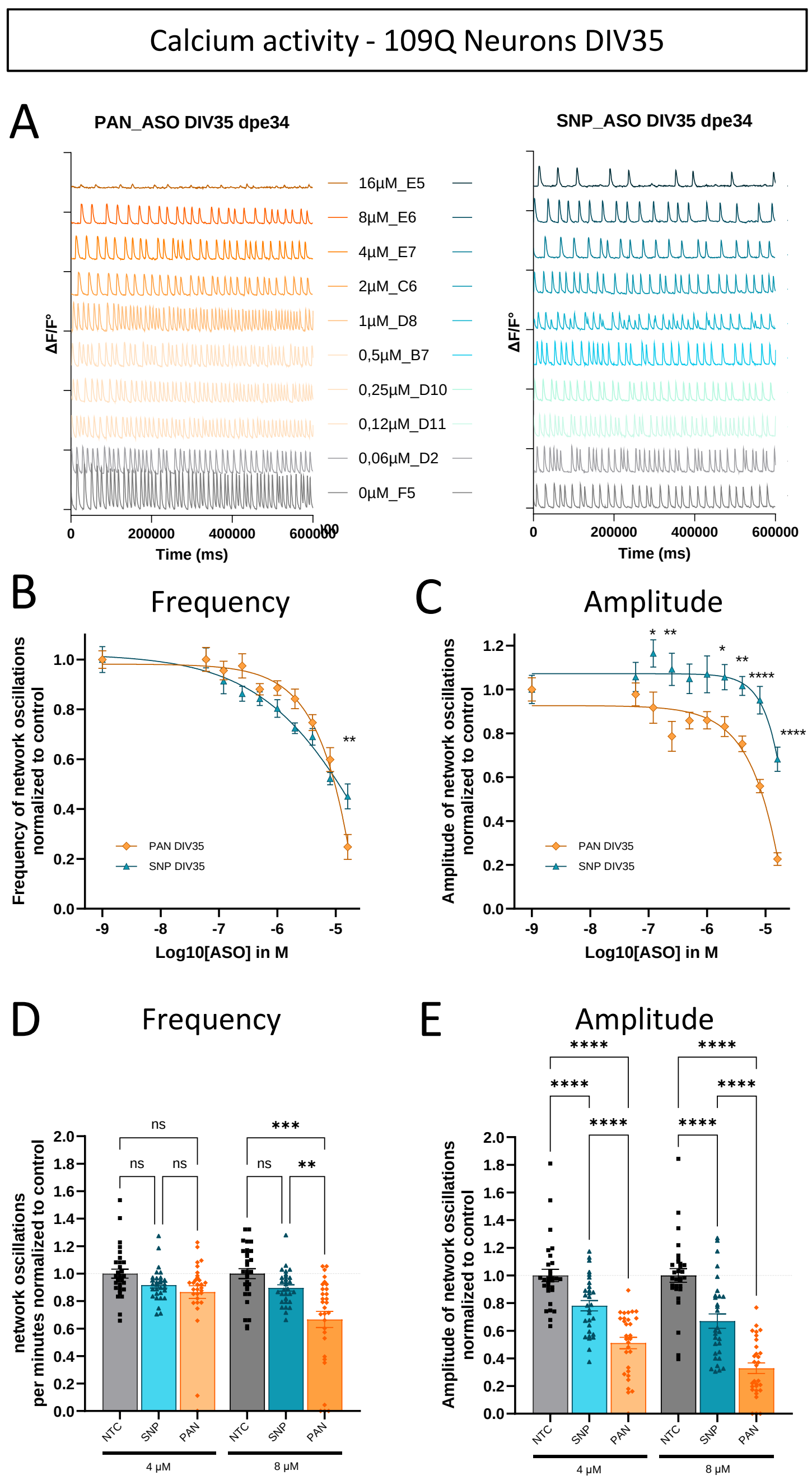

Figure S10. Sustained network activity following selective or pan-allelic *HTT* silencing.

(A–E) Spontaneous calcium oscillations in 109Q cortical networks recorded at DIV35 following a single exposure to 0–16 µM NTC (grey), SNP (teal), or PAN (orange) ASOs at DIV1.

(A) Representative 10 min traces of calcium oscillations.

(B & C) Dose–response curves showing normalized frequency (B) and amplitude (C) of network oscillations.

(D & E) Quantification at 4 µM and 8 µM ASOs relative to NTC. Despite partial loss of SNP ASO efficacy at this time point, network oscillations remained significantly more preserved than with PAN ASO treatment.

Data represent mean ± SEM from three independent experiments (six wells per condition); one-way ANOVA with Sidak's multiple comparisons; *p < 0.05, **p < 0.01, ***p < 0.001, ****p<0.0001***p < 0.001.
